# Systematic Ligand Modification Tunes the Stability and Reactivity of Copper Complexes for the Electrocatalytic Reduction of Carbon Dioxide

**DOI:** 10.1021/jacs.6c11205

**Published:** 2026-09-08

**Authors:** Ramamoorthy Ramasubramanian, Charasee Laddika Dayawansa, Jyun-Chi Lee, Rui-Ze Xu, Jiun-Shian Shen, Tzu-Hsien Tseng, Tsz-Fai Leung, Tiow-Gan Ong, Glenn P. A. Yap, Vincent C.-C. Wang

**Affiliations:** † Department of Chemistry, 34874 National Sun Yat-sen University, Kaohsiung 80424, Taiwan R.O.C.; ‡ Department of Chemistry, 34910 National Taiwan University, Taipei 10617, Taiwan, ROC; § Instrument Center, 63284 National Chung Hsing University, Taichung 402202, Taiwan R.O.C.; ∥ The School of Science and Engineering, 407605 The Chinese University of Hong Kong, Longgong, Shenzhen, Guangdong 518172, China; ⊥ Department of Chemistry and Biochemistry, 5972 University of Delaware, Newark, Delaware 19716, United States; # Green Hydrogen Research Center, National Sun Yat-Sen University, Kaohsiung 80424, Taiwan R.O.C.

## Abstract

Compared to first-row transition metal complexes, such as Mn, Fe, Co, and Ni, the development of molecular copper electrocatalysts for CO_2_ reduction has been plagued by the instability of low-valent Cu intermediates, which are prone to undergo demetallation or aggregation. A series of rational and systematic ligand modifications was employed to transform a CO_2_-fixing yet electrochemically unstable pyridine-2,6-dicarboxamide copper complex into a stable electrocatalytic system. Guided by the Hard–Soft Acid–Base principle, incorporation of a thioether donor into the nitrogen-based pincer framework stabilized Cu­(I) intermediates, thereby promoting reversible electron transfer and mitigating the instability of the hard nitrogen-donor environment. Furthermore, the introduction of a macrocyclic structure not only enhances the overall stability of the complex but also minimizes structural reorganization to support the redox reversibility of the redox-active ligand. The resulting complex mediated the electroreduction of CO_2_ to CO through an alternating sequence of electron-transfer (E) and chemical (C) steps (ECEC mechanism). The mechanistic studies identified the key CO_2_-bound and protonated HCO_2_-bound copper intermediate species using a combination of spectroscopic tools, complementary *in situ* electrochemical methods, and isotopic labeling. In addition, comparative experimental and computational analyses of the copper complexes further revealed the factors governing catalyst stability. These results demonstrate that the interplay among the coordination sphere, redox-active ligand, and ligand rigidity dictates the electrocatalytic performance and stability of molecular copper complexes. This work provides design principles and mechanistic insights applicable to both homogeneous electrocatalysts and copper-complex-derived heterogeneous systems for CO_2_ reduction.

## Graphical abstract

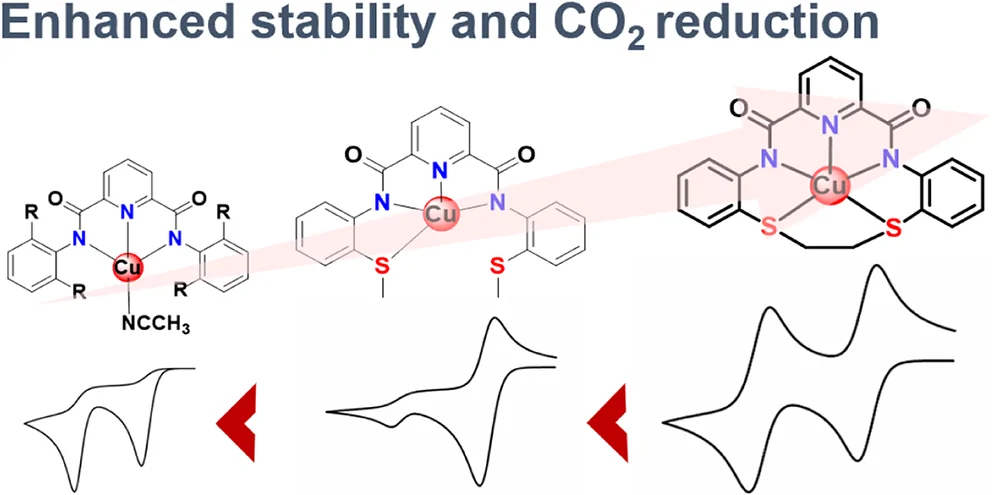

## Introduction

The electrocatalytic conversion of carbon dioxide (CO_2_) into fuels or value-added chemicals represents a key strategy for mitigating the environmental impact of increasing atmospheric CO_2_ levels.^1,2^ The development of non-noble metal electrocatalysts for CO_2_ reduction has drawn huge attention. One prominent example is copper-based materials.^3,4^ Metallic copper has been widely studied and employed as a heterogeneous electrocatalyst for CO_2_ conversion to a diverse range of products, including hydrocarbons and oxygenates.^3^ The reactivity and selectivity of these materials are highly sensitive to surface modifications. For example, recently, the addition of organic molecular additives to metallic copper systems has led to pronounced changes in reactivity and selectivity, underscoring the importance of atomic-level mechanistic understanding for tuning catalytic performance.^5–7^ A molecular complex offers a synthetically tunable platform that enables precise structural modifications and in-depth mechanistic interrogation through a broad range of spectroscopic techniques.

However, compared with first-row transition-metal complexes such as Mn, Fe, Co, and Ni, which have been extensively explored to promote CO_2_ reduction, molecular copper-based counterparts are comparatively underdeveloped.^2,8–12^ This limitation arises from the intrinsic instability of low-valent copper intermediates. The Cu­(I) state (d^10^) lacks ligand-field stabilization, and further reduction often produces Cu(0), which is prone to undergo demetalation and aggregation. For example, nitrogen-rich ligands, porphyrins, polypyridyls, and N-heterocyclic carbene frameworks exacerbate this problem by generating electron-rich environments that could destabilize low-valent copper intermediates and promote decomposition under electrochemical CO_2_ reduction conditions.^8,9,13–18^ Consequently, many reported copper complexes either exhibit poor catalytic stability or serve only as precursors to generate new types of heterogeneous Cu materials. Nevertheless, the demetalation pathway of copper-complex-derived materials, including the influence of ligand environments, remains unclear.

To address these challenges and issues, two levels of ligand environment were targeted in this research. The primary coordination sphere is directly modified to tune the electronic structure and stability of the copper center.^19,20^ In addition, a redox-active ligand framework beyond the metal center is employed to enhance the cooperative behavior between the metal center and the ligand.^21^ We hypothesize that stabilization of a low-valent copper center can be achieved through ligand designs based on the Hard–Soft Acid–Base (HSAB) principle. Specifically, incorporating a soft donor atom, such as sulfur, may stabilize Cu­(I) species and enhance CO_2_ reactivity. This concept is inspired by Type I copper proteins, which employ a thiolate or thioether coordination environment to facilitate a Cu­(II)/Cu­(I) redox event.^22,23^ Previous studies have introduced anionic thiolate donors into ligand frameworks for Ni and Co complexes to improve performance in CO_2_ reduction, demonstrating enhancements in activity for related systems.^24–27^ These reports complement our strategy of integrating neutral thioether donors to stabilize low-valent copper. Furthermore, a redox non-innocent ligand can serve as an electron reservoir, facilitating the multielectron reaction while preventing excessive charge localization on the metal center alone.

Building on these principles, we employed pyridine-2,6-dicarboxamide ligands, as shown in Figure 1, developed by Holm and coworkers, as a starting ligand scaffold for designing new copper complexes. The pincer-type complex [**1** ^**Me**^ **-OH**], bearing hydroxide ligands and derived from the Holm group, has been shown to mediate CO_2_ fixation through nucleophilic attack by the metal-hydroxide center (Cu or Ni), forming bicarbonate.^28–30^ This CO_2_ fixation pathway mirrors the mechanism found in Ni,Fe-containing carbon monoxide dehydrogenase (CODH), which is one of the most efficient CO_2_ reducing electrocatalysts.^31,32^ In addition, the ligand scaffold consists of a rigid pincer structure and possesses a pyridine core that is known to be a redox-active ligand.^21,33,34^ Notably, the related complex **1** ^**Me**^, as shown in Figure 1, was recently shown to exhibit photocatalytic activity for the conversion of CO_2_ to CO.^35^ These findings highlight the potential of **1** ^**Me**^ as a promising complex for integrating both CO_2_ fixation and reduction within a single active site as an electrocatalyst. Therefore, we started with the evaluation of the electrocatalytic properties of CO_2_ reduction mediated by complex **1** ^**Me**^ and a bulkier analogue, **1** ^*i* **Pr**^.

**Figure 1:**
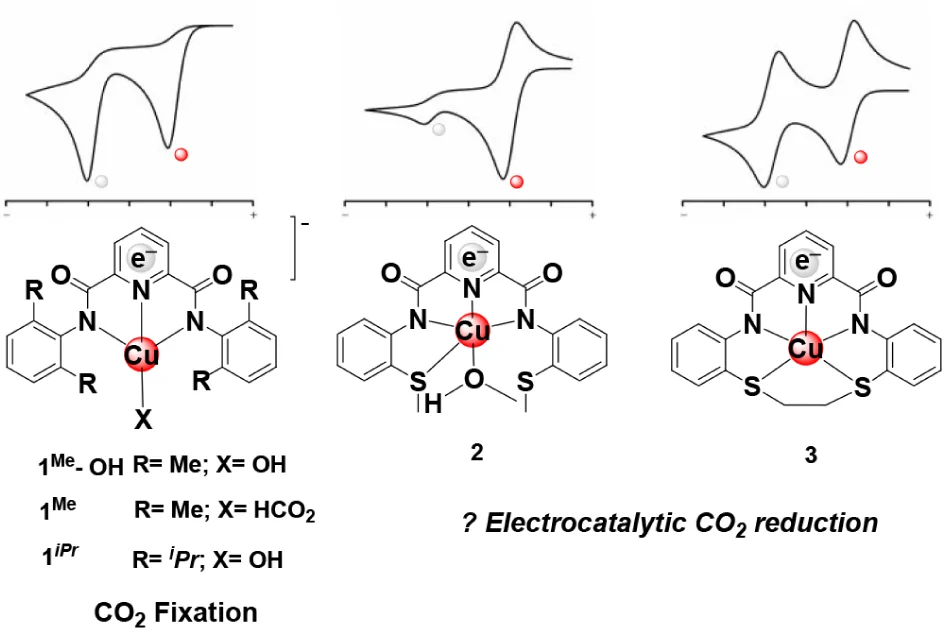
Copper complexes studied for CO_2_ fixation and reduction. The voltammograms illustrate the redox behavior found among the complexes.

To further enhance their stability and reactivity, we prepared two new copper­(II) complexes: an acyclic complex **2** and a macrocyclic complex **3**, both derived from pyridine-2,6-dicarboxamide frameworks modified with thioether moieties, as shown in Figure 1. These structural modifications introduce soft donor S atoms and further enhance ligand rigidity by cyclization. These systematic modifications of ligand frameworks allow evaluation of their impact on redox behavior, CO_2_ binding, and overall electrocatalytic performance. Further mechanistic studies revealed the ECEC pathway (E: electron transfer step; C: chemical step) for CO_2_ reduction to CO, with identification of the key CO_2_-bound and subsequently protonated intermediate, as well as the redox-active ligand that facilitates C–O bond cleavage. These systematic variations in ligand architecture demonstrated here reveal effective strategies to overcome the intrinsic instability of copper molecular complexes and provide guiding principles for the development of homogeneous electrocatalysts and copper complex-derived heterogeneous materials for electrocatalytic CO_2_ reduction.

## Results

### Electrocatalytic Performance of Pincer-Type Copper Complexes **1^Me^** and **1** ^***i*Pr**^

Following previous literature procedures,^30,35–37^ complexes **1** ^**Me**^ and **1** ^*i* **Pr**^ were prepared and evaluated as benchmarks. Both display UV-vis features consistent with reported CO_2_ fixation via hydroxide attack, and upon CO_2_ exposure, their spectra shift in agreement with bicarbonate formation (Figures S1–S2).^30^ To further explore the electrocatalytic CO_2_ reduction mediated by **1** ^**Me**^ and **1** ^*i* **Pr**^, electrochemical studies were performed in dimethylformamide (DMF). All potentials reported here are referenced against the Fc^^+^/0^ redox couple. Electrochemical studies under N_2_ reveal two irreversible reduction waves for **1** ^**Me**^ at −1.25 V and −1.76 V (Figure S3) and for **1** ^*i* **Pr**^ at −1.21 V and −1.91 V (Figure S4), assigned to the Cu­(II)/Cu­(I) redox couple and a ligand-centered redox event (*vide infra*). Under CO_2_, both complexes showed increased currents after the second redox peak, but rinse tests indicated catalyst decomposition (Figures S3, S4). Analysis of the electrode after bulk electrolysis indicated the deposition of heterogeneous copper material on the electrode surface, and additional X-ray photoelectron spectroscopy (XPS) revealed the formation of copper oxide (Figures S5, S6). Notably, the steric protection offered by the isopropyl substituents in **1** ^*i* **Pr**^ does not improve stability compared to **1** ^**Me**^. Collectively, these results highlight that nitrogen-only scaffolds fail to stabilize reduced copper species during electrocatalysis.

### Synthesis and Characterization of the Thioether-Modified Complexes **2** and **3**

To address the instability of the copper pyridine-2,6-dicarboxamide complex, a soft thioether donor was incorporated into the ligand framework, as shown in Figure 1. In addition, both acyclic and macrocyclic ligands were prepared to investigate the reactivity and stability of the resulting copper complexes. The single-crystal X-ray diffraction structures of the acyclic complex **2** and the macrocyclic complex **3** were successfully obtained, as illustrated in Figure 2. Both complexes exhibit a distorted, five-coordinate, square-pyramidal geometry around the copper centers, with τ_5_ parameter values of 0.07 for complex **2** and 0.19 for complex **3**, respectively. In complex **2**, the copper ion is coordinated by three nitrogen atoms, a sulfur atom from the ligand, and an exogenous methanol solvent molecule through its oxygen atom.

**Figure 2:**
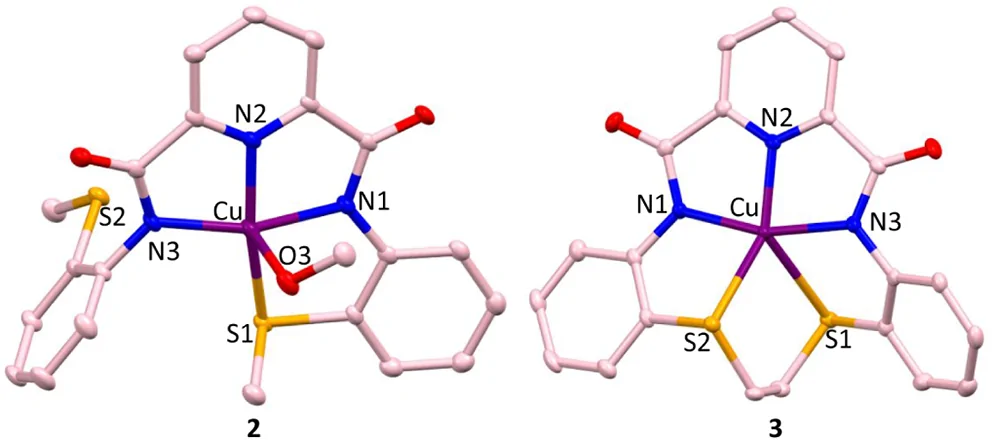
Molecular diagrams of complexes **2** and **3** with atoms depicted as 50% probability ellipsoids with colors corresponding to atom identities: C = pink; Cu = purple; S = orange; O = red; and N = blue. Hydrogen atoms are omitted for clarity.

In contrast, complex **3** features coordination by three nitrogen and two sulfur atoms from the macrocyclic ligand. The Cu–N_Py_ bond lengths in complexes **2** [1.905(2) Å] and **3** [1.914(18) Å] are slightly shorter than those reported for **1** ^**Me**^ and **1** ^*i* **Pr**^ (∼1.92 Å).^30,36,37^ The Cu–S bond lengths in complex **3** are Cu–S(1) = 2.733(6) Å and Cu–S(2) = 2.378(6) Å, compared to a shorter Cu–S(1) bond of 2.281(7) Å in complex **2**. These distances are longer than those characteristic of Cu–S (thiolate) bonds found in Type 1 copper proteins (typically 2.07–2.20 Å), but are similar to the range reported for Cu–S (thioether) coordination in copper centers.^38,39^ The electron paramagnetic resonance (EPR) spectra of the two complexes, collected at 77 K in a DMF/Toluene mixture, were further employed to investigate the electronic structure and coordination environment of the copper center (Figure S7). Complex **2** displayed g values of g_*x*_ = 1.98, g_*y*_ = 2.04, and g_*z*_ = 2.29, while complex **3** shows slightly shifted g values (g_*x*_ = 1.95, g_*y*_ = 2.03, g_*z*_ = 2.23). The corresponding hyperfine coupling constants, derived from spectral simulations, were A_*x*_ = 15 MHz, A_*y*_ = 14 MHz, and A_*z*_ = 511 MHz for complex **2**, and A_*x*_ = 11 MHz, A_*y*_ = 17 MHz, and A_*z*_ = 515 MHz for complex **3**. These rhombic EPR signals are consistent with the distorted square pyramidal geometries observed in the crystal structures.

### The Reactivity of Complexes **2** and **3** toward CO_2_

Upon addition of CO_2_ to complexes **2** and **3** in DMF, unlike **1** ^**Me**^ **-OH** and **1** ^*i* **Pr**^, no significant change in the d–d transition region of the UV-vis spectra was observed (Figures S1–S2), consistent with the absence of metal–hydroxide coordination. For complex **2**, the addition of tetrabutylammonium hydroxide (TBAOH) shifted the absorption to 575 nm, with a corresponding color change from brown to green. Subsequent CO_2_ exposure caused a shift to 535 nm, indicating CO_2_ reactivity arising from the hydroxide-bound form of complex **2** (Figure S8).^40^ In contrast, complex **3** showed no discernible spectral changes upon CO_2_, even in the presence of TBAOH (Figure S9), suggesting no hydroxide interaction at the copper site.

### Electrocatalytic Properties of CO_2_ Reduction Mediated by Complex **2**

First, cyclic voltammetry measurements of complex **2** in the presence of N_2_ were performed in DMF to explore its redox behavior. Complex **2** also displayed two redox events. Nevertheless, compared to **1** ^**Me**^ and **1** ^*i* **Pr**^, the first redox peak became a reversible wave observed at *E*_1/2_ = −1.02 V with a peak-to-peak separation (Δ*E*_*p*_) of 83 mV, assigned to Cu^2+^/Cu^+^ couple (Figure 3A). This assignment corroborates with ^1^H NMR studies using the chemical reductant cobaltocene, Co­(Cp)_2_ in CD_3_CN (Figure S10). However, the second redox event revealed a small, irreversible redox peak at −2.14 V, ascribed to a ligand-centered reduction as shown in Figure 3A. This ligand-based behavior is supported by the presence of a comparable redox feature at −2.02 V observed in the zinc-containing analogue (Figure S11). In addition, the redox peak for the protonated ligand scaffold alone was observed at −2.22 V (Figure S11). Varying scan-rate experiments of complex **2** confirmed diffusion-controlled redox processes (Figure S12). Upon CO_2_ exposure, the first reduction wave shifted anodically and a more positive potential is required for the oxidation peak, as shown in Figure 3A (blue line) and Figure S13A. This indicates that the Cu­(I) state reacts with CO_2_ following the EC mechanism. The spectroelectrochemical UV–vis measurements at −1.58 V in the presence of CO_2_ revealed a new spectrum with a distinct isosbestic point, further supporting this assignment (Figure S14). Under more negative potentials, a significant current increase was observed, overlapping the second irreversible redox peak. Nevertheless, the addition of trifluoroethanol (TFE) led to a marginal enhancement in current (Figure 3C). After voltammetric measurements, unlike complex **1** ^**Me**^ and **1** ^*i* **Pr**^, the rinse test suggested that complex **2** was stable over the time frame of cyclic voltammetry (Figure S13B).

**Figure 3:**
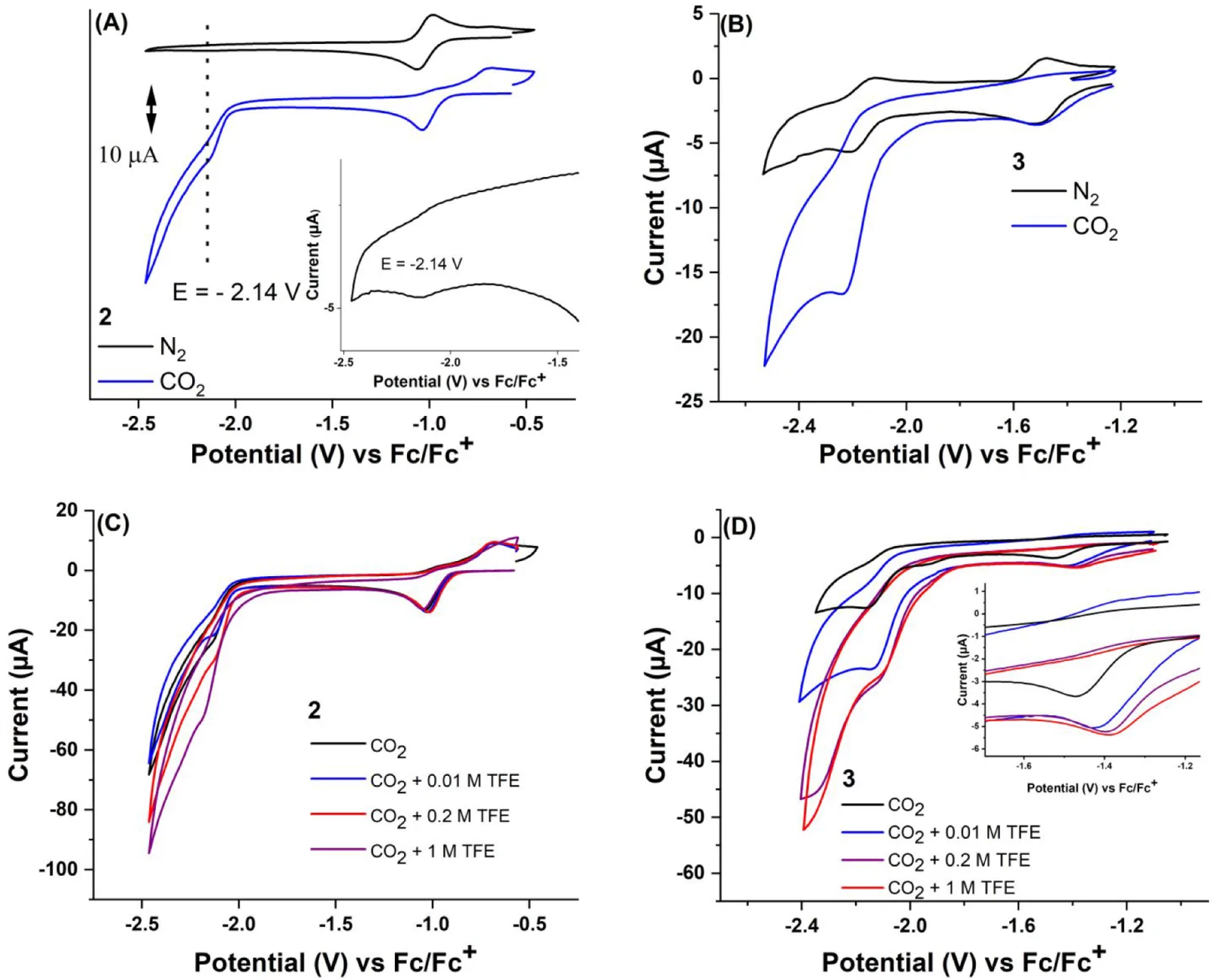
Cyclic voltammograms of: (A) complex **2** (1 mM in DMF) under N_2_ (black line) and in the presence of CO_2_ (blue line). The vertical dashed line refers to a ligand-based reduction at −2.14 V; (B) complex **3** (1 mM in DMF) under N_2_ (black line) and in the presence of CO_2_ (blue line); (C) complex **2** (1 mM in DMF) under CO_2_ with varying equivalents of TFE added as a proton source; and (D) complex **3** (1 mM in DMF) under CO_2_ with varying equivalents of TFE. Scan rate: 0.1 V s^–1^. The diameter of the working electrode is 3 mm.

### Electrocatalytic Properties of CO_2_ Reduction Mediated by Complex **3**

For the macrocyclic complex **3**, two redox couples were observed at *E*_1/2_ = −1.53 V and *E*_1/2_ = −2.19 V under N_2_, with Δ*E*_*p*_ of 68 and 84 mV, respectively (Figures 3B, S15). The redox behavior of **3** parallels that of the acyclic complexes **1** and **2**. The metal-centered reduction for **3** is shifted to a more negative potential, reflecting increased electron density at the copper center due to the dithioether coordination environment. More interestingly, the second ligand-based redox event was reversible. To elucidate the nature of these redox processes, UV-vis spectroelectrochemical experiments were conducted. The absorption peak near 530 nm, due to a d-d transition of the copper center, disappeared upon applying −1.6 V, with full recovery upon reversing the potential to +0.25 V (Figure S16).

In addition, the EPR signal corresponding to Cu­(II) vanished upon treatment with one equivalent of decamethylcobaltocene (Co­(Cp*)_2_, *E* ^0^ = −1.94 V) (Figure S17). Similar to complex **2**, ^1^H NMR studies with Co­(Cp*)_2_ in CD_3_CN revealed diamagnetic spectra of the one-electron reduced complex **3** (Figure S18). These observations confirm that the first redox event corresponds to the Cu­(II)/Cu­(I) redox couple. It is noteworthy that the ^1^H NMR spectrum of **1** ^*i* **Pr**^ after treatment with Co­(Cp)_2_ revealed only significantly broadened and diminished peaks in the aromatic region (Figure S19). This suggests the instability of the copper center within the purely N-donor pincer framework, consistent with the irreversible redox response observed in the cyclic voltammogram of **1** ^*i* **Pr**^ (Figure S4). To further probe the second redox feature, the cyclic voltammetry of a zinc-containing analogue, **3** ^**Zn**^ and the protonated macrocyclic ligand L3 were examined, revealing a redox event near −2.1 V (Figure S20). Interestingly, the protonated macrocyclic ligand L3 shows a quasi-reversible wave at −2.18 V, with improved electrochemical reversibility compared to related ligands L1^Me^, L1^*i*Pr^ (Figure S21), and L2 (Figure S11). Moreover, upon the reduction of ligand L3 and complex **3** with KC_8_ (*E*_1/2_ ∼ −2.44 V vs Fc^+/0^), an EPR signal at g = 2.002, characteristic of an organic ligand-centered radical (Figure S22) was observed. Finally, DFT calculations further support the idea that the second redox event involves the ligand center (Figure S38). Accordingly, the second redox event observed at −2.19 V in complex **3** is assigned to a ligand-based process.

Under a CO_2_-saturated atmosphere, we found that the first redox peak of complex **3** became irreversible (Figure 3B) and the electrocatalytic onset potential began around −2.1 V, as shown in Figure 3B. The introduction of additional equivalents of TFE resulted in a pronounced current enhancement (Figure 3D). To understand the nature of the first irreversible peak in the presence of CO_2_, spectroelectrochemical UV–vis experiments were conducted. As shown in Figure 4A, a new peak appeared at 625 nm when the electrode potential was applied at −1.61 V in the presence of CO_2_. This suggests CO_2_ binding to the reduced Cu­(I) center. Furthermore, upon addition of CO_2_ to the EPR-silent reduced complex **3** ^–^ (Figure S17), the resulting EPR spectrum of the CO_2_-treated complex **3** ^–^ exhibited a distinct feature compared to that of the parent complex **3**.

**Figure 4:**
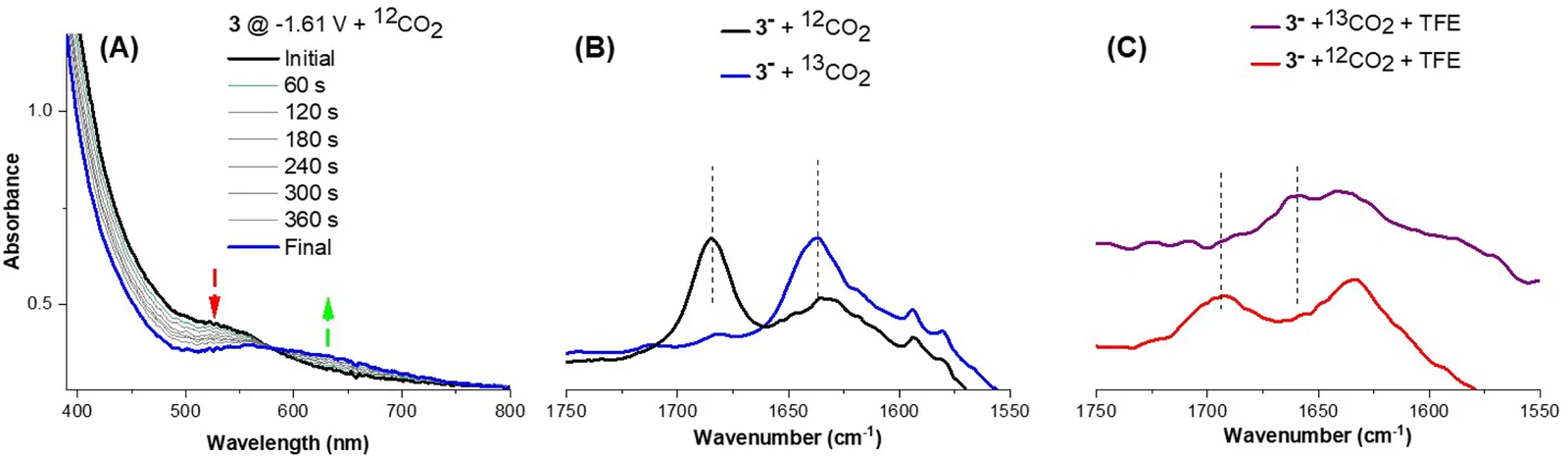
Spectroelectrochemical UV–vis spectra of complex **3**: (A) recorded under a CO_2_ atmosphere in DMF at −1.61 V. (B) Infrared spectra of reduced complex **3** ^–^ generated using 1.2 equiv Co­(Cp*)_2_ in acetonitrile under natural-abundance ^12^CO_2_ (black) and ^13^CO_2_ (99%) (blue). (C) Infrared spectra of **3** ^–^ under ^12^CO_2_ (red) with 10 equiv of TFE, and **3** ^–^ under ^13^CO_2_ (purple) with 10 equiv of TFE in acetonitrile.

Finally, infrared spectroscopy provided unequivocal evidence for the formation of this CO_2_-bound Cu­(II) intermediate species. Upon the introduction of CO_2_ to the reduced Cu­(I) species, two new peaks appeared at 1684 and 1633 cm^–1^ (Figures 4B and S23). When adding ^13^CO_2_ to the system, the 1684 cm^–1^ peak shifted to 1639 cm^–1^ (Δ*ν* = 45 cm^–1^), leading to overlap with 1633 cm^–1^ feature. Moreover, upon the addition of CO_2_ to a pre-mixed solution of complex **3** with one equivalent of Co­(Cp*)_2_ and 10 equiv of TFE, the IR peak shifted from 1684 to 1698 cm^–1^ as shown in Figure 4C. Additional preparations of the corresponding ^13^CO_2_-derived intermediate resulted in an additional shift to 1659 cm^–1^. These isotope-dependent shifts support the formation of a CO_2_-bound intermediate, CO_2_-**3** ^–^, followed by protonation to generate an HCO_2_-**3** species during electrocatalysis in the presence of acid. More importantly, the **3** ^–^, CO_2_-**3** ^–^ and HCO_2_-**3** intermediates were independently observed under electrochemical conditions by *in situ* spectroelectrochemical IR measurements, supporting their relevance in the electrocatalytic cycle (Figure S24).

Collectively, these results indicate that the first irreversible peak observed for complex **3** from the blue voltammogram in Figure 3B follows an EC mechanism in which the Cu­(I) state is first generated by electron transfer, followed by the chemical reaction of CO_2_ to form a Cu­(II)-CO_2_ adduct. To further evaluate the kinetics of this chemical step for complex **3**, scan rate-dependent cyclic voltammetry experiments were performed. The analysis was carried out using eq 1, which describes the relationship between the peak potential (*E_p_*) and the scan rate (ν) for an irreversible EC process.^41,42^ A linear dependence of *E*_*P*_ on ln­(1/ν) (Figure S25) yielded an observed rate constant, *k*_*obs*_ of 28.55 s^–1^. The second-order rate constant for CO_2_ binding was calculated to be 124.13 M^–1^s^–1^ considering the solubility of CO_2_ in DMF, 0.23 M.^43^

1 $$E_{p}=E^{0}-0.78\frac{RT}{F}+\frac{RT}{2F}\ln\left(\frac{RT}{F}\times \frac{k_{obs}}{\nu }\right)$$

*R*: ideal gas constant; *T*: temperature (K); *F*: Faraday constant; υ: scan rate (V/s); and *E* ^0^ = −1.53 V.

### Bulk Electrolysis and Turnover Frequency of Complexes **2** and **3**

Subsequently, bulk electrolysis experiments were conducted to verify the products of the electrocatalytic reduction of CO_2_ mediated by **2** and **3**. Bulk electrolysis was carried out in a CO_2_-saturated DMF solution in an H-cell for 20 min using 0.25 M TFE. The headspace product was analyzed by gas chromatography, and the liquid product was investigated by NMR. Bulk electrolysis of complex **2** at −2.37 V yielded a total Faradaic efficiency (FE) of 25% for both H_2_ and CO (Figure S26 and Table S2). Notably, postelectrolysis examination revealed the formation of a shiny reddish film on the surface of the glassy carbon electrode (Figure S27B). A further bulk electrolysis experiment in a fresh CO_2_-saturated DMF solution using the postelectrolysis electrode (in the absence of complex **2**) generated similar current densities and FE distributions for the products (Figure S26). This result indicates that heterogeneous species are involved in electrocatalysis. X-ray photoelectron spectroscopy (XPS) analysis of the postelectrolysis electrode surface revealed the formation of metallic copper (Figure S28). These results indicate that complex **2** underwent decomposition under reducing conditions to generate heterogeneous copper as the active electrocatalyst for CO_2_ reduction.

In comparison, complex **3** maintained stable current profiles across a range of applied potentials. The FE for CO production (85.2 ± 2.7%) and minimal H_2_ evolution (7.4 ± 0.4%) were observed at −2.37 V (Figure S29, Table S2). No formate was observed in any of the electrolysis solutions by NMR. To confirm the molecular integrity of catalyst **3** and exclude copper deposition as the origin of electrocatalytic activity, a combination of dynamic light scattering (DLS), UV–vis spectroscopy, XPS, and rinse tests was conducted (Figures S29–S31) after 20 min of electrolysis. Neither CO nor H_2_ products detected when the post-electrolysis glassy carbon electrode was reused in fresh CO_2_-saturated DMF, confirming the absence of active surface-bound species engaging in electrocatalysis. The DLS measurements showed no significant changes in nanoparticle size distribution before and after electrolysis, indicating no colloidal Cu particle formation. Finally, XPS revealed no Cu species on the electrode surface. However, when the electrode after 40 min of electrolysis was further analyzed by XPS, it revealed the presence of heterogeneous Cu species on the electrode surface (Figure S32). The post-electrolysis electrode in fresh CO_2_-saturated DMF began to produce a small amount of CO (7%) and H_2_ (9%), indicating the formation of surface-bound copper species at extended electrolysis times (Figure S33). Collectively, these findings establish the dithioether macrocyclic Cu­(II) complex **3** as a selective homogeneous electrocatalyst for CO_2_ reduction, exhibiting high Faradaic efficiency for CO production and improved stability compared with complexes **1** and **2**.

The turnover frequency (TOF) of CO_2_ reduction mediated by complex **3** was further estimated using high-speed scan voltammetry in the presence of TFE and CO_2_ as shown in Figure 5. As the scan rate increased, the voltammogram showed an S-shaped feature, and the limiting current became independent of scan rate at approximately 70 V s^–1^ (Figure S34). This behavior indicates a steady-state kinetic region, allowing for the determination of the apparent first-order rate constant, *k*_*obs*_ (i.e., TOF), using eq 2 ^44–46^ The resulting TOF was determined to be 2139 s^–1^. The limiting current exhibited no dependence on proton concentration, suggesting that proton transfer is not involved in the rate-determining step.

2 $$\frac{i_{lim}}{i_{p}}=2.24n\sqrt{\frac{RTk_{obs}}{F\upsilon }}$$

where *i*_*lim*_ is the limiting current in the presence of substrate and *i*_*p*_ is the peak current in the absence of substrate.

**Figure 5:**
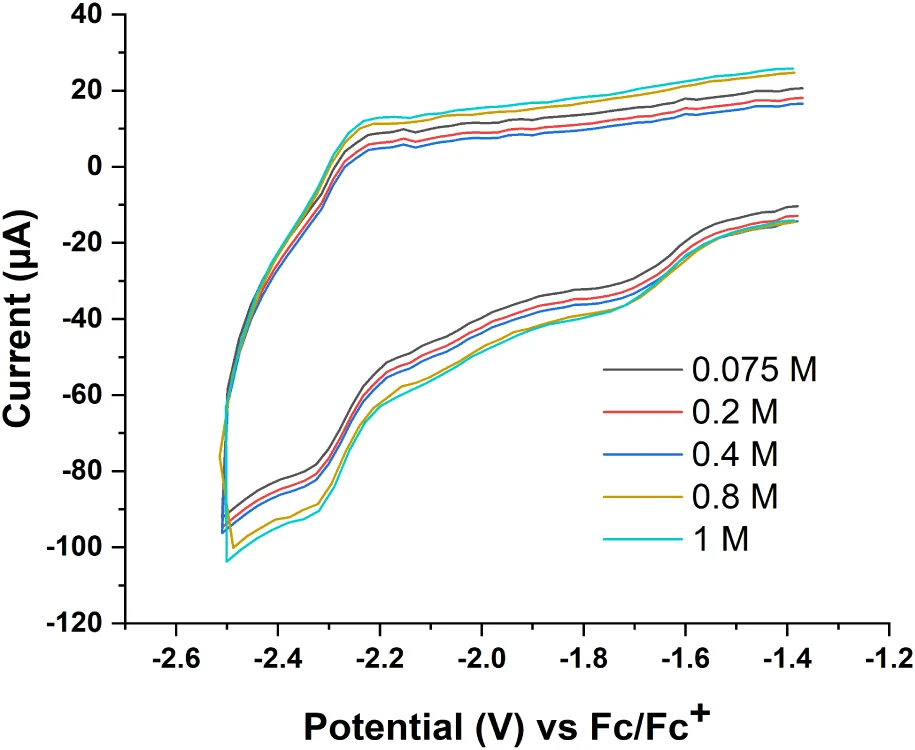
Cyclic voltammograms of complex **3** (1 mM) were recorded at 70 V s^–1^ in the CO_2_-saturated DMF using 0.1 M TBAPF_6_ as the supporting electrolyte, and varying concentrations of TFE were added. The diameter of the working electrode is 1 mm.

The improved stability and selectivity in CO_2_ reduction by complex **3** warrant further mechanistic investigations to uncover the underlying principles governing its electrocatalytic behavior.

### Mechanistic Investigations of CO_2_ Reduction Mediated by Complex **3**

As shown earlier, CO_2_ reduction mediated by complex **3** proceeds with an EC-type mechanism, initiated by electrochemical reduction of Cu­(II) to Cu­(I) at −1.53 V, followed by chemical binding of CO_2_. Chemical reduction of **3** with 1.5 equiv of Co­(Cp*)_2_ in CD_3_CN generated **3** ^–^, which exhibited two distinct resonances at 3.21 and 3.1 ppm in the ^1^H NMR spectrum, assigned to the methylene protons of the −S–CH_2_–CH_2_–S– motif (Figure S18). These signals indicate a structural reorganization favoring a lower coordination geometry, likely involving the dissociation of one thioether arm.

Upon exposure of **3** ^–^ to CO_2_, the sample reverted to a paramagnetic state, as evidenced by signal broadening in the ^1^H NMR (Figure S18) and the emergence of a new Cu­(II)-based EPR signal (Figure S17),^47,48^ supporting the reactivity of CO_2_ toward the Cu­(I) center. The IR measurements provided direct evidence of the formation of CO_2_-bound intermediates (Figures 4B, S23 and S24), **CO**_**2**_ **-3** ^–^. Following the addition of TFE, as shown in Figure 3D the voltammogram revealed a progressive anodic shift of the Cu^II/I^ redox couple. This feature indicates that protonation precedes the second electron transfer, suggesting that protonation likely occurs at the carboxylate moiety, forming **HCO**_**2**_ **-3**. Consistent with this assignment, IR spectroscopic measurements of separate electrochemically and chemically prepared samples support the formation of the **HCO**_**2**_ **-3** intermediate (Figure 4C, S24).

For the second redox event, as shown earlier, the second electron-transfer step involves ligand redox behavior (Figures S20, S22). Furthermore, the proton-concentration-independent behavior of the limiting current (Figure 5) suggests that the second electron transfer at the ligand site is gated to control the subsequent chemical step. Therefore, this ligand-centered redox event promotes C–O bond cleavage with **HCO_2_-3** through a proton-coupled electron-transfer pathway, yielding the final product, CO. To this end, the proposed mechanism for CO_2_ reduction catalyzed by complex **3** is summarized in Figure 6.

**Figure 6:**
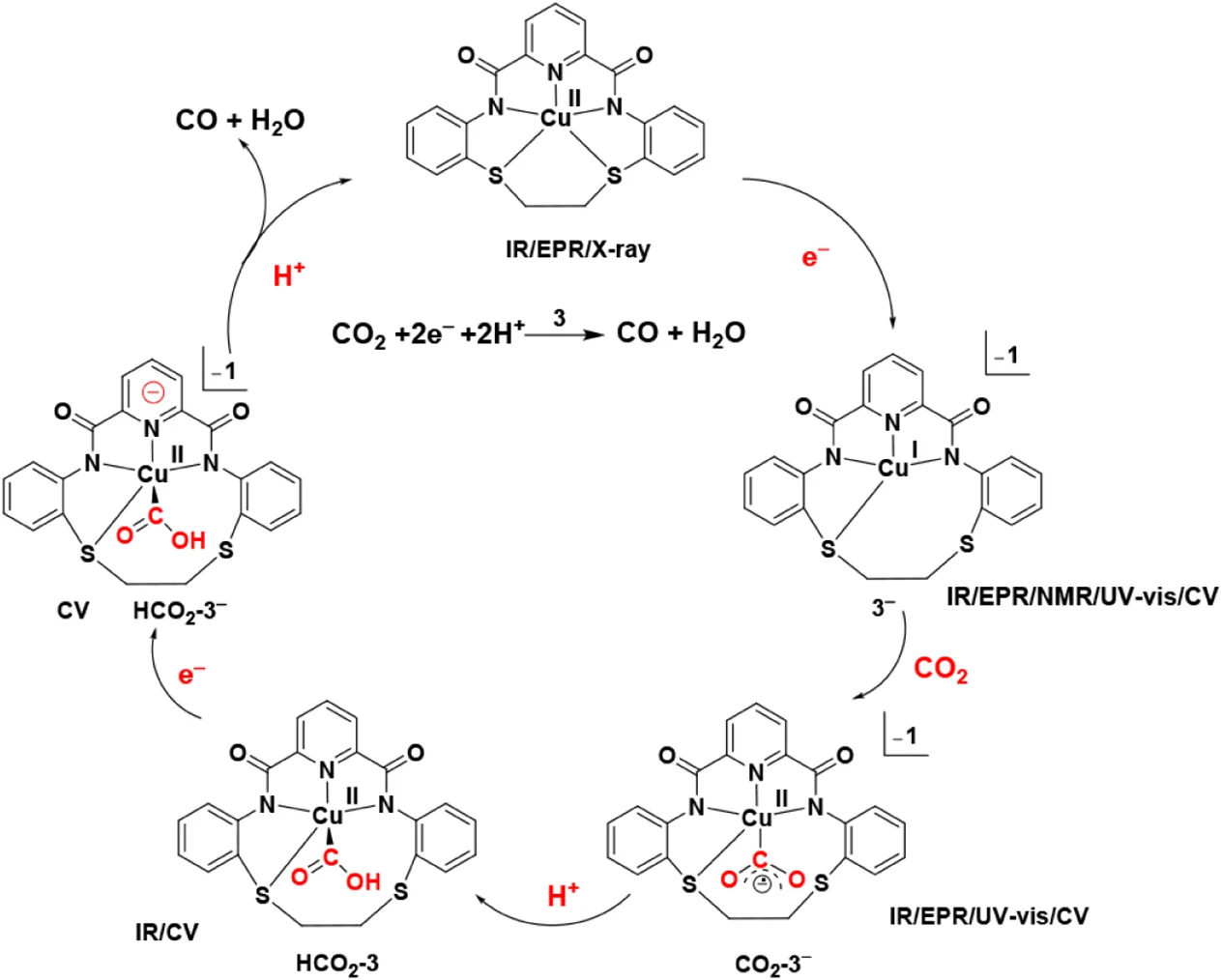
Proposed catalytic cycle and mechanism of CO_2_ reduction mediated by complex **3**.

### Role of Thioether Coordination and Ligand Rigidity in Complex Stability

Finally, the relationships among the primary coordination sphere, ligand rigidity, and complex stability were examined. In contrast to the reversible Cu­(II/I) redox couples observed for **2** and **3**, the thioether-free complexes **1** ^**Me**^ and **1** ^*i* **Pr**^ exhibited irreversible electrochemical behavior (Figures S3 and S4), indicating decreased stability upon reduction. Further, one-electron chemical reduction of the thioether-containing complexes afforded well-defined diamagnetic ^1^H NMR spectra, whereas reduced **1** ^*i* **Pr**^ only showed diminished signals, particularly in the aromatic region (Figure S19). These results demonstrate that incorporation of thioether donors enhances the stability of the reduced copper center on the basis of the HSAB principle.^22,23^

To probe the influence of different thioether-containing ligand frameworks, the reduced species **2** ^–^ and **3** ^–^ were prepared and reacted with CO_2_, followed by IR monitoring over a 10-min period. Exposure of **2** ^–^ to CO_2_ produced a new band at 1684 cm^–1^ assigned to a CO_2_-bound intermediate (Figure S35C). Upon treatment with ^13^CO_2_, this peak disappeared, red-shifting and merging with the ligand peaks (Figure S36). This isotopic shift confirms the formation of a CO_2_-bound adduct and demonstrates that, analogous to **3** ^–^, **2** ^–^ is also capable of CO_2_ binding. However, unlike the spectroscopically stable **CO**_**2**_ **-3** ^–^ intermediate (Figure S37), the IR spectrum of **CO**_**2**_ **-2** ^–^ gradually evolves over the same period (Figure S35C). Notably, spectral features directly collected from spectroelectrochemical IR measurements of **2** in the presence of CO_2_ were similar only to those of the chemically generated intermediates collected at 10 min rather than to the initial features (Figure S35C, S35D). Collectively, these results indicate that, unlike the stable **CO**_**2**_ **-3** ^–^ intermediate species, the CO_2_-bound intermediate derived from **2** undergoes subsequent transformation and may lead to instability. Notably, precipitation was observed occasionally when reacting the reduced complex **2** with CO_2_ during the preparation process, suggesting the susceptibility of **CO**_**2**_ **-2** ^–^.

To explore the nature differing stabilities of **CO**_**2**_ **-2** ^–^and **CO**_**2**_ **-3** ^–^, the DFT-optimized structures of **CO**_**2**_ **-2** ^–^ and **CO**_**2**_ **-3** ^–^ were analyzed using energy decomposition analysis coupled with natural orbitals for chemical valence (EDA-NOCV)^49,50^ to evaluate the nature of orbital contributions between the Cu center and the CO_2_ bond (Figure S39). EDA-NOCV analysis revealed that the orbital interaction in both CO_2_-bound intermediates is dominated by the first deformation-density channel, Δρ_(1)_ (Table S4). This dominant contribution arises primarily from pincer orbitals rather than thioether S donor orbitals. The corresponding orbital stabilization energy is greater for **CO**_**2**_ **-3** ^**–**^ than for **CO**_**2**_ **-2** ^–^ (Δ*E*_orb_(1) = −46.90 and −38.13 kcal/mol, respectively). Overall, these results suggest that the enhanced stability of **CO**_**2**_ **-3** ^–^ is attributed primarily to a rigid conformation that strengthens N3 pincer moiety–Cu orbital interactions within the macrocyclic framework.

## Discussion

These findings underscore the cooperative interplay among the primary coordination sphere of a copper center, the redox-active ligand framework, and ligand rigidity, which can collectively affect the stable and efficient electroreduction of CO_2_. Compared to the solely N-coordinated pincer ligands in complex **1** ^**Me**^ and **1** ^***iPr***^, the incorporation of thioether donors greatly increased the stability of copper­(I), as demonstrated by complexes **2** and **3**, thereby enhancing the reactivity toward CO_2_. The macrocyclic ligand design further enhances redox and catalytic stability, as well as performance. Unexpectedly, such an enhancement is more pronounced for the second reduction event centered on the ligand compared to complex **2**. These results indicate that the macrocyclic ligand framework not only provides a rigid coordination environment for overall complex stability but also minimizes structural reorganization to support redox reversibility. This structural rigidity can enhance electronic communication between the metal center and the redox-active ligand to prevent the overreduction of the copper center. In other words, the redox-active ligand serves as an intramolecular electron reservoir, facilitating charge transfer to the copper center during catalysis.

This strategy parallels the role of Fe–S cubane clusters in carbon monoxide dehydrogenase (CODH), where a redox-active unit stores electrons adjacent to the Ni catalytic site to enable multielectron transformations.^51,52^ Similarly, several molecular electrocatalysts for CO_2_ reduction have been proposed to exhibit metal–ligand redox cooperativity.^12,53,54^ Notably, the Chang group has highlighted how electronic coupling between the iron center and a redox-active ligand can modulate the reactivity of molecular CO_2_ reduction catalysts.^54^ These findings collectively underscore the essential role of the ligand framework and metal–ligand cooperativity in governing the activity and durability of copper-based electrocatalysts for CO_2_ reduction.

Several polypyridyl Cu complexes have been reported to degrade under electrocatalytic conditions, restricting their use primarily to photocatalytic applications.^17,35^ The ligand design strategy presented here offers a new direction for developing robust molecular systems capable of stabilizing electron-rich or low-valent first-row transition metals. Incorporation of thioether donors into the primary coordination sphere enhances the stability of low-valent Cu intermediates, as reflected in extended CV time profiles, while the macrocyclic framework further suppresses the demetalation pathways under prolonged electrolysis conditions. On the one hand, copper complexes have been employed directly as precursors to copper-complex-derived heterogeneous catalysts for CO_2_ reduction.^15,18,55–57^ On the other hand, the systematic modification of the ligand framework reveals distinct stability profiles across complexes, providing valuable design principles for the rational development of copper-complex-derived materials^8,58^ and for molecular surface modification of copper materials for heterogeneous CO_2_ electroreduction.^59^ In addition, the identification of the CO_2_-bound and protonated HCO_2_-bound intermediates provides direct molecular-level insight into CO_2_ activation at a copper center.

Finally, an electrocatalytic Tafel plot was constructed to benchmark the performance of complex **3** against those of other representative first-row transition-metal complexes. As shown in Figure 7, the electrocatalytic activity of complex **3** is comparable to that of most reported molecular complexes in terms of TOF. The relatively high overpotential can be attributed to the dianionic nature of the ligand framework. This limitation could potentially be mitigated by incorporating positively charged groups or proton relay functionalities in the secondary coordination sphere,^60^ as exemplified by Fe-TDHP and Fe–O–TMA, which outperform FeTPP despite the similarly dianionic porphyrin framework (Figure S40).^61–65^ Such modifications to derivatives of complex **3** are currently underway.

**Figure 7:**
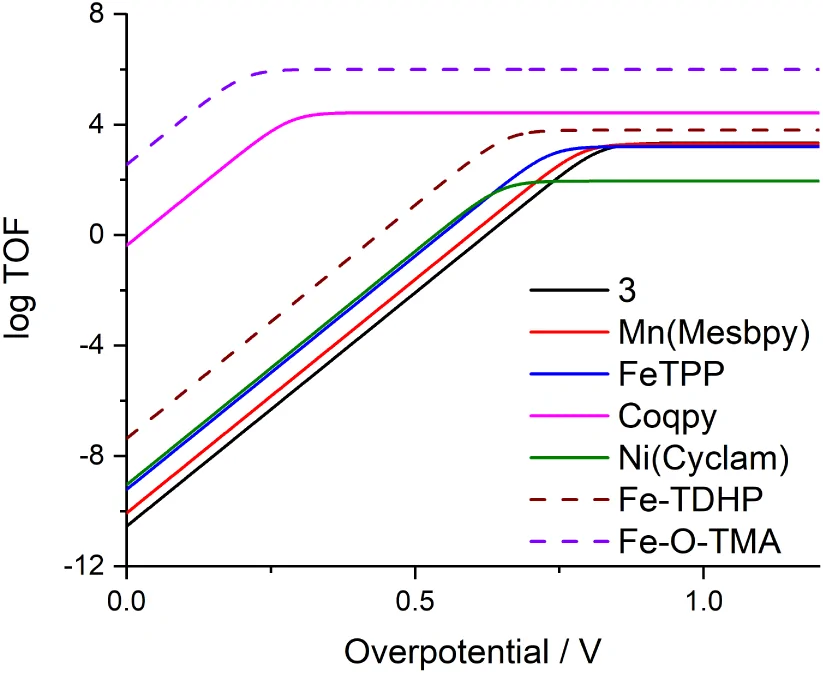
Catalytic Tafel plot of first-row transition-metal complexes for the reduction of CO_2_ to CO. The corresponding molecular structures and information on the complexes can be found in SI.

## Conclusion

Through systematic ligand design, we demonstrate that the integration of soft–hard donor interactions and a rigid redox-active macrocyclic scaffold not only stabilizes reactive intermediates but also promotes efficient electron delivery during turnover. These findings demonstrate that rational ligand design, balancing the coordination environment and donor identity, offers a pivotal strategy for advancing copper-based molecular electrocatalysts. The design principles established here may also serve as a foundation for developing complex-derived copper materials for heterogeneous CO_2_ reduction applications.

## Supplementary Material



## Notes

Materials and methods; experimental procedures; additional spectroscopic and electrochemical results for ligands and complexes; DFT calculation results; and crystallographic details (PDF)
