# Supplementary material for: Systematic Ligand Modification Tunes the Stability and Reactivity of Copper Complexes for the Electrocatalytic Reduction of Carbon Dioxide
Source: J Am Chem Soc. 2026 Sep 8;148(37):39936–45. doi: 10.1021/jacs.6c11205 (PMC13614178; doi:10.1021/jacs.6c11205)
Supplement: Supplementary file 1 [file ja6c11205_si_001.pdf]

Supporting Information

Systematic Ligand Modification Tunes the Stability and Reactivity of Copper Complexes for Electrocatalytic Reduction of Carbon Dioxide

Ramamoorthy Ramasubramanian[a], Charasee Laddika Dayawansa[a], Jyun-Chi Lee[a], Rui-Ze Xu[a], Jiun-Shian Shen[b], Tzu-Hsien Tseng[c], Tsz-Fai Leung[a], Tiow-Gan Ong[d], Glenn P. A. Yap[e] and Vincent C.-C. Wang[a,f*]

vincentwang64@yahoo.com or vincent.wang@mail.nsysu.edu.tw

[a]Department of Chemistry, National Sun Yat-sen University, Kaohsiung 80424, Taiwan, R.O.C.

[b]Department of Chemistry, National Taiwan University, Taipei 10617, Taiwan, R.O.C.

[c]Instrument Center, National Chung Hsing University, Taichung, 402202, Taiwan, R.O.C.

[d]The School of Science and Engineering, The Chinese University of Hong Kong, Shenzhen, Longgong, Guangdong, China

[e]Department of Chemistry and Biochemistry, University of Delaware, Newark, Delaware 19716, United States

[f]Green Hydrogen Research Center, National Sun Yat-sen University, Kaohsiung 80424, Taiwan, R.O.C.

Table of Contents

General Procedures and Chemicals 2

Experimental characterizations and measurements 7

Computational Calculations and Analysis 10

Supporting Results/Figures 12

Supporting Tables 49

Characterization Data of Ligands and Complexes 50

Coordinates of optimized structure 61

References 72

General Procedures and Chemicals

Nitrogen (N_2, 99.99%) and carbon dioxide (CO_2, 99.999%) were used as received. $^{13}CO_2$ (99.0 atom %, <3 atom % ^{18}O) was purchased from Sigma Aldrich. Dichloromethane (DCM, Macron, 99.8%) was also used without further purification. Acetonitrile (J.T. Baker, >99.9%), N, N-dimethylformamide (DMF, Macron, ACS reagent grade), and diethyl ether (Echo, 95%) were dried over 3 Å molecular sieves overnight prior to purification using solvent purification systems (Vigor Gas Purification Technologies or Pure Process Technology). The electrolyte tetrabutylammonium hexafluorophosphate ($TBAPF_6$, NOVA Materials, 99%) was recrystallized before use. Solvents were degassed by at least five freeze–pump–thaw cycles before transfer into a nitrogen-filled glovebox (Vigor; H_2O < 0.1 ppm, O_2 < 0.5 ppm) and subsequently stored over 3 Å molecular sieves. Potassium graphite (KC_8) was prepared according to standard literature procedures.[1]

The following chemicals were purchased and used as specified: 2,6-pyridinedicarboxylic acid (NOVA Materials), 1,2-dibromoethane (NOVA Materials), 2,6-dimethylaniline (NOVA Materials), 2-(methylthio)aniline (NOVA Materials), potassium carbonate (NOVA Materials), thionyl chloride (NOVA Materials), 2-aminothiophenol (Thermo Scientific Chemicals, 98%); copper(II) trifluoromethanesulfonate (Alfa Aesar, 99%), cobaltocene (Sigma-Aldrich) and decamethylcobaltocene (Sigma-Aldrich). Unless otherwise specified, all chemicals and solvents were used as received without further purification.

Synthetic Procedures

Synthesis of L2

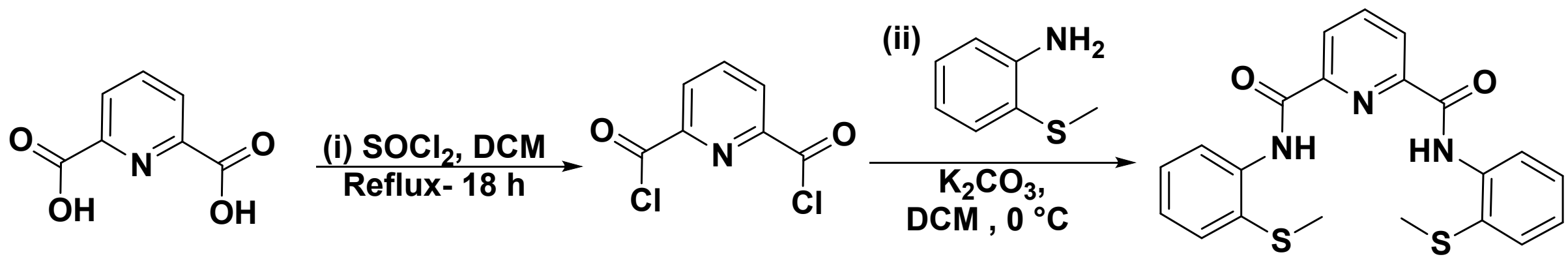

L2

In a 100 mL two-neck round-bottom flask equipped with a magnetic stir bar, 2,6-pyridinedicarboxylic acid (1.20 g, 0.0072 mol) was dissolved in dry dichloromethane (15 mL). To increase solubility, a drop of N,N-dimethylformamide (DMF) was added. The resulting solution was stirred at room temperature for 15 min, after which thionyl chloride (2.5 mL, 0.035 mol, 5 equiv) was added dropwise. The reaction mixture was heated to reflux (52 °C) under a nitrogen atmosphere for 18 h. During this time, the solution gradually changed color from colorless to orange, consistent with the *in situ* formation of pyridine-2,6-dicarbonyl dichloride. The solvent and excess thionyl chloride were removed under reduced pressure to afford the crude acid chloride, which was used directly in subsequent reactions without further purification.

Separately, 2-(methylthio)aniline (2.10 g, 15.0 mmol, 2.1 equiv) and potassium carbonate (3.0 g) were dissolved in DCM (10 mL) with two drops of DMF. The solution was cooled to 0 °C in an ice bath. The freshly prepared pyridine-2,6-dicarbonyl dichloride solution was added

dropwise to the cooled 2-(methylthio)aniline solution. The reaction was maintained at 0 °C for 3 hours, then gently heated to 40 °C for 1 hour, and finally stirred overnight at room temperature to ensure complete conversion. The mixture was quenched by a slow addition of saturated aqueous sodium chloride, and the organic layer was separated. The aqueous phase was extracted with DCM (15 mL) two times, and combined organic layers were dried over anhydrous Na_2SO_4, filtered, and concentrated under reduced pressure. The resulting crude solid was purified by trituration with diethyl ether (10 mL) three times to afford **L2** as a white crystalline solid in 80% yield (1.85 g).

^{1}H NMR (300 MHz, $CDCl_3$) δ in ppm, 10.59 (s, 2H, N_fH), δ8.54(d, $^3J_{H-H}$=9 Hz, 1H, C_eH), δ8.36(d, $^3J_{H-H}$=9 Hz, 1H, C_bH), δ8.17(t, $^3J_{H-H}$=18 Hz, 1H, C_gH), δ7.51(d, $^3J_{H-H}$=6 Hz, 1H, C_hH), δ7.37(t, $^3J_{H-H}$=15 Hz, 1H, C_dH), δ7.18(t, $^3J_{H-H}$=15 Hz, 1H, C_cH), δ2.40(s, 6H, C_a) ppm. ^{13}C NMR (75MHz, $CDCl_3$) δ161.7(C_b), 149.5(C_c), 139.9(C_d), 137.3(C_e), 131.9 (C_f), 128.5(C_j), 126.0(C_h), 125.7(C_g), 122.2(C_i), 18.73(C_a)ppm. HR-ESI-MS (m/z): Calculated: 410.0997; Found: 410.0996.

Synthesis of Ligand L3

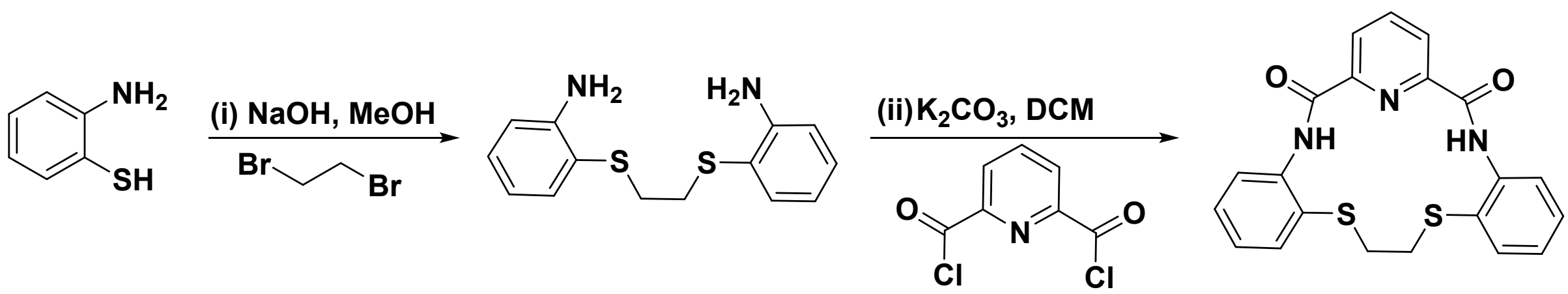

L3

1,2-Dibromoethane (0.6 mL, 6.9 mmol) was added dropwise to a refluxing solution of 2-aminothiophenol (1.5 mL, 14 mmol) and sodium hydroxide (0.6 g, 14 mmol) in methanol (100 mL). The mixture was stirred under reflux for 16 hours. After completion, the solvent was removed under reduced pressure, and the residue was cooled to room temperature. Water (50 mL) was added, and the mixture was extracted with diethyl ether (4 × 50 mL). The combined organic extracts were dried and concentrated to yield 1,2-bis(aminophenylthio)ethane (1.3 g, 68% yield) as a solid.

In the next step, 1,2-bis(aminophenylthio)ethane (0.80 g, 2.9 mmol) was dissolved in DCM, and potassium carbonate (3.0 g, excess) was added. The reaction mixture was stirred at 0 °C for 30 minutes. A freshly prepared solution of pyridine-2,6-dicarbonyl dichloride (2.8 mmol) in DCM was then added dropwise while maintaining the temperature at 0 °C. The mixture was stirred for 3 hours at 0 °C, followed by heating at 40 °C for 1 hour, and then allowed to stir overnight at room temperature. During the course of the reaction, the solution color changed from greenish-yellow to a white dispersion. The mixture was quenched by a slow addition of saturated aqueous sodium chloride, and the organic layer was separated. The aqueous phase was extracted with DCM (2 × 15 mL), and combined organic layers were dried over anhydrous Na_2SO_4, filtered, and concentrated under reduced pressure. The resulting crude solid was purified by trituration with diethyl ether (10 mL) three times to afford **L3** as a pale solid (0.9 g, 79% yield).

^1H NMR (300 MHz, $CDCl_3$), δ in ppm, 11.65 (s, 2H), 8.54 (d, $^3J_{H-H}$=9 Hz, 2H), 8.47 (d, $^3J_{H-H}$=6 Hz 2H), 8.21 (t, $^3J_{H-H}$=15 Hz, 1H), 7.54 (d, $^3J_{H-H}$=6 Hz 2H), 7.40 (t, $^3J_{H-H}$=15 Hz, 2H), 7.15 (t, $^3J_{H-H}$=15 Hz 2H), and 3.20 (s, 4H). ^{13}C NMR ($CDCl_3$), 160.80, 148.72, 140.15, 138.98, 129.39, 124.96, 124.90, 123.04, 120.54, 34.88 ppm. HR-ESI-MS (m/z): Calculated: 406.0684; Found: 406.0684.

Synthesis of Ligands L1^{Me} and L1^{iPr}

Ligands L1^{Me} and L1^{iPr} were synthesized using a modified literature procedure.[2, 3] In brief, a freshly prepared solution of pyridine-2,6-dicarbonyl chloride (1.02 g, 5.00 mmol) in DCM (15 mL) was added dropwise at 0 °C to a stirred mixture containing the respective aniline derivative, 2,6-dimethylaniline (1.21 g, 10 mmol) for **L1^{Me}** or 2,6-diisopropylaniline (1.77 g, 10 mmol) for **L1^{iPr}** , along with excess potassium carbonate (3.0 g) in a DCM (15 mL) were added two drops of DMF as a co-solvent to aid dissolution. The reaction was maintained at 0 °C for 30 minutes and then allowed to warm to room temperature, followed by stirring overnight.

Upon completion of the reaction, it was quenched by the slow addition of ice-cold water at 0 °C. The organic layer was separated and extracted with DCM (15 mL) two times, dried over anhydrous sodium sulfate, and concentrated under reduced pressure. The crude product was purified to afford the target ligands. **L1^{Me}** and **L1^{iPr}** were obtained as white solids in yields of 85% (1.58 g) and 77% (1.87 g), respectively.

^1H NMR (300 MHz, $CDCl_3$), **L1^{Me}**, δ in ppm, 9.17 (s, 2H), 8.51(d, $^3J_{H-H}$=6 Hz, 2H), 8.14 (t, $^3J_{H-H}$=9 Hz, 1H), 7.13 (m, 6H), 2.27 (s, 12H) and ^{13}C NMR ($CDCl_3$), 161.45, 148.90, 139.54, 135.26, 133.25, 128.38, 127.59, 125.75, 18.58 ppm; HR-ESI-MS (m/z): Calculated $[M +H]^+$; 374.1869 and found 374.1857 $[M+H]^+$. **L1^{iPr}** ^1H NMR (300 MHz, $CDCl_3$), δ in ppm, 9.02 (s, 2H), 8.55 (d, $^3J_{H-H}$=9 Hz, 2H), 8.22 (t, $^3J_{H-H}$=18 Hz, 1H), 7.36 (t, $^3J_{H-H}$=15 Hz 2H), 7.26 (d, $^3J_{H-H}$=9 Hz, 2H), 3.15 (s, $^3J_{H-H}$=6.8 Hz, 4H) and 1.22 (d, $^3J_{H-H}$=9 Hz, 24H). ^{13}C NMR ($CDCl_3$), 162.45, 147.90, 145.90, 139.54, 135.26, 133.25, 128.38, 127.59, 125.75, 29.97, 22.94 ppm; HR-ESI-MS (m/z): Calculated $\{[M +H]^+\}$: 486.3121 and found 486.3100 $[M+H]^+$.

Synthesis of copper(II) complexes

Synthesis of [Cu^{II} (L2)-MeOH], 2

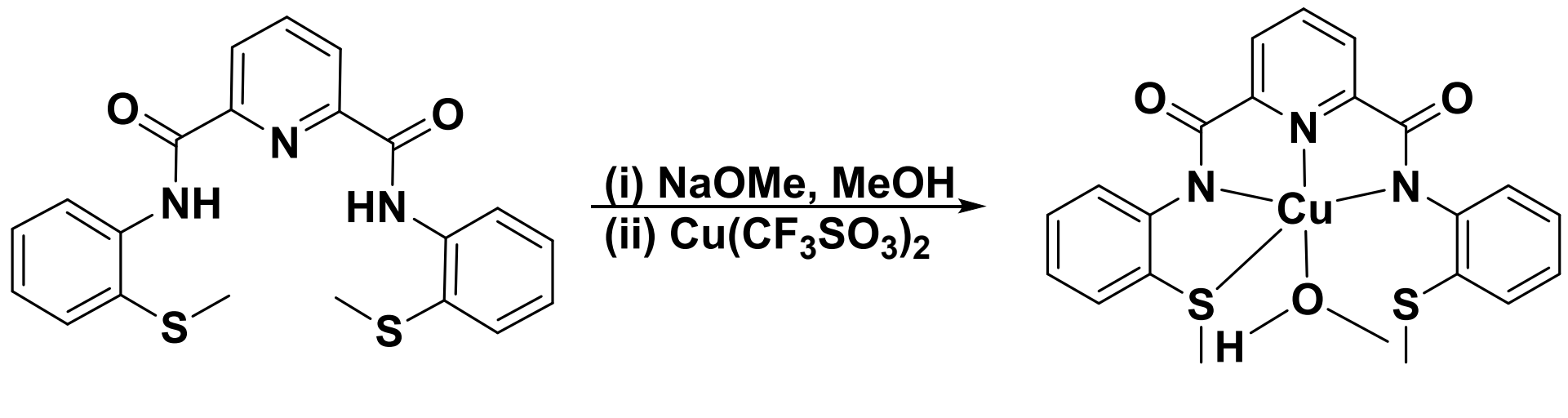

L2 **2**

A mixture of ligand L2 (173 mg, 0.424 mmol) and copper(II) trifluoromethanesulfonate ($Cu(CF_3SO_3)_2$, 148 mg, 0.410 mmol) was placed in a two-neck round-bottom flask, evacuated, and backfilled with nitrogen three times. The solids were then dissolved in methanol (3 mL) under a nitrogen atmosphere and stirred for 5 minutes, during which the solution turned green. In a separate flask, sodium methoxide (NaOMe, 140 mg, 6 equivalents) was dissolved in methanol (7 mL) and added dropwise to the reaction mixture. The resulting solution was stirred under nitrogen overnight, during which the color gradually changed from green to brown.

The next day, the mixture was concentrated to dryness under reduced pressure. The residue was dissolved in acetonitrile (50 mL) and filtered to remove some solid impurities. The filtrate was concentrated again, and the resulting crude product was redissolved in dichloromethane (50 mL) and filtered to remove sodium triflate ($NaCF_3SO_3$), resulting in the formation of a brown solid as the final product with 59% yield. The X-ray crystals were obtained by slow diffusion of diethyl ether into a methanol solution of the complex.

Analytical Data: HR-ESI-MS (m/z): Calculated $[M - MeOH + H]^+$: 471.0136; found 471.01318. Elemental analysis: *Calculated for* $C_{22}H_{21}CuN_3O_3S_2$: C, 52.52; H, 4.21; N, 8.35. *Found*: C, 52.54; H, 4.02; N, 8.51. EPR (X-band, 77 K): $g_x = 1.98$, $g_y = 2.04$, and $g_z = 2.29$.

Synthesis of [Cu^{II}(L3)], 3

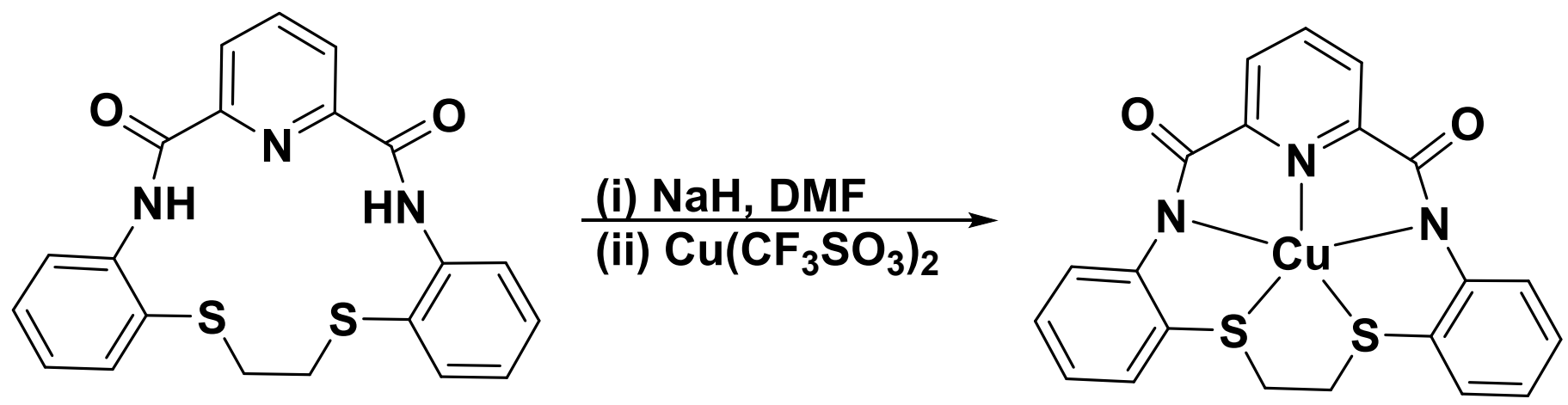

L3 **3**

Sodium hydride (NaH; 0.042 g, 1.8 mmol; 60% dispersion in mineral oil) was suspended in dry DMF (3 mL) and stirred for at least 10 mins under a nitrogen atmosphere. To this suspension, a solution of ligand L3 (0.122 g, 0.30 mmol) was added. Upon addition, mild gas evolution was observed, and the mixture developed a pale yellow suspension. The reaction was stirred for an additional 15 minutes at room temperature to ensure complete deprotonation of the ligand. A solution of $Cu(OTf)_2$ (0.100 g, 0.277 mmol) in DMF (4 mL) was then added

slowly to the reaction mixture, resulting in a gradual color change to dark black-brown solution. The reaction was stirred for overnight at room temperature, after which it was filtered to remove any insoluble material.

The crude product was dissolved in a DCM/ACN (9:1) mixture, filtered to remove $NaCF_3SO_3$, and the filtrate was concentrated under reduced pressure. Once again residue was redissolved in pure DCM, filtered, and evaporated to dryness. The resulting solid was washed twice with diethyl ether to afford a brown-black microcrystalline product. Single crystals suitable for X-ray diffraction were obtained via slow vapor diffusion of diethyl ether into a solution of a portion of the complex in ACN/THF/$CHCl_3$. Yield: 0.07 g (49%).

Analytical Data: HR-ESI-MS (m/z): Calculated $[M + H]^+$ 468.9980; Found: 468.9976. Elemental analysis: *Calculated for* $C_{21}H_{15}CuN_3O_2S_2$: C, 53.78; H, 3.22; N, 8.96; Found: C, 53.27; H, 2.97; N, 8.8. EPR (X-band, 77 K): $g_x = 1.95$, $g_y = 2.03$, $g_z = 2.23$.

Synthesis of Zinc complexes 2^{Zn} and 3^{Zn}

L2 ligand (0.244 mmol) was added to a two-neck flask, followed by NaH (36 mg, 1.5 mmol, 6 equiv). The flask was evacuated and backfilled with nitrogen three times to remove residual air. Anhydrous acetonitrile (3 mL) and one drop of DMF were added to enhance solubility, and the resulting suspension was stirred for 30 min. $Zn(ClO_4)_2$ (65 mg, 0.245 mmol, 1 equiv), dissolved separately in acetonitrile, was added to the reaction mixture, turning the solution from white to yellow. The reaction was allowed to proceed under a nitrogen atmosphere overnight. After completion, the solvent was removed under reduced pressure, and the crude product was washed with diethyl ether three times to give a yellow solid (73.9 mg, 64% yield).

For 3^{Zn}, the same procedure was followed except that anhydrous DMF was used as the sole solvent for the entire reaction instead of acetonitrile. $Zn(ClO_4)_2$ (65 mg, 0.245 mmol, 1 equiv) was dissolved in DMF and added to the ligand/NaH suspension in DMF, after which the solution turned yellow. The reaction mixture was stirred overnight under nitrogen, the solvent was removed under reduced pressure, and the crude product was washed with diethyl ether (3×) to yield a yellow solid (41 mg, 36% yield). The 3^{Zn} complex was found to be poorly soluble in most common solvents and was therefore used for further characterization without additional purification.

Analytical Data: HR-ESI-MS (m/z): Calculated 2^{Zn} $[M + H]^+$ 472.0132; Found: 472.0150; Calculated 3^{Zn} $[M + H]^+$ 469.9975; Found: 469.9987.

2^{Zn} - H NMR (300 MHz, CD_3OD), δ in ppm, 8.48 (d, 2H), 8.3 (t, 1H), 8.00 (m, 2H), 7.52 (d, 2H), 7.36 (m, 2H), 7.05 (t, 1H), 6.75 (m, 1H), 2.98 (s, 3H) and 2.86 (s, 3H). ^{13}C NMR (CD_3OD), 182.09, 170.9, 150.1, 141.44, 137.02, 131.24, 128.25, 127.42, 126.63, 124.48, 19.32, 17.51 ppm.

3^{Zn} - H NMR (300 MHz, CD_3OD), δ in ppm, 8.2 (2H), 8.03 (1H), 7.83 (1H), 7.34 (2H), 7. 21 (2H), 7.06 (1H), 6.71 (1H), 6.54 (1H), 2.99 (2H) and 2.86 (2H).

Synthesis of Complexes 1^{Me} and 1^{iPr}

Following a modified literature procedure,[2, 4] complex 1^{Me}-OH was synthesized as briefly described below. Ligand $L1^{Me}$ (223 mg, 0.60 mmol) and $Cu(CF_3SO_3)_2$ (216 mg, 0.60 mmol) were dissolved in DMF (10 mL) and stirred for 10 minutes. Tetraethylammonium hydroxide (Et_4NOH, 25% in methanol, 1.1 mL, 1.94 mmol) was then added dropwise, and the reaction mixture was stirred at room temperature for 12 hours, affording a dark blue solution. A small amount of insoluble material was removed by filtration. Evaporation of the filtrate to dryness yielded the hydroxide complex [$\mathbf{1}^{\mathbf{Me}}$–OH]Et_4N, analogous to Holm's Cu–OH system[2] and employed here for CO_2 fixation studies.

A portion of this complex was redissolved in DMF and layered with diethyl ether (60 mL), forming a cloudy suspension from which dark blue crystals of [$\mathbf{1}^{\mathbf{Me}}$–HCO_2]Et_4N gradually developed over 24 hours. The crystalline product was isolated by filtration and dried under reduced pressure to afford 72% in overall yield (0.23 g) which is further used for electrochemical studies. This complex was subsequently used for electrochemical investigations.

Complex $\mathbf{1}^{iPr}$ was synthesized analogously following a previously reported procedure, to afford the product in 67% yield (0.27 g).[3]

Analytical Data: [$\mathbf{1}^{\mathbf{Me}}$-OH]Et_4N: HR-ESI-MS (m/z): Calculated {[$\mathbf{1}^{\mathbf{Me}}$-OH]$^-$} 451.0957; Found: 451.0949 and [$\mathbf{1}^{\mathbf{Me}}$-HCO_2]Et_4N*:* HR-ESI-MS (m/z): Calculated [1^{Me}-HCO_2]$^-$ 479.0906; Found: 479.0900; $\mathbf{1}^{iPr}$*:* HR-ESI-MS (m/z): Calculated [M]$^-$ 563.2209; Found: 563.2227. Elemental analysis: *Calculated for* $C_{39}H_{58}CuN_4O_3$ C, 67.45; H, 8.42; N, 8.07; Found: C, 68.1; H, 8.80; N, 7.93.

Experimental characterizations and measurements

Electrochemical Measurements

Electrochemical studies were conducted using a Metrohm Autolab PGSTAT302N potentiostat equipped with an FRA32M module. A conventional three-electrode setup was employed, comprising a glassy carbon as a working electrode (3 mm diameter for standard experiments, 1 mm for high-scan rate measurements), a platinum wire as a counter electrode, and a silver wire as a quasi-reference electrode, all immersed in DMF. All potentials were referenced to the ferrocenium/ferrocene (Fc^+/Fc) redox couple, introduced as an internal standard after the measurements. For high-speed voltammetry, the solution resistance was determined via electrochemical impedance spectroscopy (EIS) prior to measurement. The measured uncompensated resistance (R_u) was used to apply positive feedback IR compensation, following established procedures.[5]

TOF calculation and Tafel plot analysis:

The Tafel plot is constructed by the following equation[6, 7]

$$TOF = \frac{TOF_{max}}{1 + \exp(f\Delta E)\exp(-f\eta)}$$

where

$f = \frac{F}{RT}$ F is the Faraday constant; R is the ideal gas constant. T is temperature in Kelvin. Here the value is 298 Kelvin degrees.

$\Delta E = E_{CO2/CO} - E_{cat/2}$

$E_{CO2/CO}$ is the formal reduction potential of CO_2 to CO in the solvent. The value in organic solvent (DMF or ACN) is followed by reference 6.

$E_{cat/2}$ is the potential value of a half-wave potential of the electrocatalytic S-shape voltammogram. This value is generally equal to the redox potential (i.e. $E_{1/2}$) of the metal center of the catalyst in the absence of substrate.[9]

H is overpotential against $E_{CO2/CO}$

The values used to construct the Tafel plot (Figure 7 in main texts)

**Catalyst**	**ΔE (V)**	**TOF_{max} (s^{-1})**	**Proton source(solvent)**	**References**
Mn(Mesbyp)	0.79	2.00×10^3	0.2 M TFE (ACN)	8
FeTPP	0.734	1.6×10^3	0.1 M Phenol (DMF)	9
Coqpy	0.248	3.3×10^4	3 M Phenol (ACN)	10
Ni(Cyclam)	0.65	90	25% Water (ACN)	11
Fe-TDHP	0.69	1.6×10^4	3 M Phenol (DMF)	7
Fe-O-TMA	0.204	10^6	3 M Phenol (DMF)	5
**3**	0.82	2.14×10^3	0.2 M TFE (DMF)	This work

Controlled-Potential Electrolysis (CPE)

Controlled potential electrolysis (CPE) experiments were performed in a two-compartment H-cell separated by a glass frit. Each compartment contained 7.5 mL of DMF with 0.1 M tetrabutylammonium hexafluorophosphate ($TBAPF_6$) as the supporting electrolyte. The copper complexes (0.5 mM) and trifluoroethanol (500 equiv, 250 mM) were added to both compartments. Prior to electrolysis, the solutions were saturated with CO_2 by purging for at least 30 min. Glassy carbon plates (1.5 × 1.0 cm^2) were used as the working and counter electrodes. A silver wire quasi-reference electrode was employed and positioned in a frit-isolated compartment to prevent direct contact with the bulk electrolysis solution and minimize potential drift arising from changes in electrolyte composition during electrolysis.

Gaseous products (H_2, CO, CH_4) were quantified using a Shimadzu Nexis GC-2030 gas chromatograph equipped with a Micropacked ST column (2.0 m × 1.0 mm ID) and a barrier discharge ionization detector. High-purity helium (99.9999%) was used as the carrier gas at a pressure of 300 kPa. For analysis, 300 µL of headspace gas was sampled using a gastight syringe (VICI). Liquid-phase products, including formate, were quantified after extraction from the post-electrolysis solution. All electrolysis experiments were performed in duplicate under identical conditions to ensure reproducibility.

Rinse Test for Homogeneity

To evaluate whether the electrolysis of CO_2 reduction is homogeneous, a rinse test was performed. After the completion of controlled-potential electrolysis, the glassy carbon working electrode was thoroughly rinsed with DMF to remove any physisorbed species, without mechanical polishing. The electrode was then transferred to a fresh electrochemical cell containing CO_2-saturated DMF with 0.1 M $TBAPF_6$ and trifluoroethanol, but no added copper complexes.

UV–Vis and Infrared Spectroscopy

UV–visible spectra were acquired using a Cary 5000 UV–Vis–NIR spectrophotometer (Agilent Technologies). IR spectra were recorded with a Nicolet Apex FT-IR (thermos scientific) spectrometer equipped with a MCT detector.

Spectroelectrochemical Measurements

Spectroelectrochemical UV-visible spectroscopy experiments were carried out in a quartz thin-layer cuvette (1 mm path length) equipped with a platinum gauze working electrode, a platinum wire counter electrode, and a silver wire quasi-reference electrode. UV–visible spectra were recorded using an Ocean Insight SR2 UV–vis spectrometer, with the applied potential controlled via a Metrohm Autolab PGSTAT302N potentiostat.

Spectroelectrochemical infrared measurements were conducted using an optically transparent thin-layer electrochemical (OTTLE) cell equipped with CaF_2 windows. A platinum mesh, platinum wire, and silver wire were used as the working, counter, and quasi-reference electrodes, respectively.

Electron Paramagnetic Resonance and X-ray Photoelectron Spectroscopy (XPS)

Electron paramagnetic resonance (EPR) measurements were performed on a Bruker EMXplus 10/12 spectrometer operating at X-band frequencies. Low-temperature measurements were conducted using a finger dewar filled with liquid nitrogen (77 K). EPR samples were prepared in a 1:1 mixture of DMF and toluene and frozen in liquid nitrogen. Spectral simulations were carried out using the EasySpin (v6.0.6) software package.[12]

XPS measurements were performed using a ULVAC-PHI PHI 5000 VersaProbe instrument. Following electrolysis, electrode surfaces were rinsed with fresh DMF, dried under nitrogen, and subjected to analysis.

X-ray Crystallography

X-ray structural analysis for **2** and **3**: Crystal data and refinement details are presented in Table S1. Crystals were grown from vapour diffusion crystallizations. Crystals were selected, mounted using viscous onto a plastic mesh and cooled to the data collection temperature, 100 K. Data were collected on a Bruker Apex II CCD diffractometer with Mo-Kα radiation (λ = 0.71073 Å). Unit cell parameters were obtained from fast scan data frames, 1°/s ω, of an Ewald hemisphere. The unit-cell parameters and systematic absences in the diffraction data were uniquely consistent in each case for $P2_1/c$. The data were treated with numerical absorption corrections.[13] Structures were solved in Olex2[14] using intrinsic phasing methods[15] and refined with full-matrix, least-squares procedures on F^2.[16]

All non-hydrogen atoms were refined with anisotropic displacement coefficients. Hydrogen atoms were treated as idealized contributions with geometrically calculated positions and with U_{iso} equal to 1.2 U_{eq} (1.5 U_{eq} for methyl) of the attached atom. Atomic scattering factors are contained in the SHELXTL program library.[15] The structures have been deposited at the Cambridge Structural Database under the following CCDC depositary numbers: CCDC 2495987 and 2495988.

Computational Calculations and Analysis

All density functional theory (DFT) calculations were performed using the Gaussian 16 software package. The computational methodology was benchmarked by calculating the first reduction potential of complex **3** using several exchange–correlation functionals (as shown in Table S3) and a Born–Haber thermodynamic cycle method reported in literature.[17, 18] Based on agreement between the calculated reduction potentials and structural parameters, and experimental data (Table S3), the B3LYP functional was selected for all subsequent calculations. Geometry optimizations of complexes $\mathbf{1^{Me}}$, **2**, **3**, and all proposed intermediates were carried out at the B3LYP/def2-TZVP level of theory. Solvent effects were incorporated using the SMD continuum solvation model with DMF as the solvent. Frequency calculations were performed at the same level of theory to confirm each optimized structure as a true minimum (no imaginary frequencies) and to obtain thermal corrections to the Gibbs free energy. Open-shell species were treated using unrestricted DFT, and multiple spin states were considered where appropriate.

Energy Decomposition Analysis coupled with Natural Orbitals for Chemical Valence (EDA-NOCV)[19-21] analyses were further carried out for the optimized CO_2-bound intermediates of complexes **2** and **3** using ORCA 6.1.1 by dividing each structure into the Cu–ligand fragment and the CO_2 unit.[22] The total interaction energy was decomposed into orbital, electrostatic, Pauli repulsion, exchange–correlation, and solvation contributions, while the NOCV channels were used to compare the dominant Cu/ligand–CO_2 orbital interactions.[23]

Table S1. Crystal Data and Structure Refinement for complexes 2 and 3

	**2**	**3**
Formula	$C_{22}H_{21}CuN_3O_3S_2$	$C_{21}H_{15}CuN_3O_2S_2$
M.W.	503.08	469.02
Crystal system	Monoclinic	Monoclinic
Space group	$P2_1/c$	$P2_1/c$
CCDC numbers	2495988	2495987
Temperature	100 K	100 K
a/Å	13.8234(4)	12.2254(4)
b/Å	18.6655(6)	12.3037(4)
c/A^0	8.2949(2)	12.4406(4)
$\alpha/^0$	90	90
$\beta/^0$	99.6310(10)	99.7120(10)
$\gamma/^0$	90	90
Volume/$Å^3$	2110.09(10)	1844.47(10)
Z	4	4
ρ_{calc} (mg/mm^3)	1.584	1.689
μ/mm^{-1}	1.264	1.435
F(000)	1036.0	956.0
Crystal size/mm^3	0.75 × 0.52 × 0.366	0.359 × 0.332 × 0.262
Reflection collected	84207	66655
Goodness-of-fit on F^2	1.064	1.069
Final R indexes [I>=2σ (I)]	R_1 = 0.0363, wR_2 = 0.1040	R_1 = 0.0307, wR_2 = 0.0788
Final R indexes [all data]	R_1 = 0.0403, wR_2 = 0.1059	R_1 = 0.0330, wR_2 = 0.0811

Supporting Results/Figures

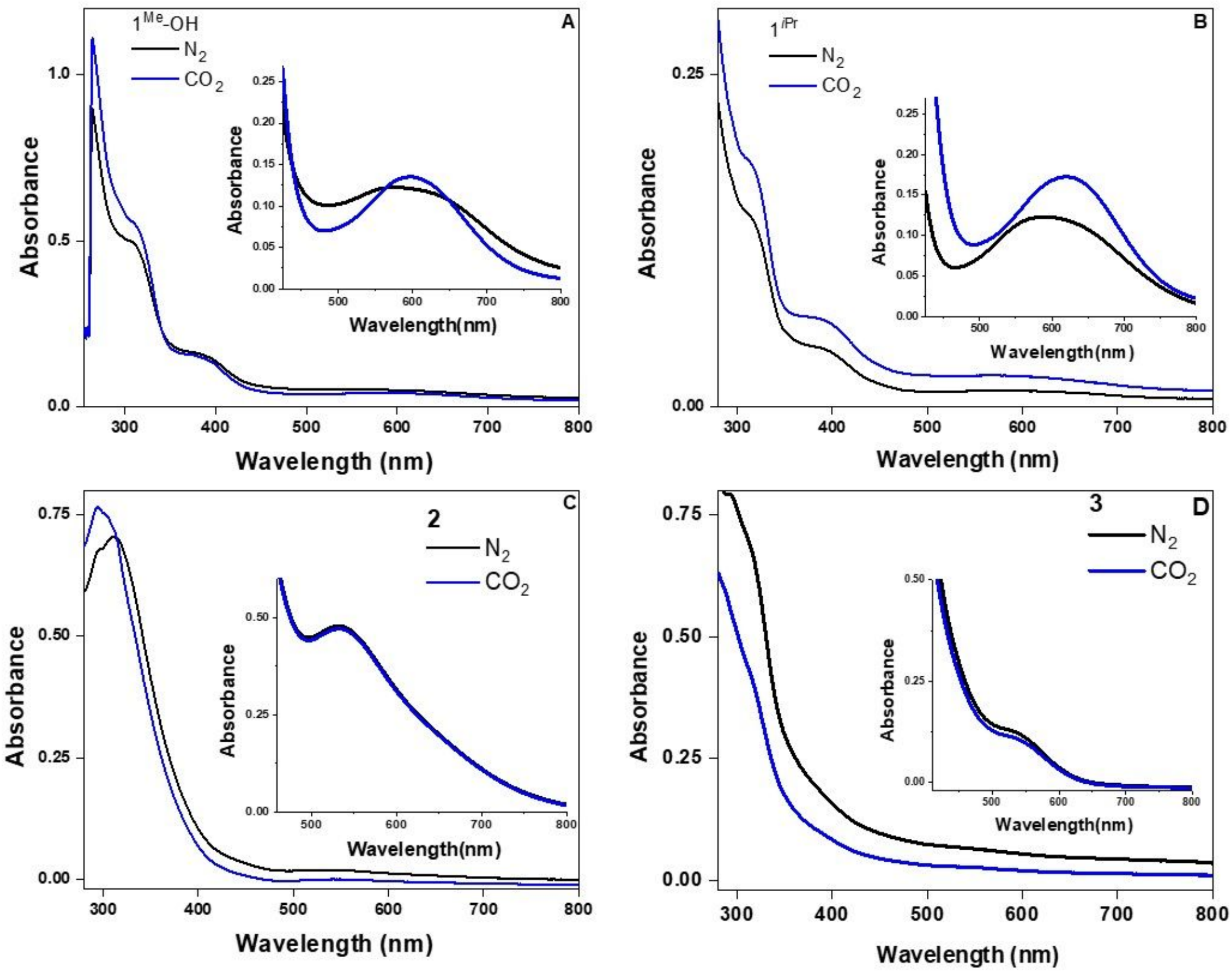

Figure S1. UV−vis spectra of copper complexes in the presence of N_2 (black line) and carbon dioxide (Blue line) in DMF solution. A) **1^Me^-**OH (0.1 mM); B) **1*^iPr^*** (0.06 mM); C) **2** (0.1 mM) and D) **3** (0.1 mM) and Inset A) **1^Me^-**OH (1 mM); B) **1*^iPr^*** (1 mM); C) **2** (1.3 mM) and D) **3** (1 mM).

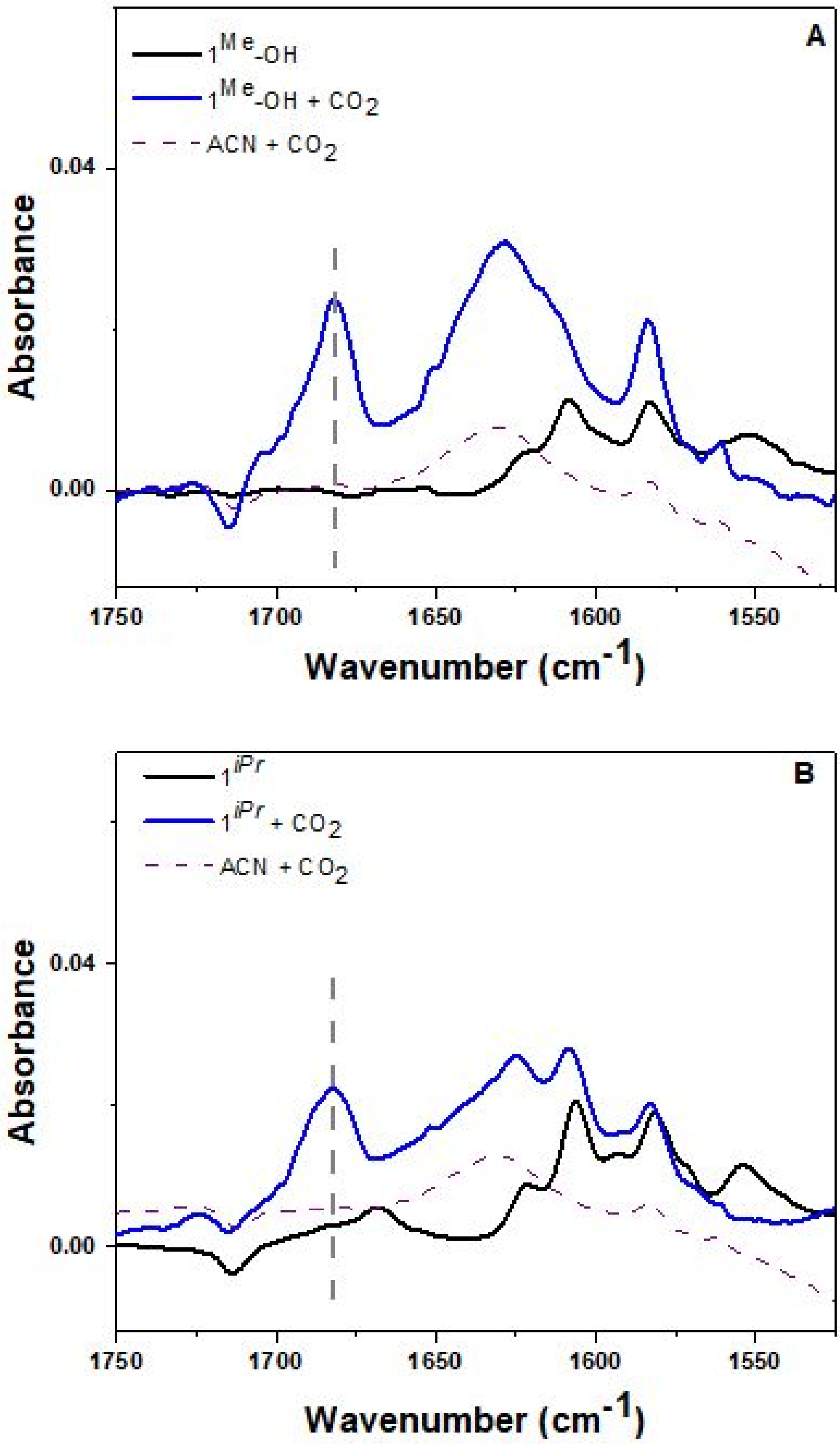

Figure S2. The solution state IR spectra in acetonitrile (ACN) of complexes A): **1^{Me}-OH** (black); **1^{Me}-OH** + CO_2 (blue); and B): **1^{iPr}** (black); **1^{iPr}** + CO_2 (blue) and CO_2-saturated ACN solvent (dashed purple) respectively. All measurements were conducted in ACN under the controlled conditions.

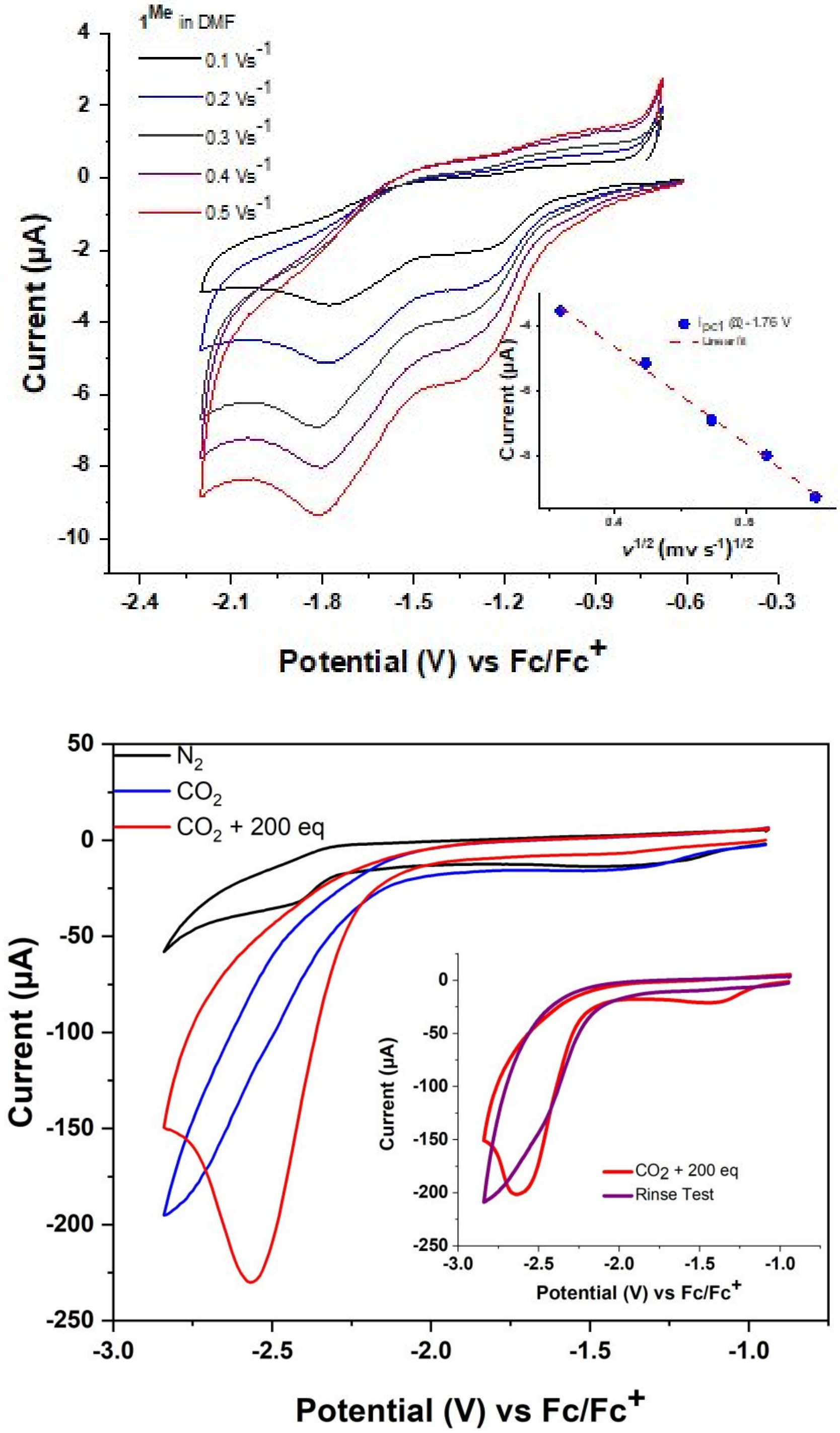

Figure S3. Cyclic voltammograms of complex **1**[Me] (1 mM in DMF) recorded using 0.1 M $TBAPF_6$ as the supporting electrolyte. (Top) Dependence of the second redox peak current on the square root of the scan rate, indicating diffusion-controlled electron transfer. (Bottom) Voltammograms collected under N_2 (black), CO_2 (blue), and CO_2 and 200 equivalents of TFE (red). The purple trace represents the rinse test, acquired in fresh DMF containing 0.1 M $TBAPF_6$ under 100 % CO_2 in the absence of **1**[Me]. The persistence of cathodic current in the rinse experiment suggests the formation of heterogeneous copper species on the electrode surface during electrolysis.

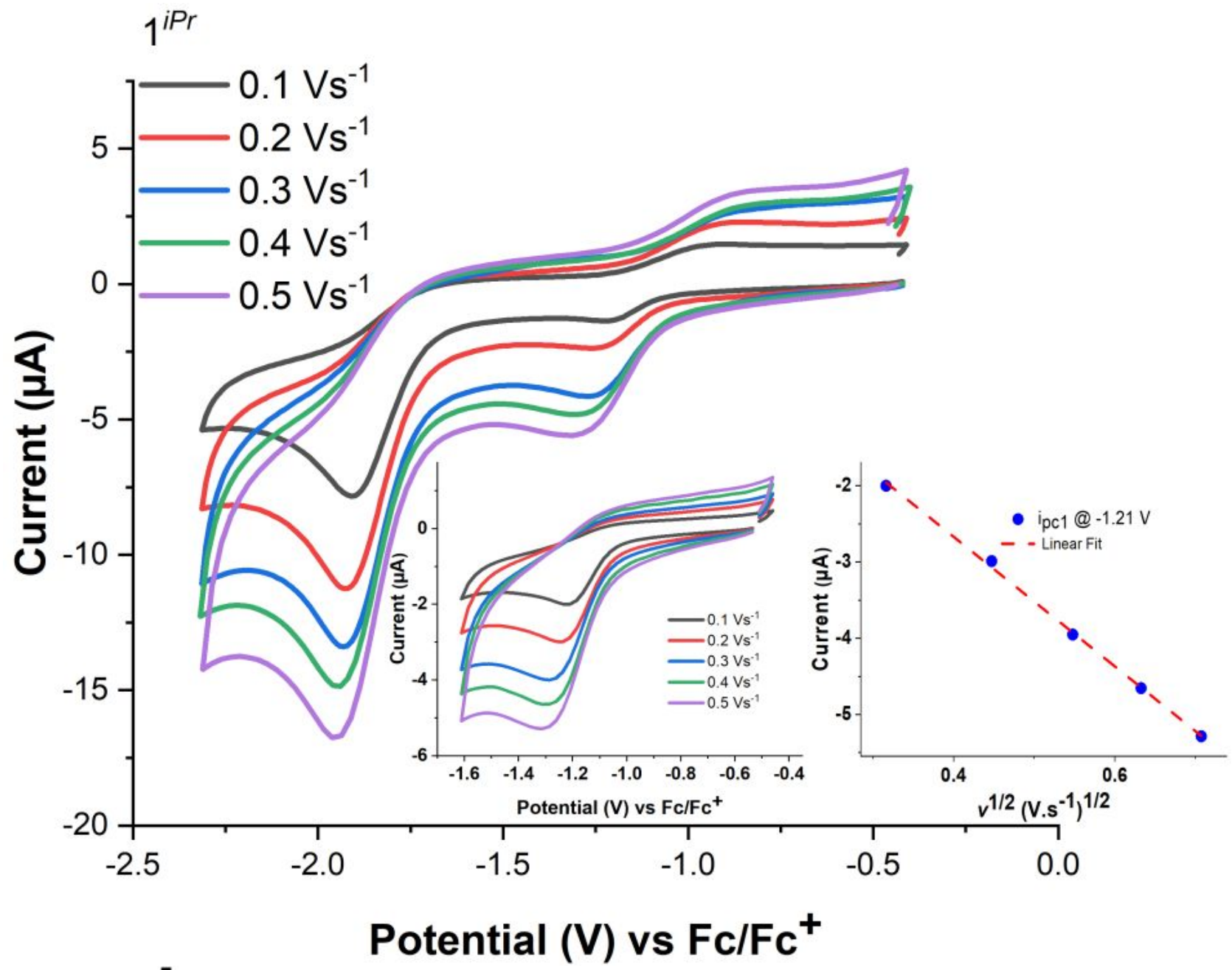

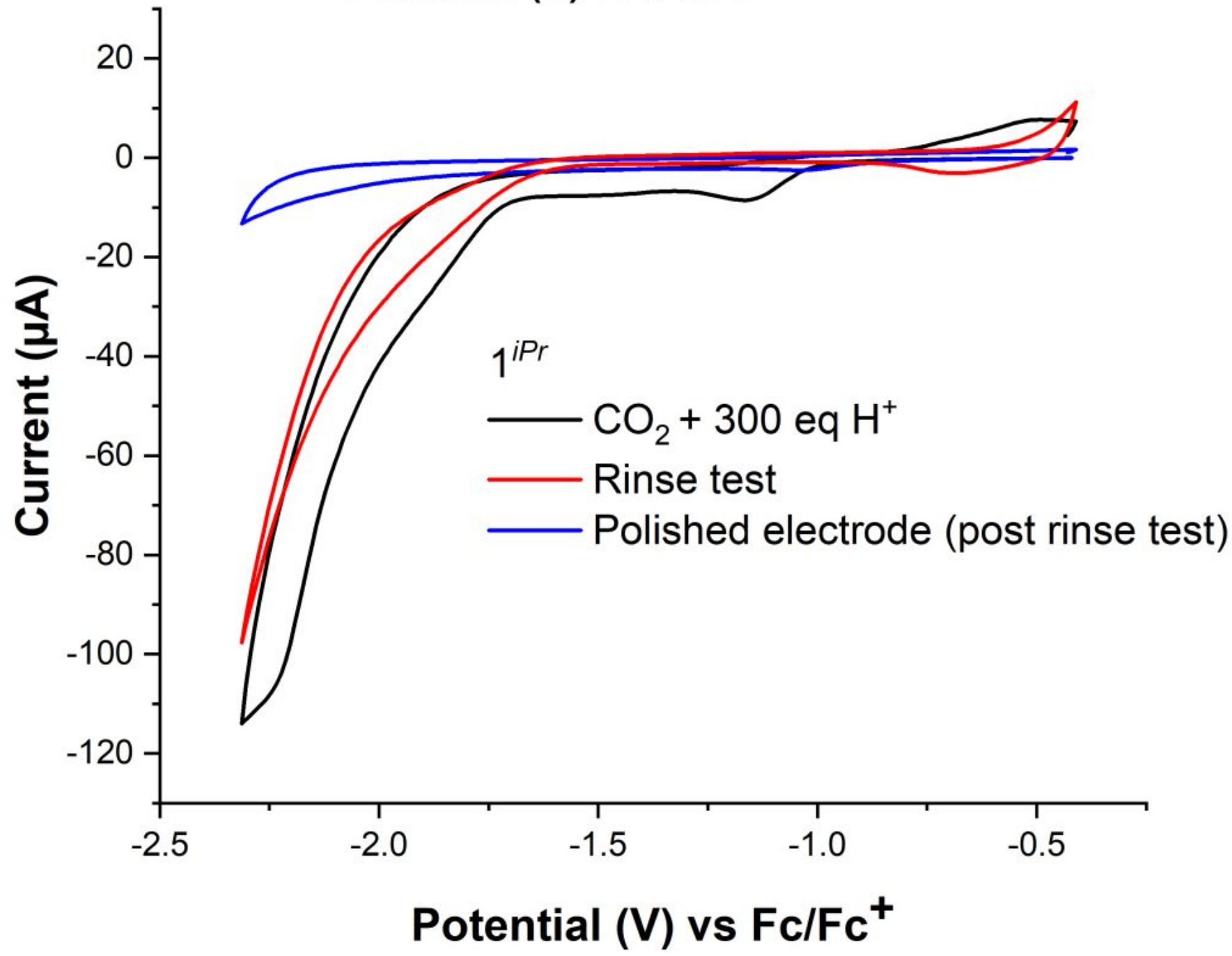

Figure S4. Cyclic voltammograms of complex $\mathbf{1^{\textit{i}Pr}}$ (1 mM in DMF) recorded using 0.1 M $TBAPF_6$ as the supporting electrolyte. (Top) The inset plots illustrate the irreversible nature of the first redox peak and the dependence of the peak current on the square root of the scan rate, indicating the diffusion-controlled behavior. (Bottom) Voltammograms collected under CO_2 with 300 equivalents of TFE (black), under the rinse test condition (red), and after mechanical polishing of the electrode (blue). The persistence of cathodic current in the rinse test suggests the formation of heterogeneous copper species on the electrode surface during electrolysis.

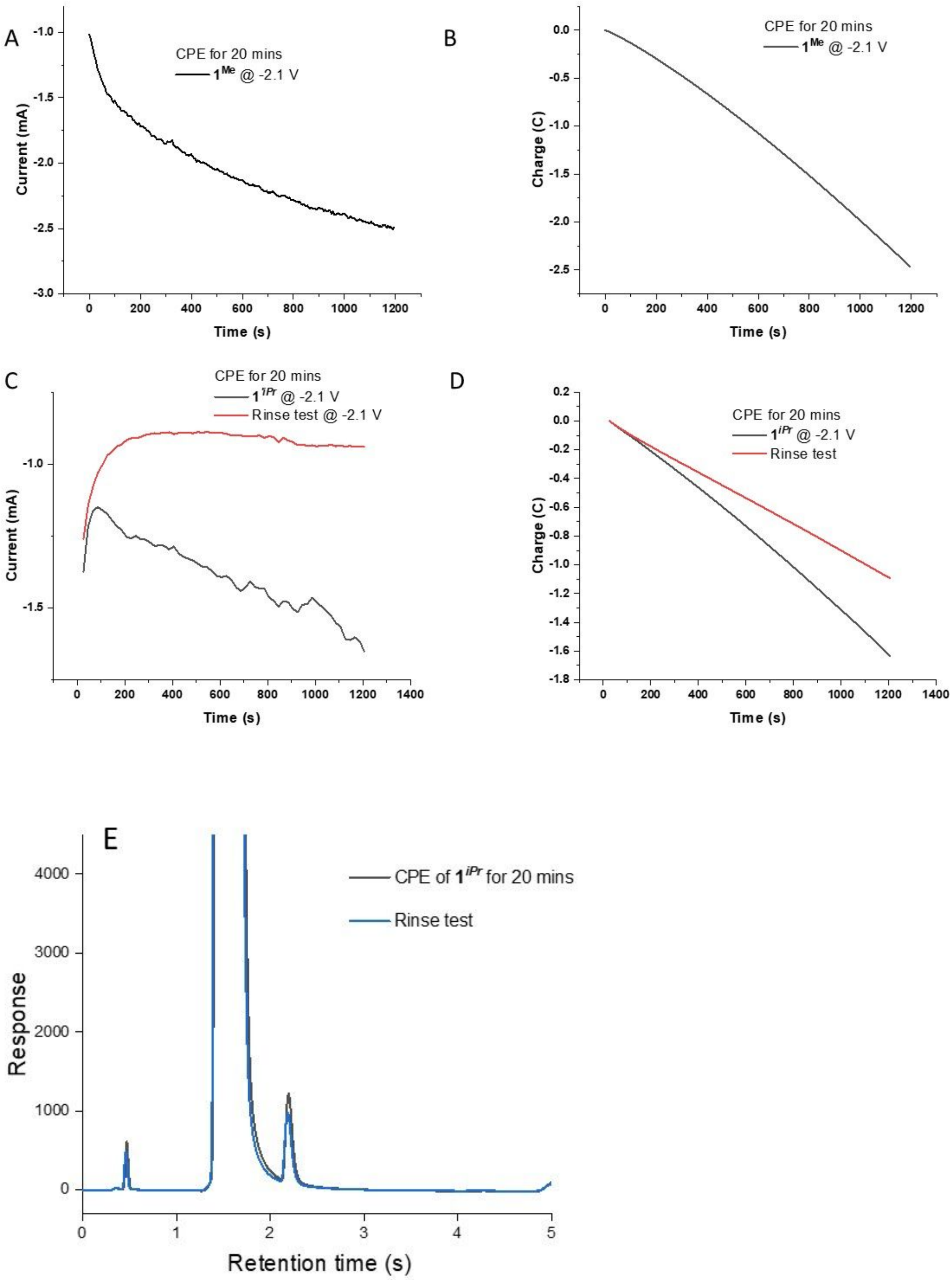

Figure S5. Bulk electrolysis of Complex $\mathbf{1^{Me}}$ and $\mathbf{1^{\textit{i}Pr}}$ (0.5 mM) under a CO_2 atmosphere in the presence of TFE (150 mM) in DMF for 20 minutes A) $\mathbf{1^{Me}}$ current vs. time and B) corresponding charge vs. time plot; C) $\mathbf{1}\ ^{\mathbf{\textit{i}Pr}}$ current vs. time and D) corresponding charge vs. time plot. E) Gas chromatograph of headspace products following electrolysis of $\mathbf{1^{\textit{i}Pr}}$ at –2.1 V for 20 mins. Rinse Test refers to bulk electrolysis experiment in a fresh CO_2-saturated DMF solution using the post-electrolysis electrode after rinsing (in the absence of complex $\mathbf{1^{\textit{i}Pr}}$).

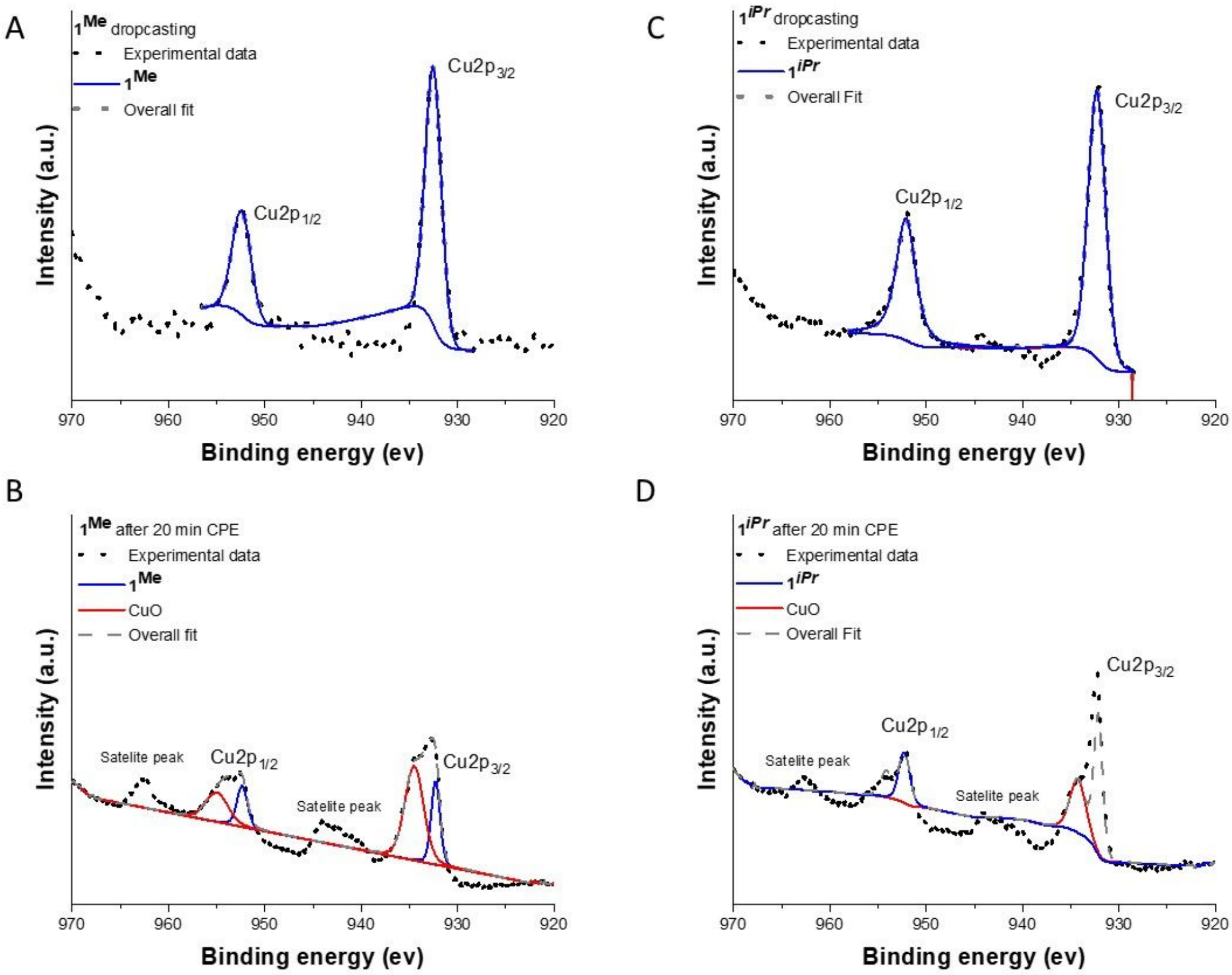

Figure S6. The XPS analysis of the Cu 2p region of the complexes **1^Me^** and **1^*i*Pr^**. A) and C) for drop-cast complex **1^Me^** and **1^*i*Pr^** on the glass carbon electrode. The Cu 2p fitting component was achieved using the drop-cast **1^Me^** and **1^*i*Pr^** respectively. B) and D) XPS analysis of the Cu 2p region for complex **1^Me^** and **1^*i*Pr^** after electrolysis. The fitting of Cu 2p peak components was achieved using the reference spectrum from drop-cast **1^Me^** and **1^*i*Pr^** signals and reference [24] for the remaining copper species.

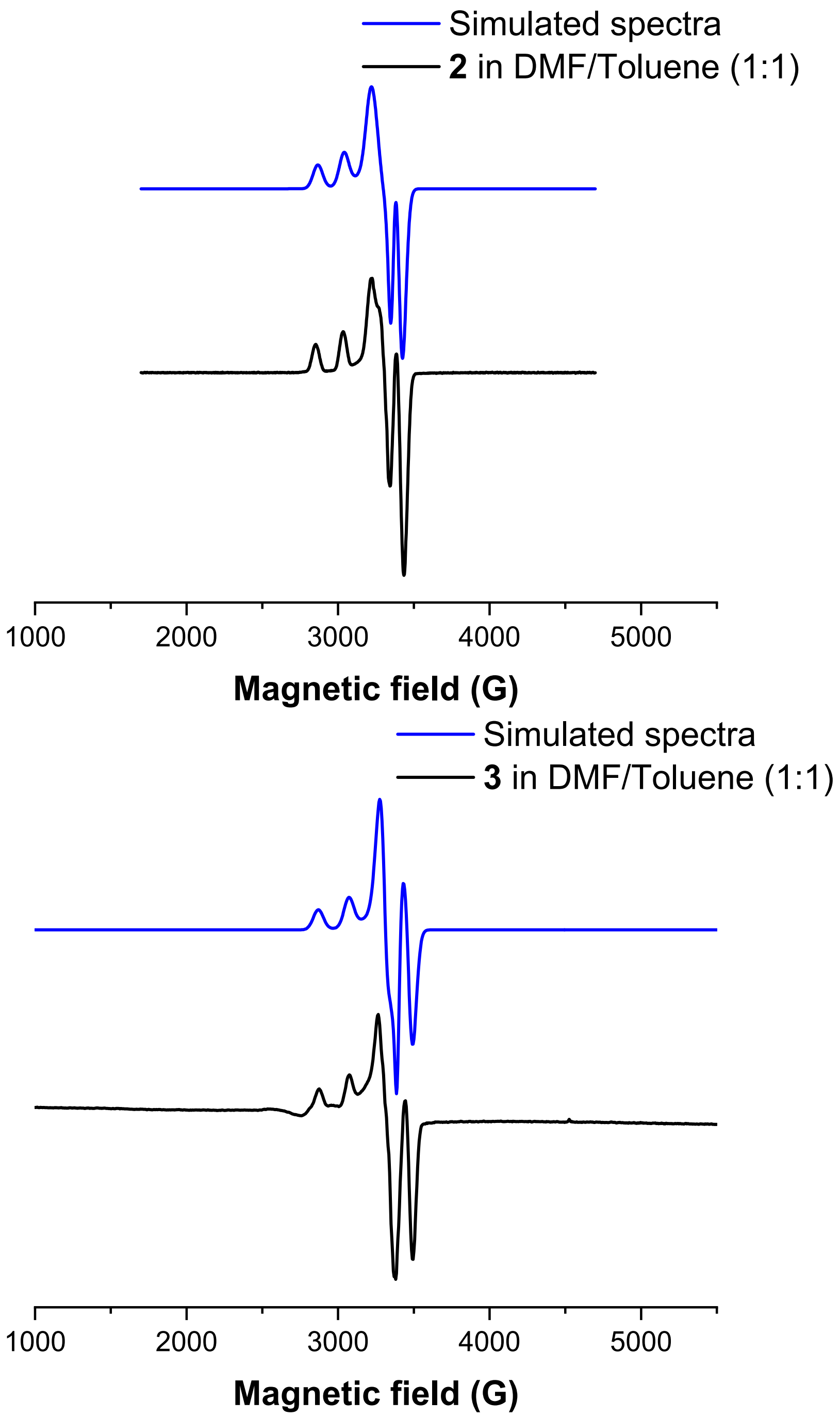

Figure S7. The EPR spectra (black) of complex **2** and **3** (1 mM) in DMF/Toluene respectively were recorded at 77 K with a microwave frequency of 9.4942 GHz. The corresponding simulated spectra of the complexes are shown in blue.

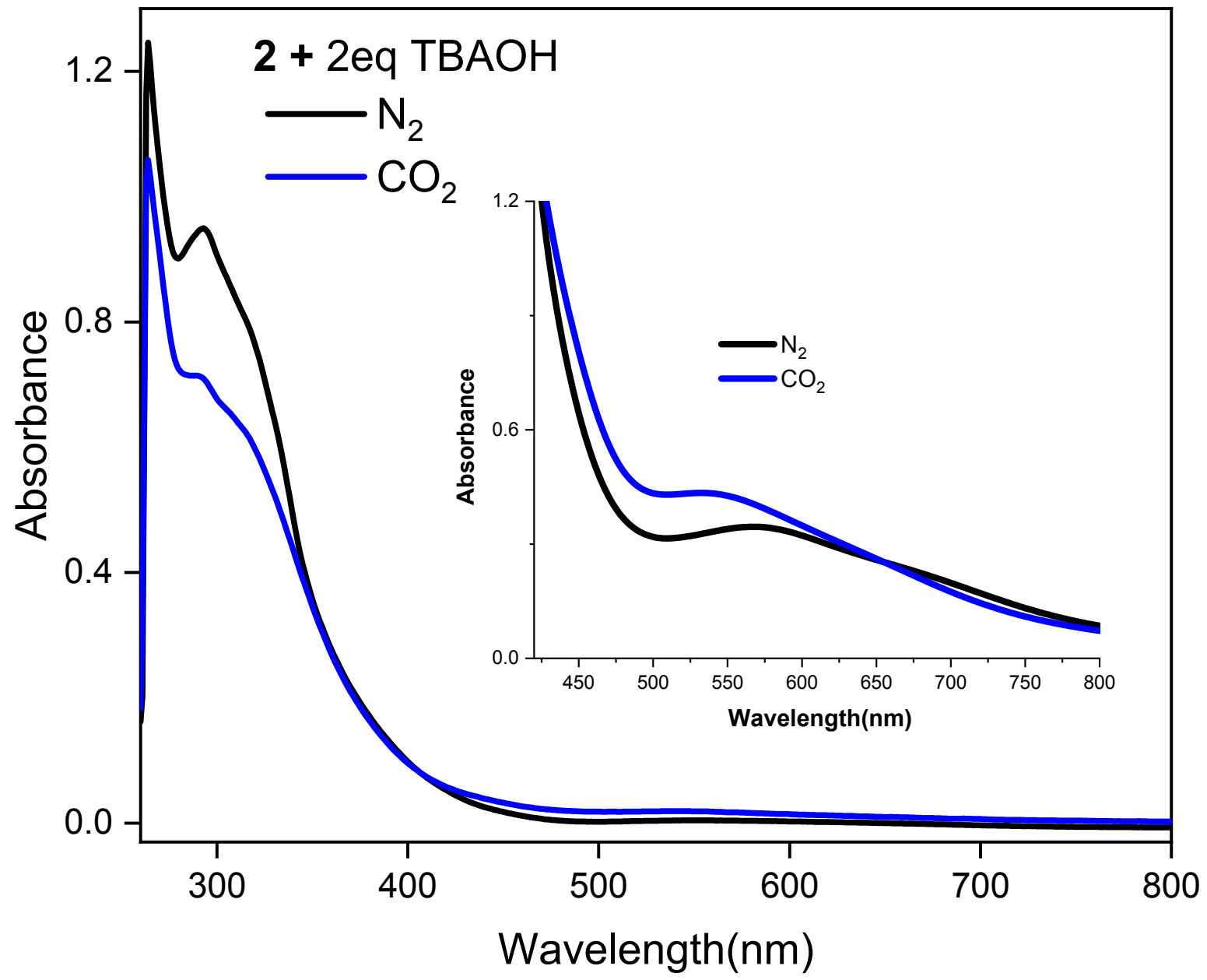

Figure S8. Reactivity of complex **2** (0.031 mM) in the presence of two equivalents of TBAOH in DMF under N_2 and CO_2 atmosphere respectively. Inset: the d–d transition of **2** (1.2 mM).

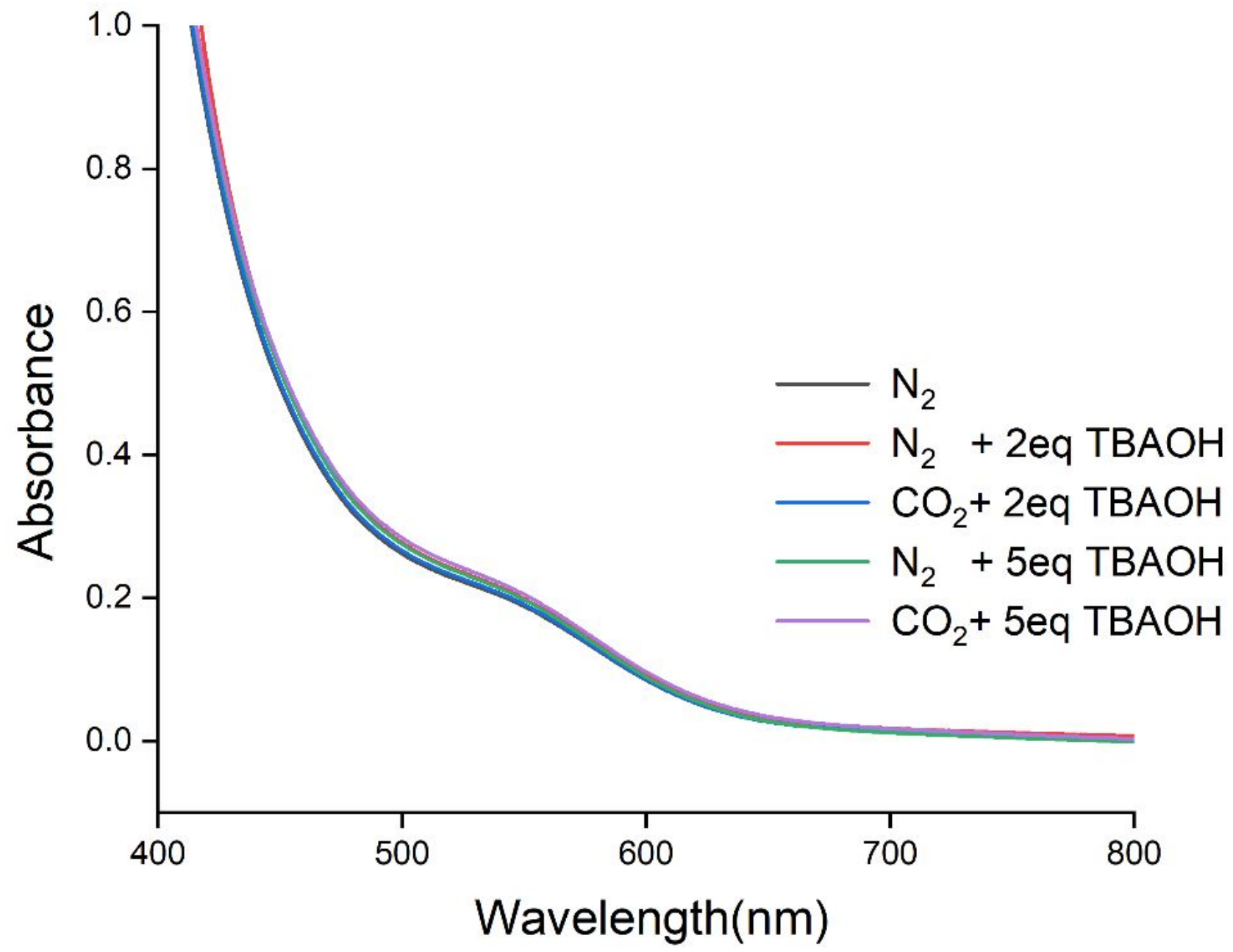

Figure S9. Reactivity of complex **3** (1 mM) with varying equivalents of TBAOH in DMF under N_2 and CO_2 atmosphere respectively.

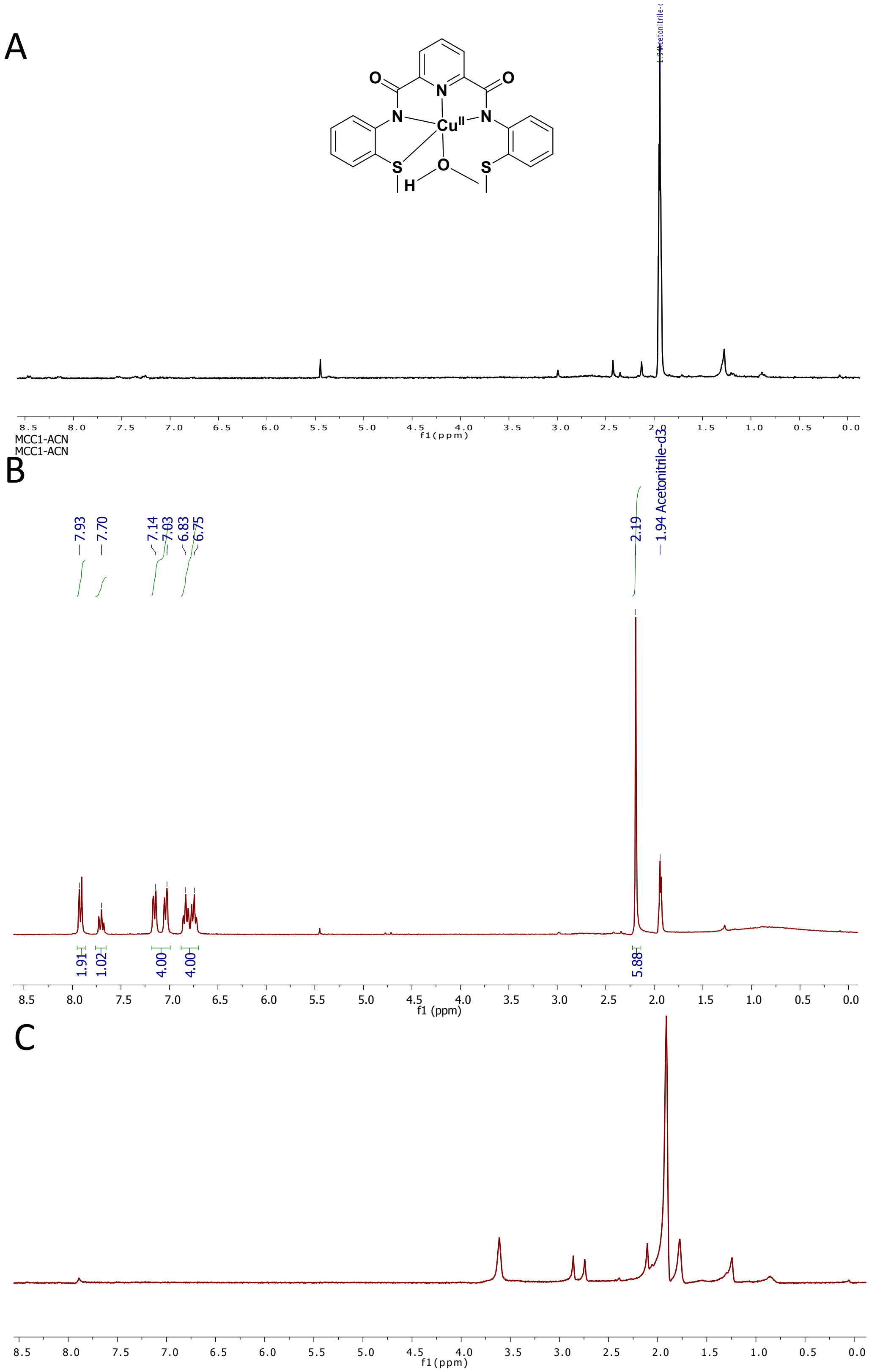

Figure S10. ^{1}H NMR spectra of A) Complex **2** in N_2; B) **2** with 1.5 equivalents of cobaltocene in N_2 and C) **2** with 1.5 equivalents of cobaltocene in CO_2 in CD_3CN.

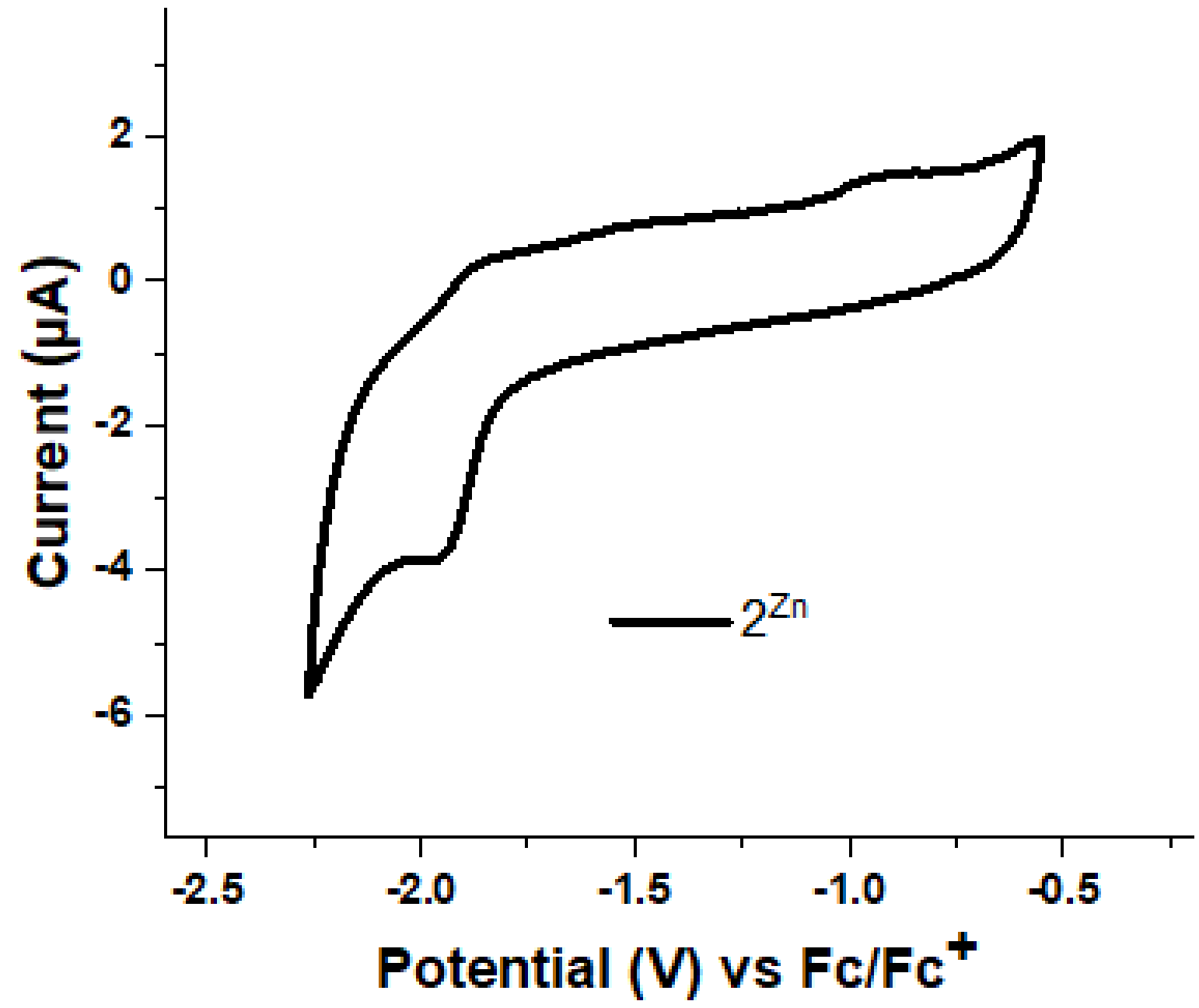

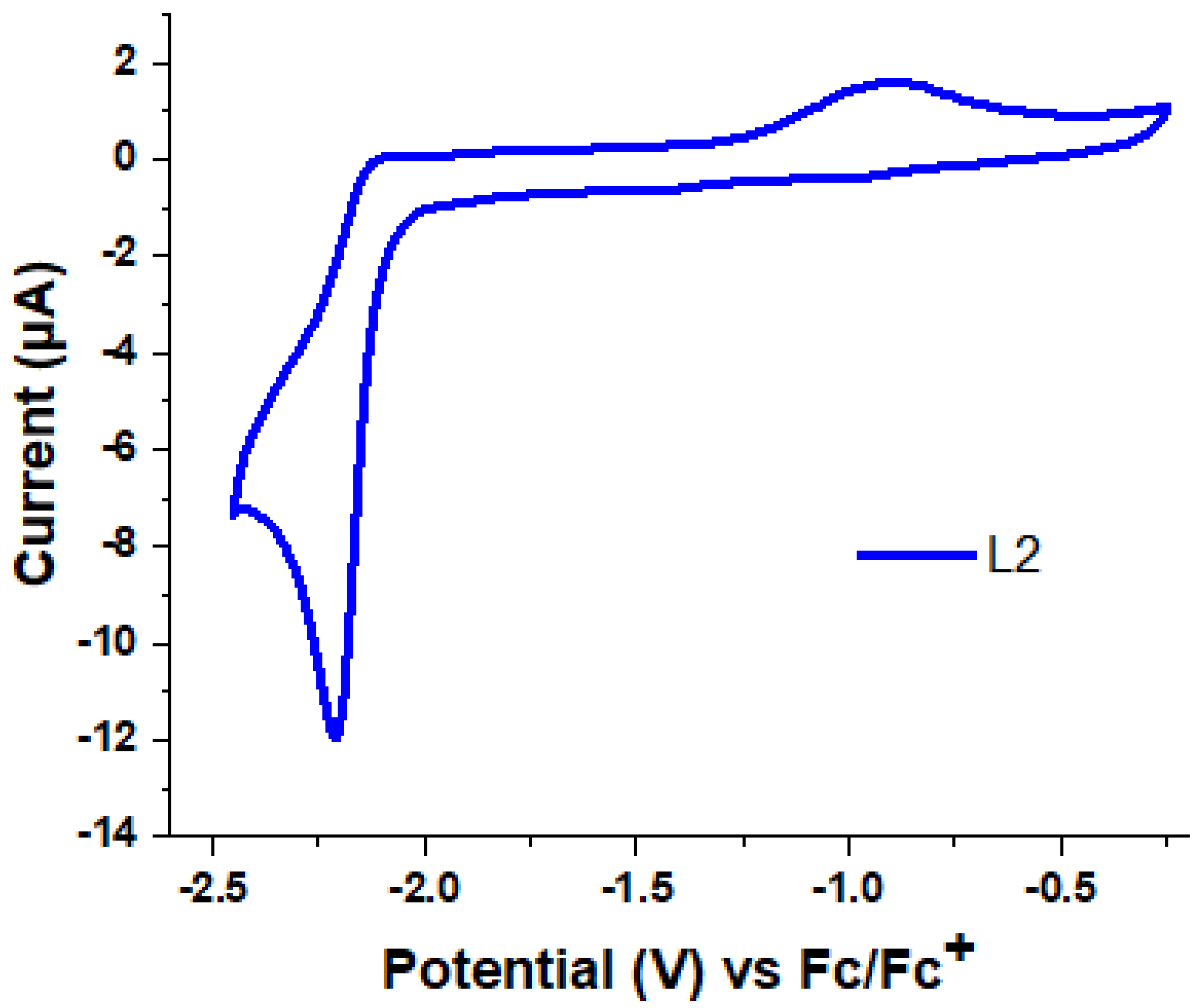

Figure S11. The cyclic voltammogram of (Top) 1 mM complex $\mathbf{2^{Zn}}$; (Bottom) 2 mM of **L2** in DMF using $TBAPF_6$ as supporting electrolyte in the presence of N_2. scan rate: 0.1 Vs^{-1}.

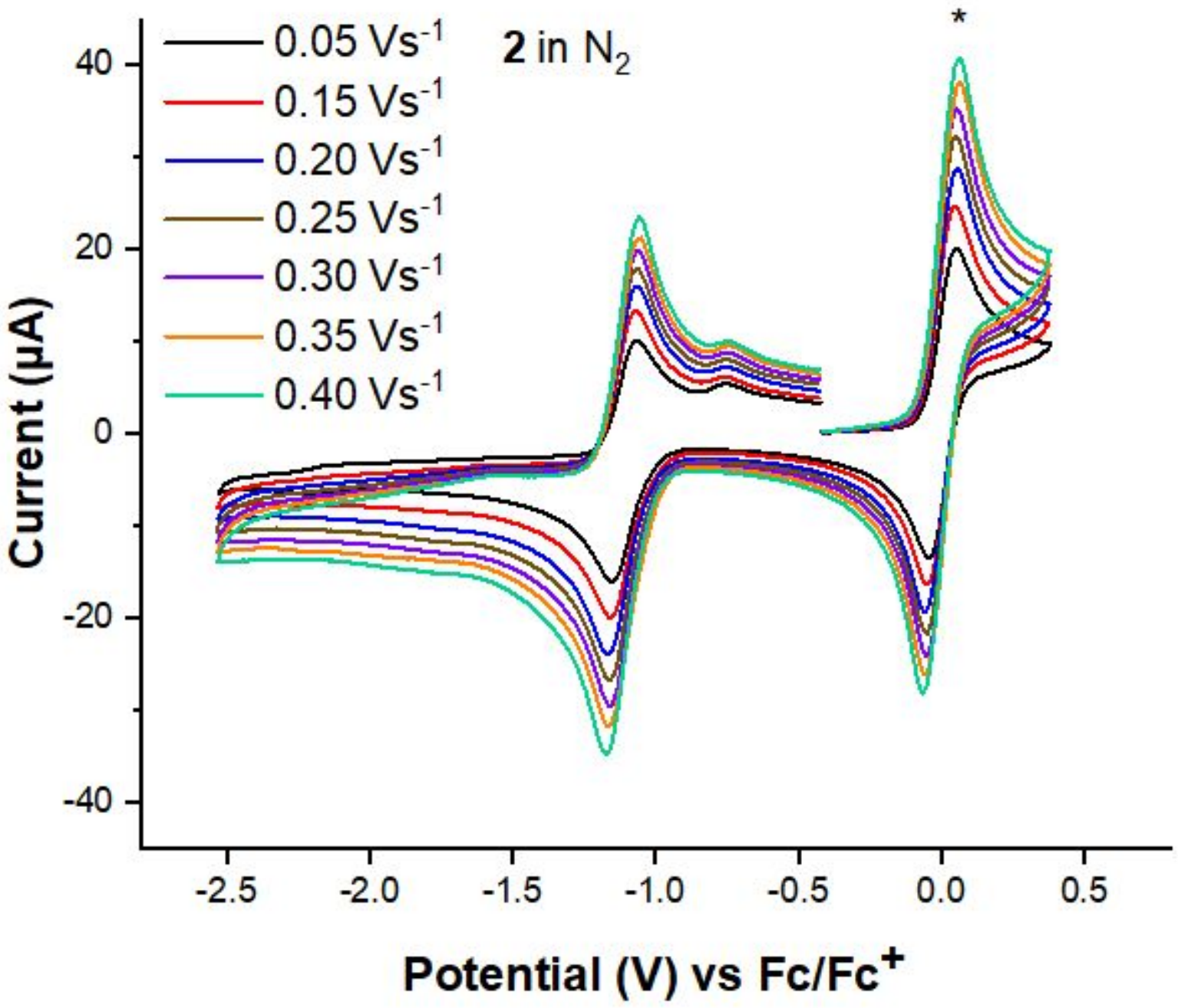

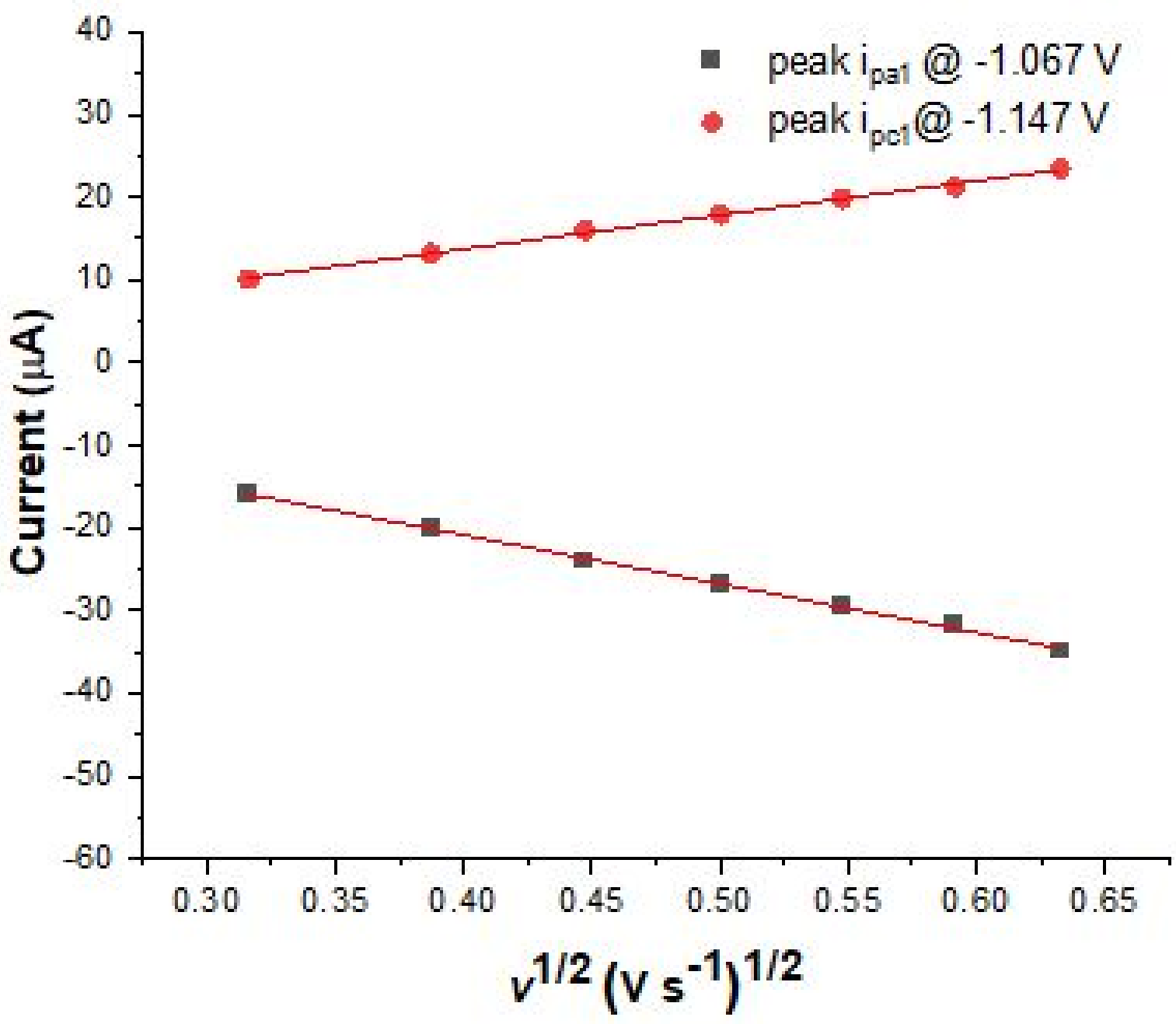

Figure S12. (Top) The cyclic voltammograms of **2** (1 mM in DMF) in the presence of N_2. The asterisk sign refers to the redox peak from internal standard samples, ferrocene. (bottom) Plots of i_p versus $v^{1/2}$.

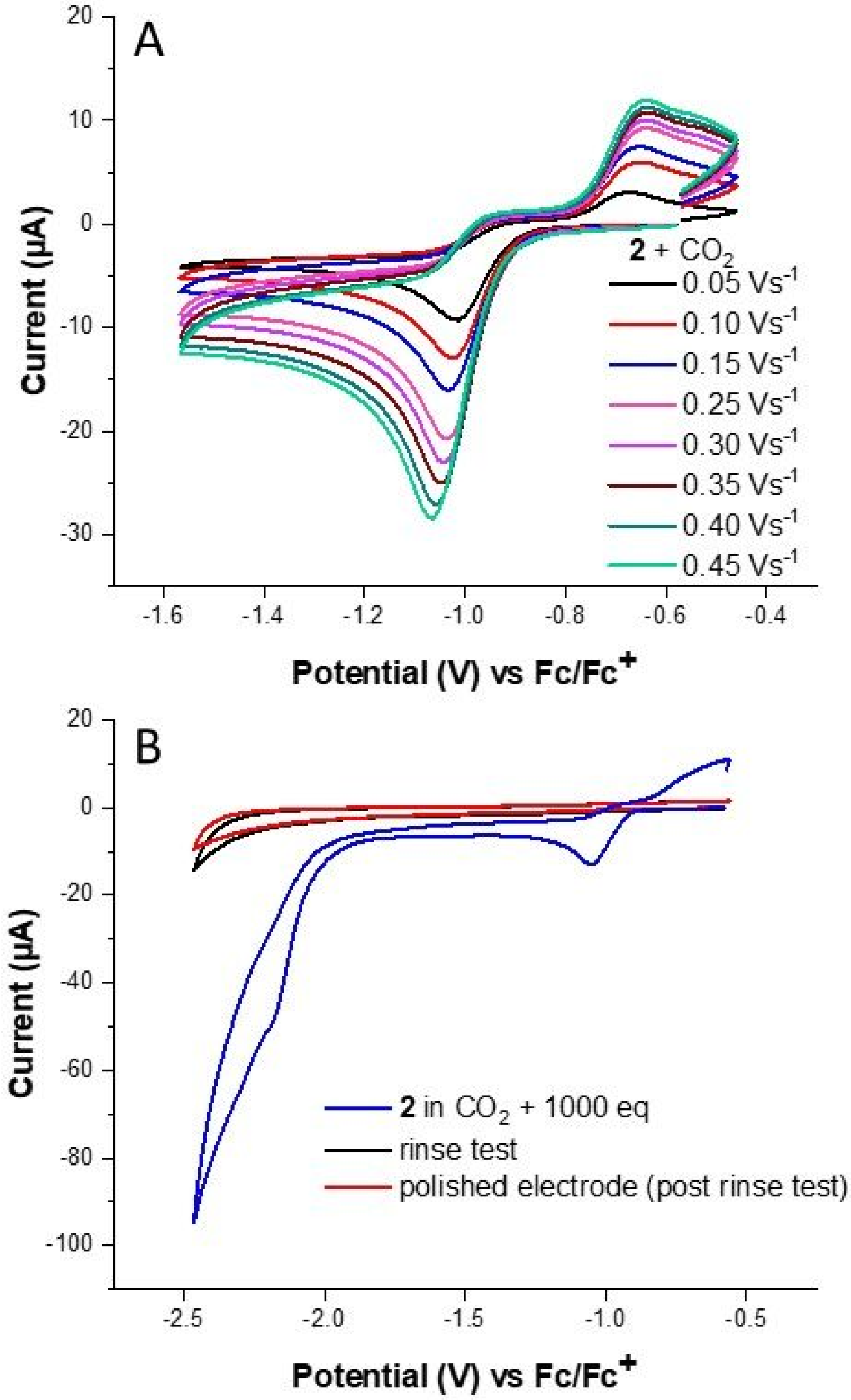

Figure S13. A) The cyclic voltammograms of **2** (1 mM) in CO_2 with various scan rates in 0.1 M $TBAPF_6$/DMF. B) Rinse test of cyclic voltammograms of complex **2**. TFE was used as proton source. Scan rate: 0.1 Vs^{-1}.

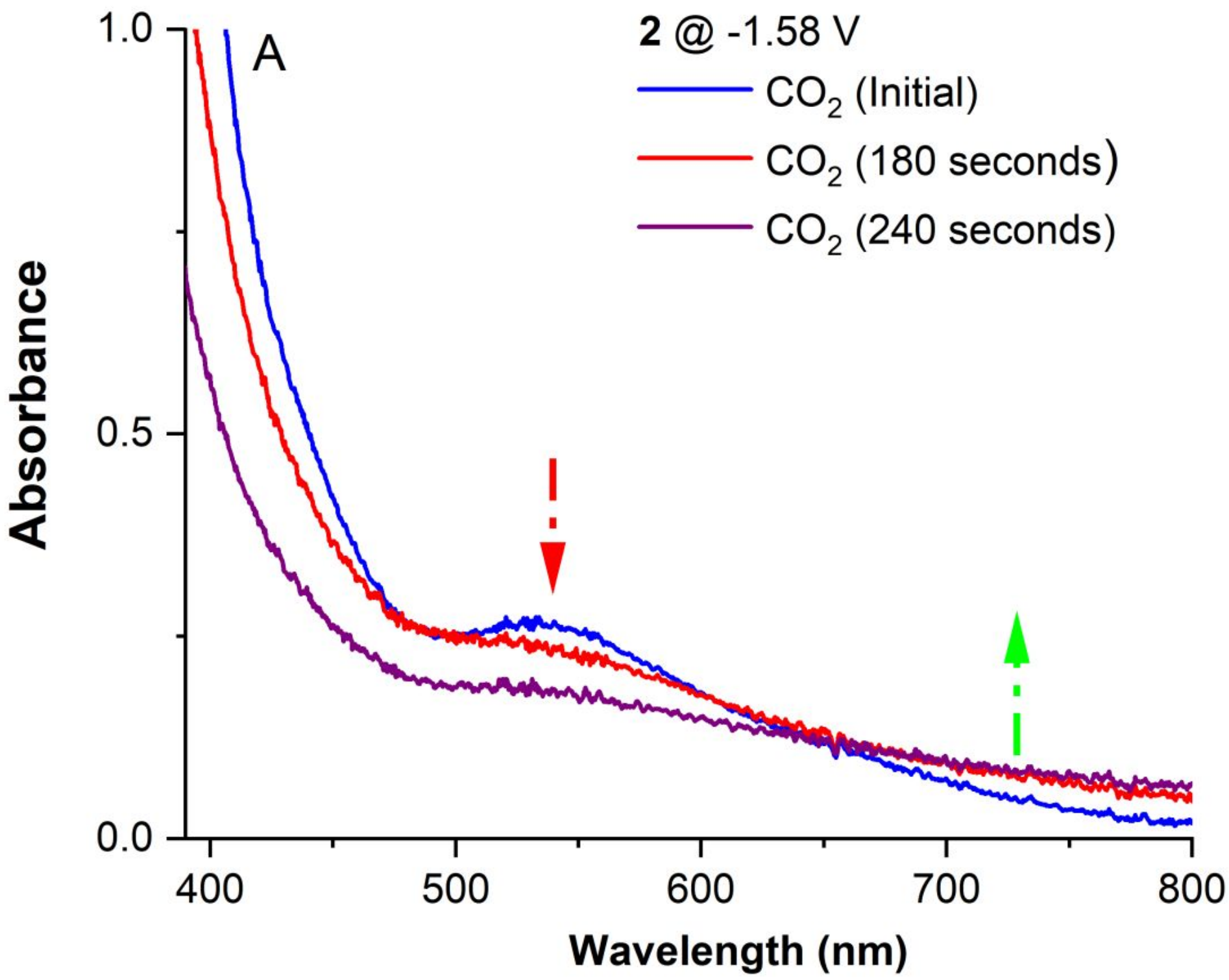

Figure S14. Spectroelectrochemical UV–Vis spectra of complex **2** recorded under CO_2 atmosphere in DMF at −1.58 V.

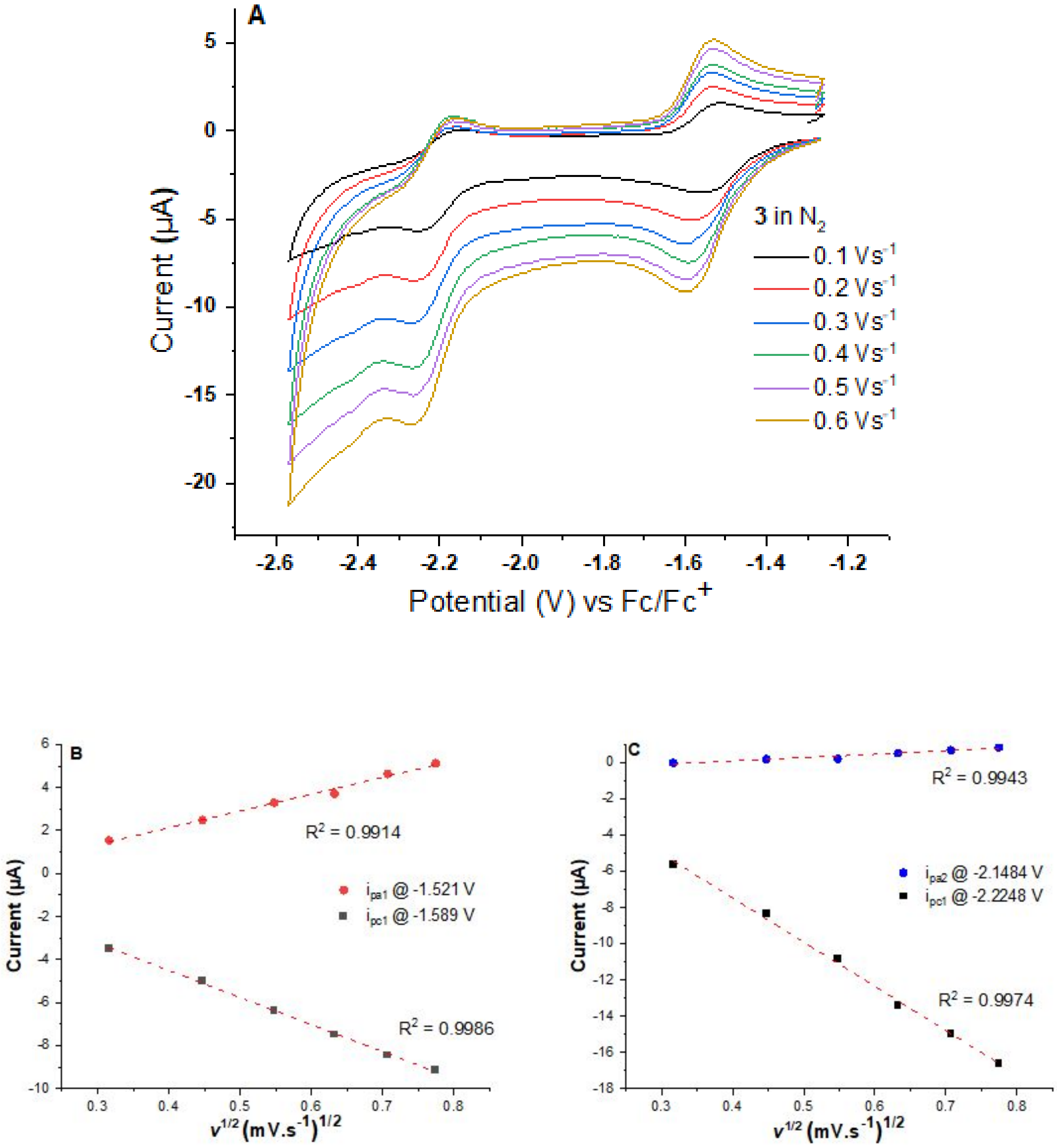

Figure S15. A) Cyclic voltammograms of compound **3** (1 mM in DMF) recorded at various scan rates in 0.1 M $TBAPF_6$/DMF; B) Plots of the first redox peak current versus $v^{1/2}$; C) Plots of the second redox peak current versus $v^{1/2}$.

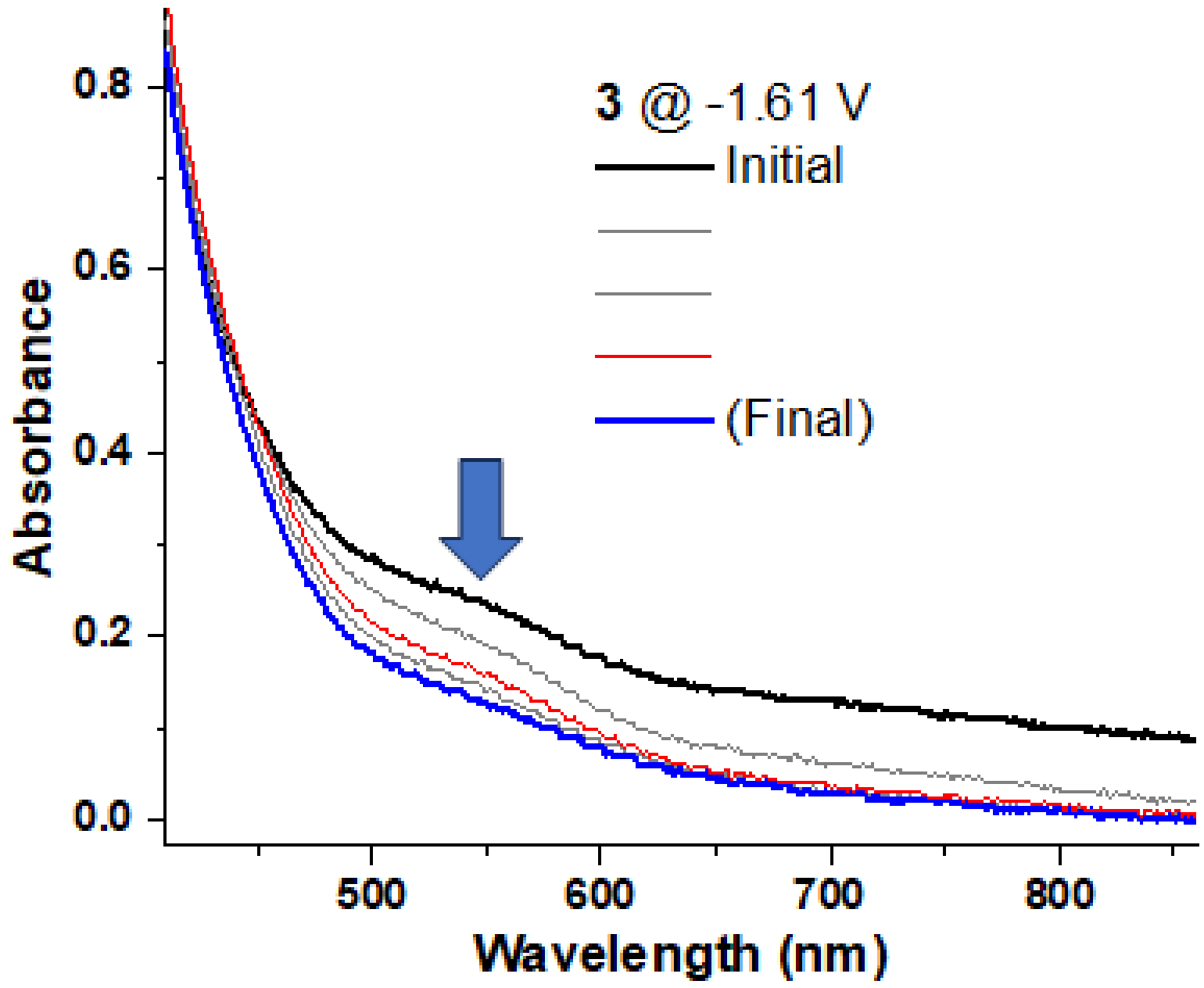

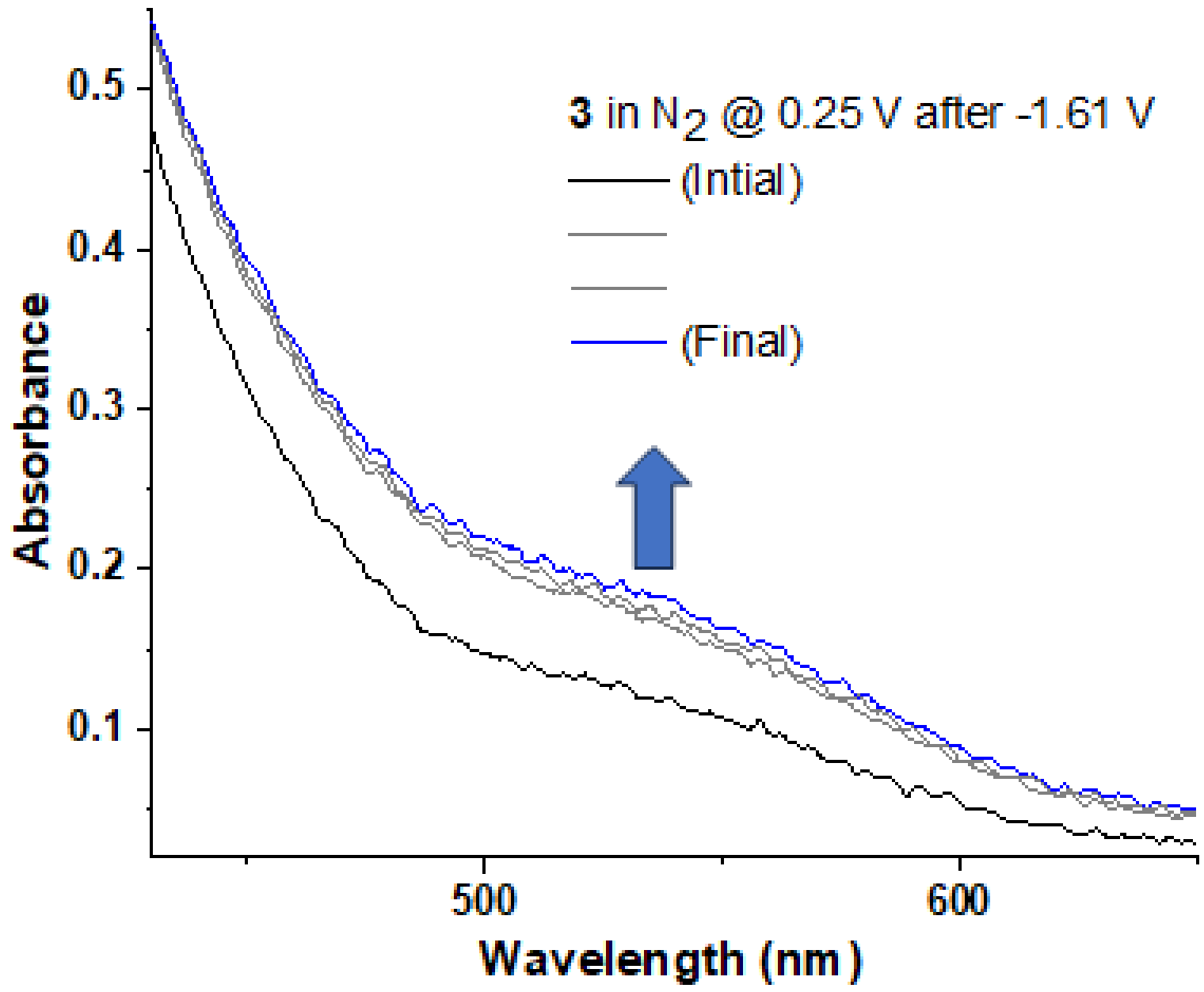

Figure S16. UV–Vis spectral changes of complex **3** under N_2 atmosphere in DMF during spectroelectrochemical measurements at −1.61 V (top) and subsequently poised at +0.25 V (bottom).

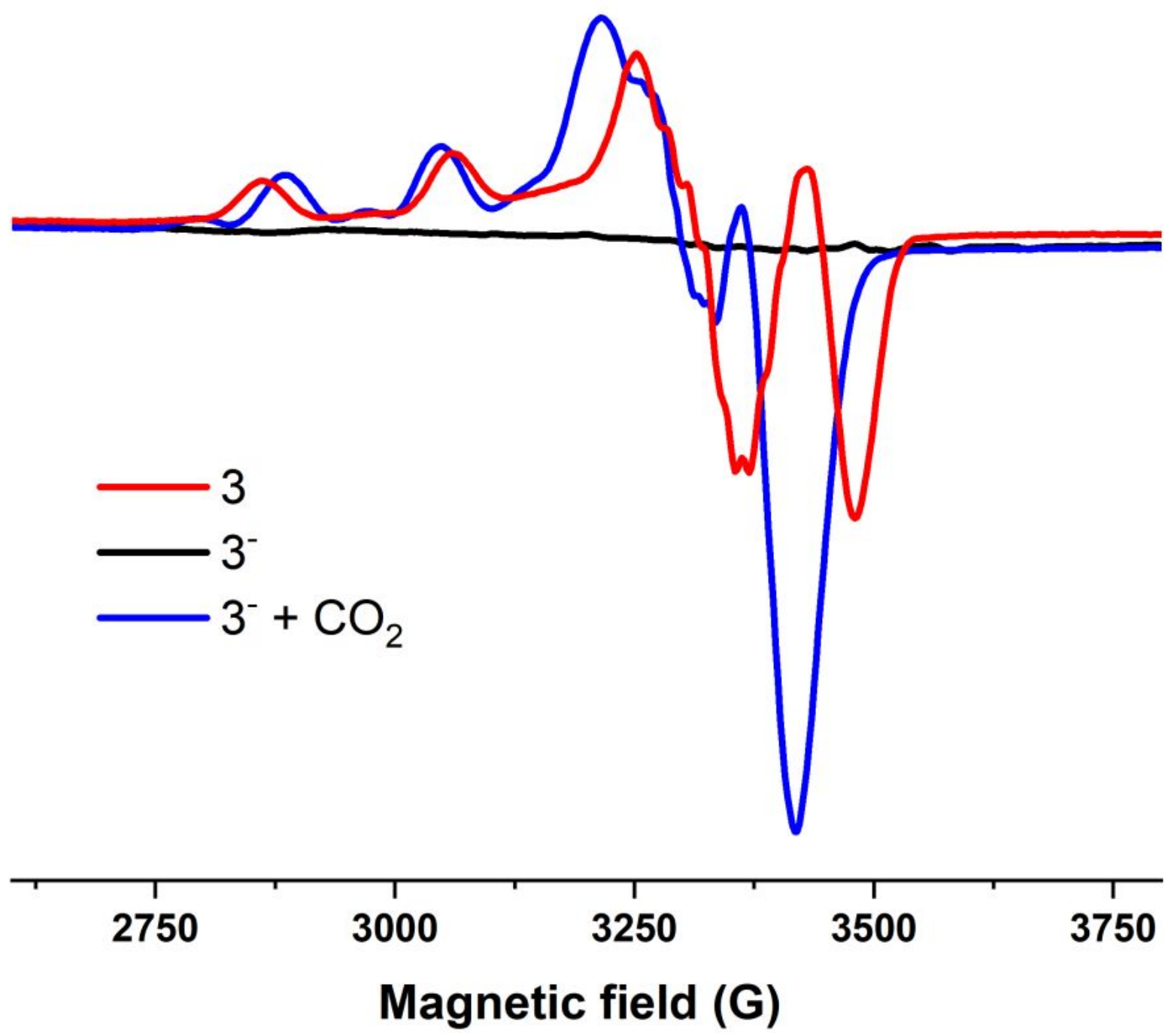

Figure S17. EPR spectra of complex **3** (red), $\mathbf{3}^-$ generated by **3** with 1.2 equivalents of $Co(Cp^*)_2$ (black) and $\mathbf{3}^-$ in CO_2 (blue) (1.5×10^{-3} M) in a DMF/toluene mixture. Measurement conditions: 9.474535 GHz, 77 K.

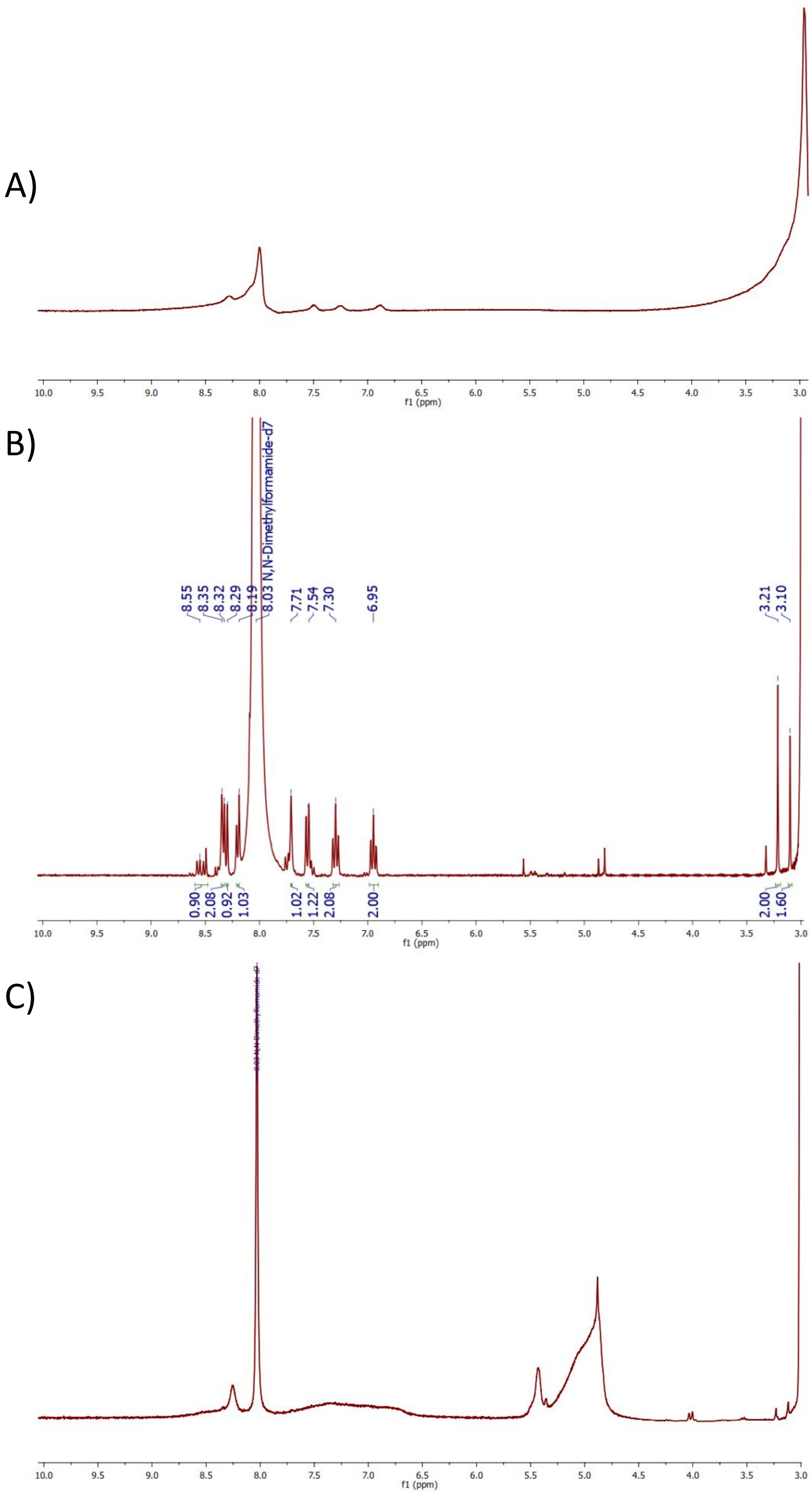

Figure S18. ^1H NMR spectra of A) Complex **3** in N_2; B) **3** with 1 equiv. of decamethylcobaltocene [Co(Cp*)$_2$] in N_2 C) **3** with 1 equiv. of [Co(Cp*)$_2$] in CO_2 in CD_3CN.

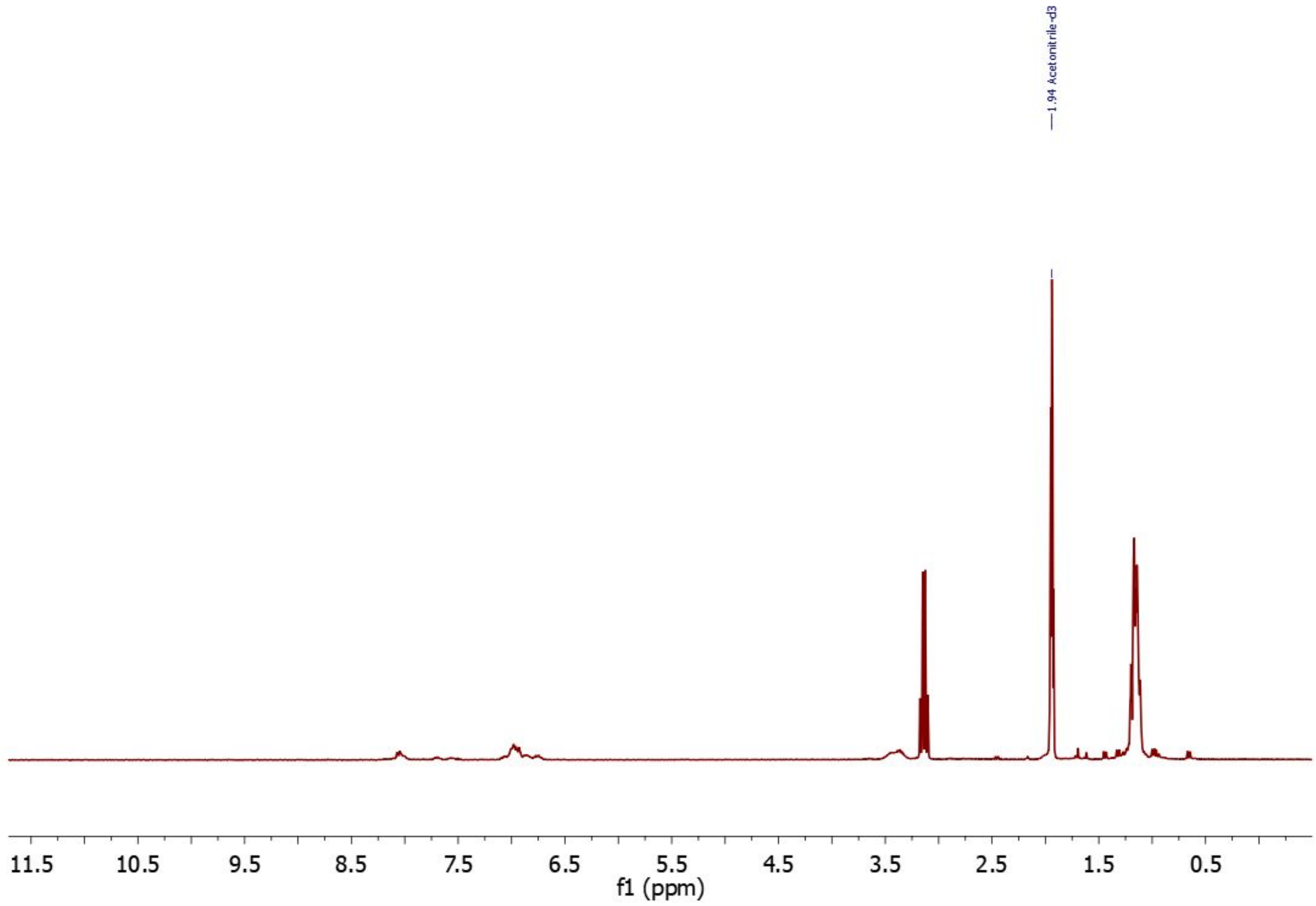

Figure S19. ^{1}H NMR spectra of Complex $\mathbf{1}^{\boldsymbol{i}\mathbf{Pr}}$ with 1.5 equivalents of cobaltocene in N_2 in CD_3CN.

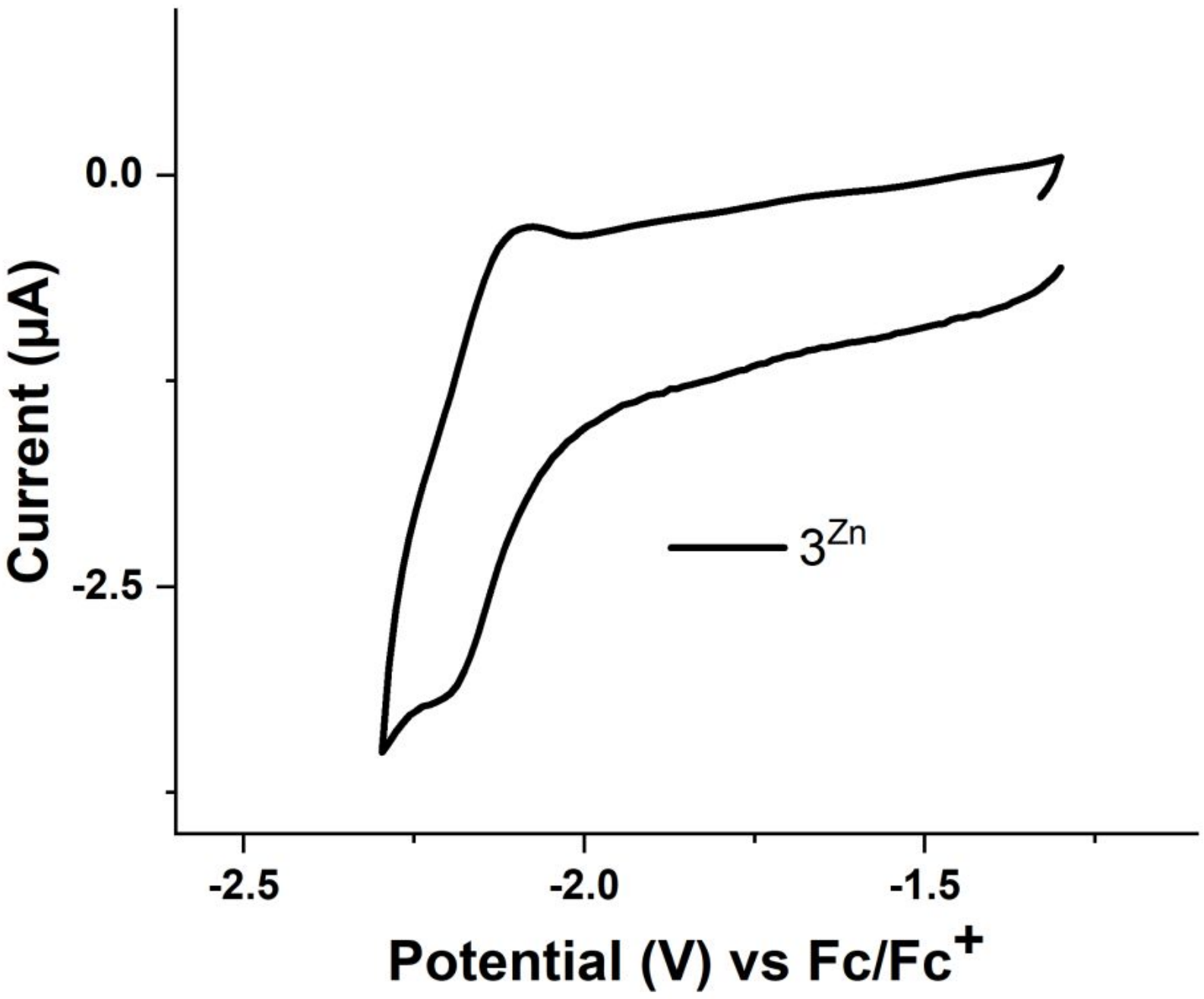

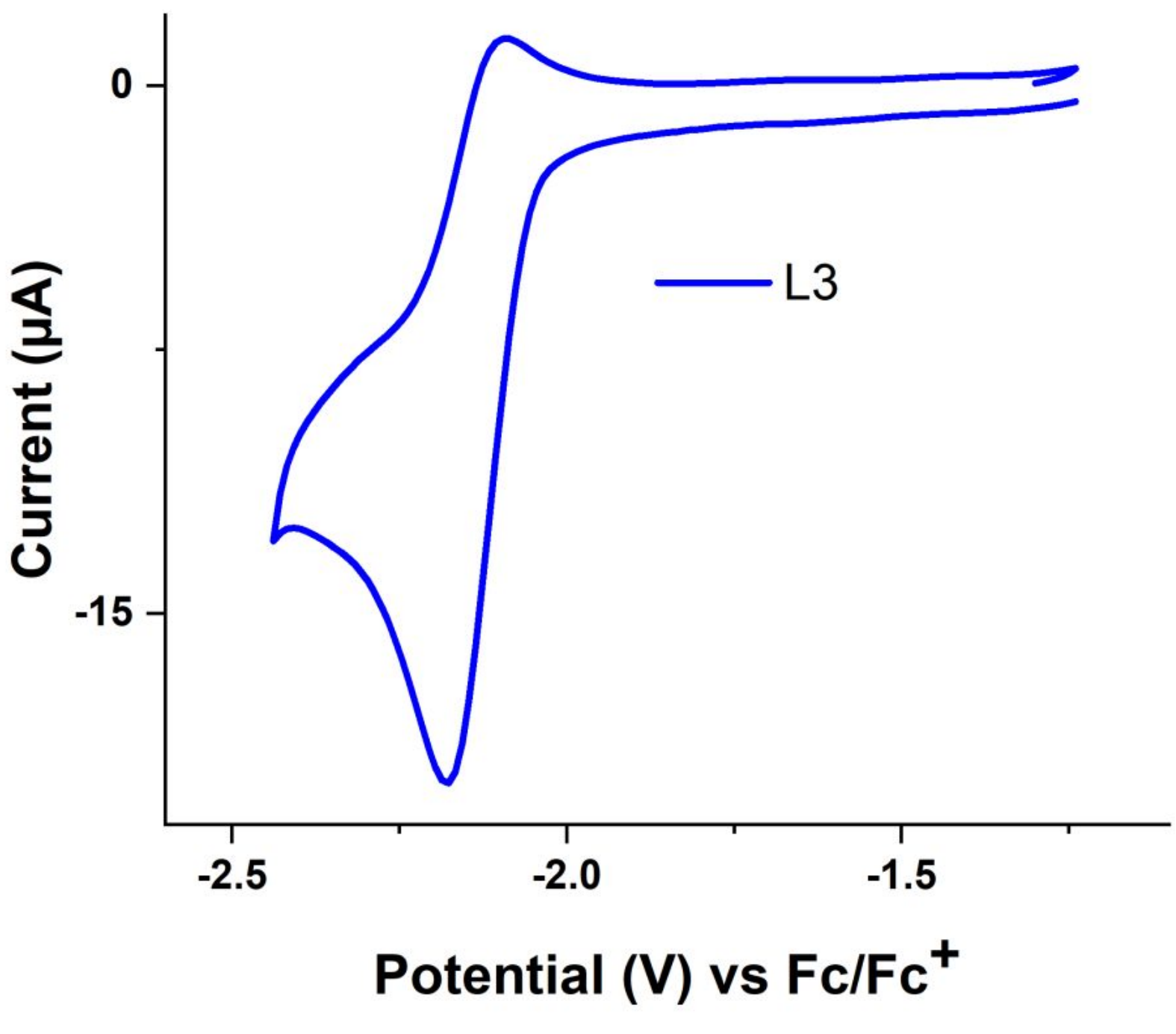

Figure S20. Cyclic voltammograms of complex $\mathbf{3^{Zn}}$ (0.3 mM, black) and Ligand **L3** (1 mM, blue) in DMF, all recorded using 0.1 M $TBAPF_6$ as the supporting electrolyte. Scan rate: 0.1 Vs^{-1}.

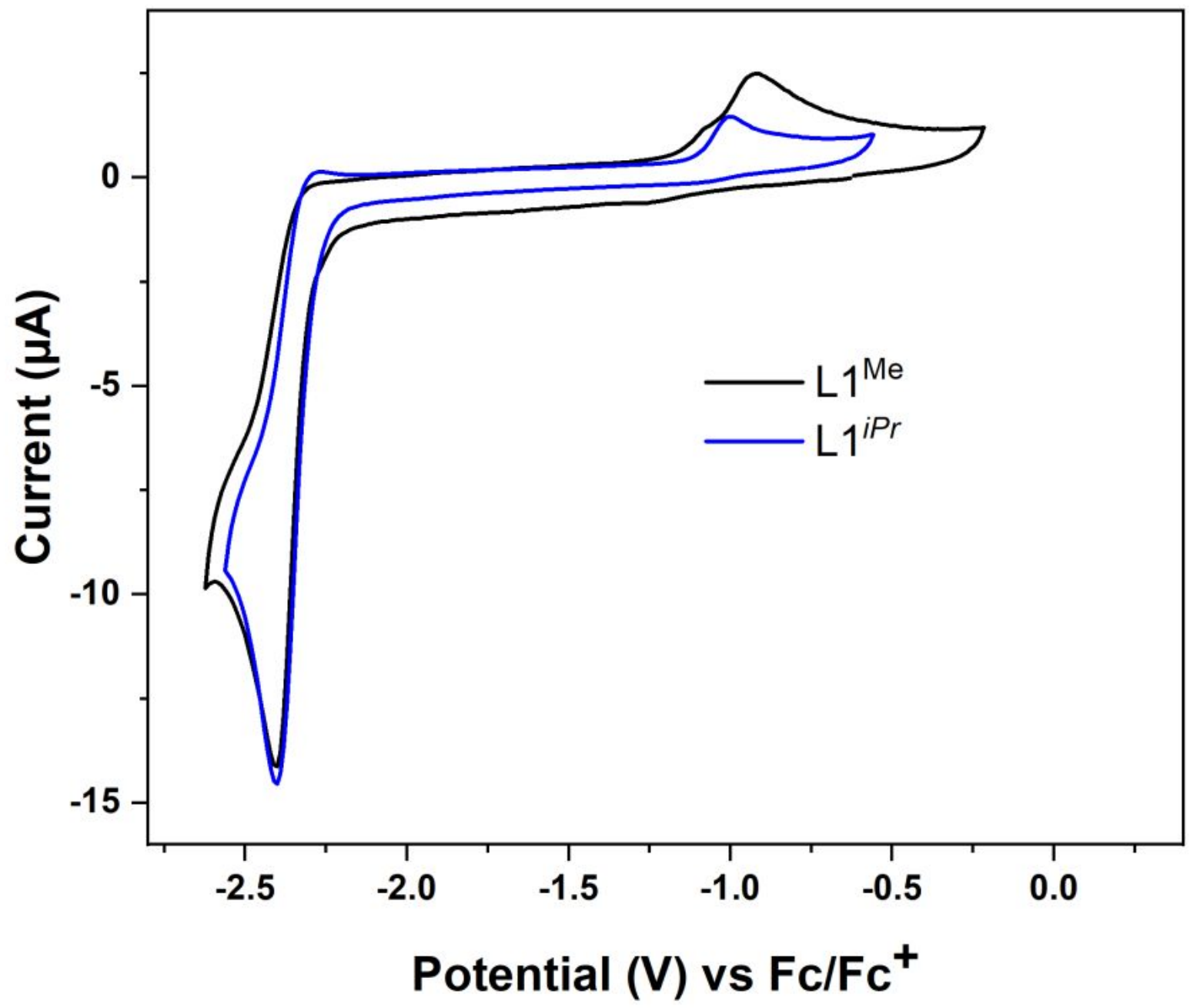

Figure S21. Cyclic voltammograms of ligands $\mathbf{L1^{Me}}$ (black) and $\mathbf{L1^{iPr}}$ (blue) 1 mM respectively in DMF, all recorded using 0.1 M $TBAPF_6$ as the supporting electrolyte. Scan rate: 0.1 Vs^{-1}.

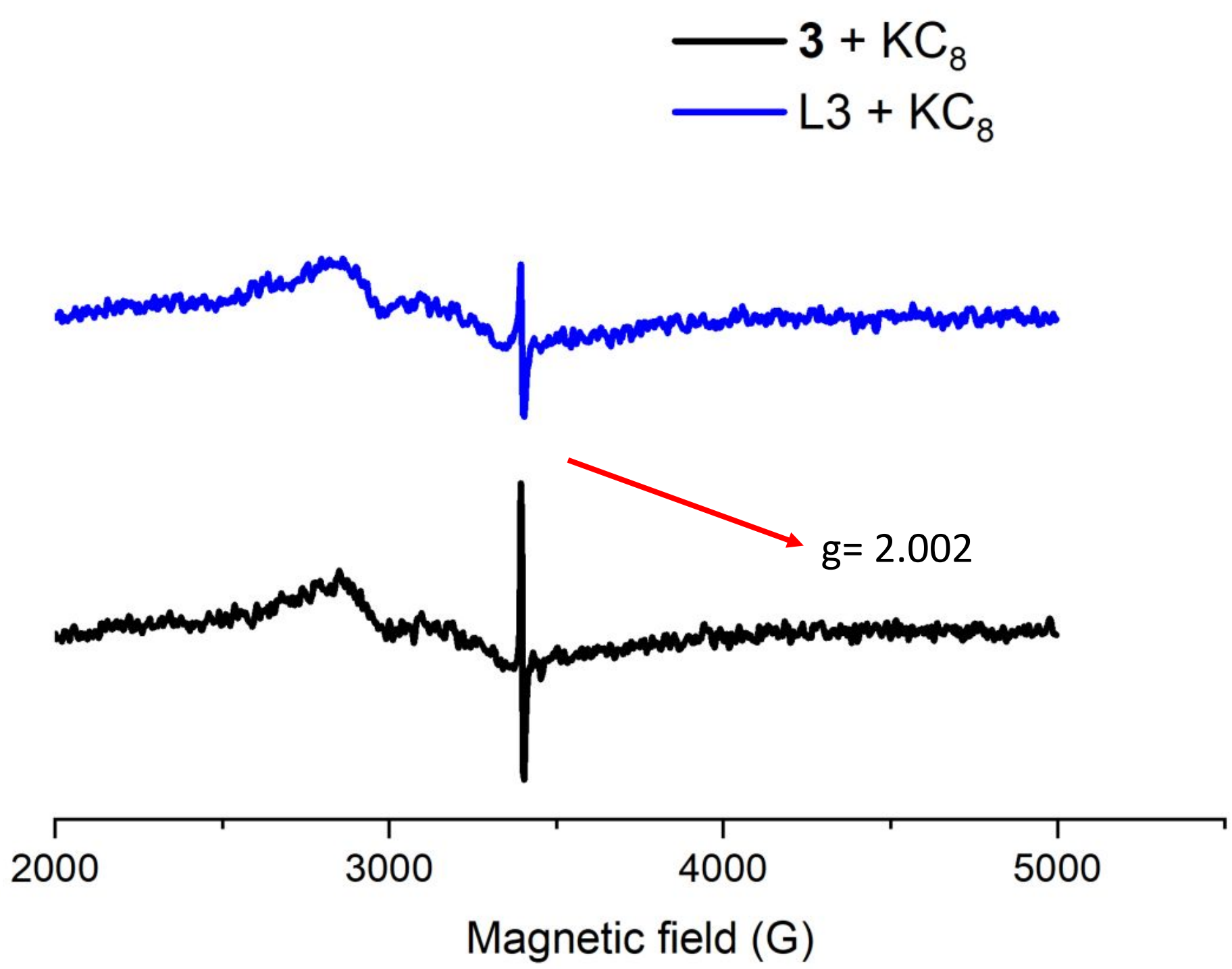

Figure S22. EPR spectra of **3** (1 mM, black) and **L3** (1.5 mM, blue) after reduction with KC_8, showing a signal at g = 2.002. Spectra were recorded at 77 K in DMF and toluene, respectively, with a microwave frequency of 9.4942 GHz.

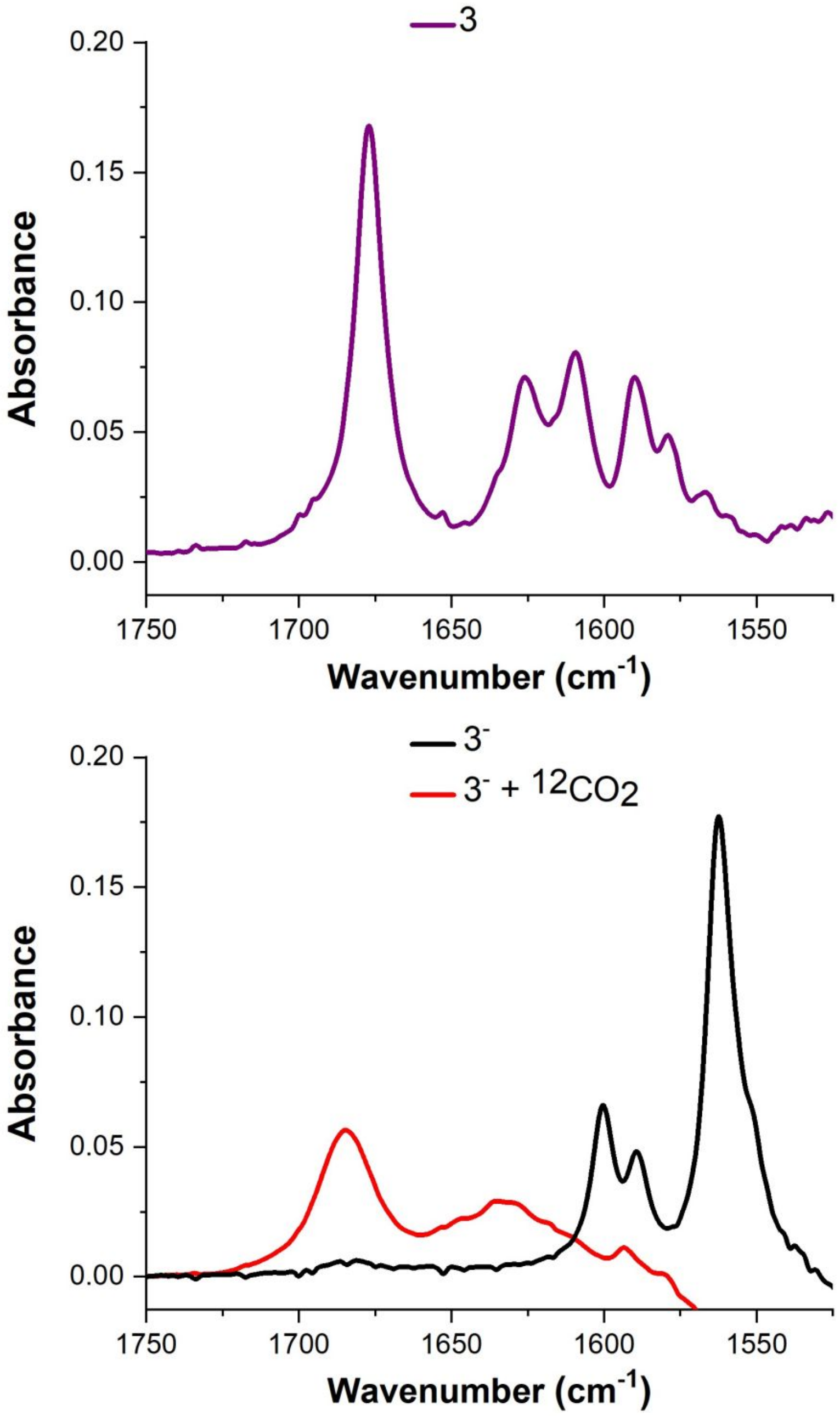

Figure S23. Infrared spectra of complex **3** in acetonitrile: **3** (purple); reduced form of $\mathbf{3}^{-}$ generated by reacting **3** with $Co(Cp^*)_2$ under N_2 (black), $\mathbf{3}^{-}$ under natural-abundance CO_2 (red).

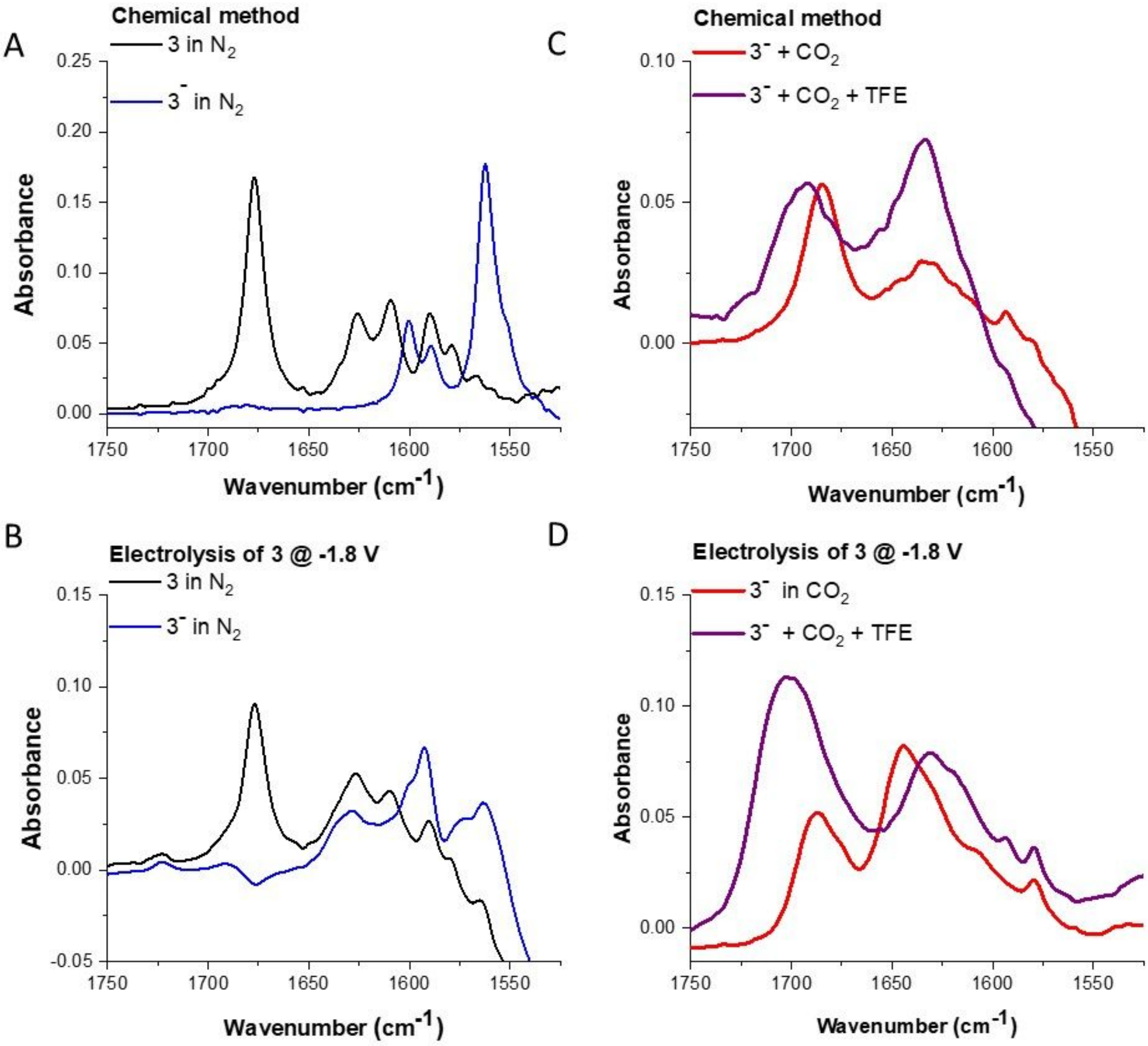

Figure S24. Comparative infrared spectra of chemically generated intermediates of complex **3** and corresponding *in-situ* infrared spectroelectrochemical measurements in ACN. A) chemically generated species in N_2 showing complex **3** (black); **3**$^-$ (blue) B) Infrared spectroelectrochemical spectra of complex **3** under N_2 atmosphere at an applied potential of −1.8 V vs Fc/Fc$^+$, showing spectra of **3** (black) and reduced **3**$^-$ (blue). C) Infrared spectra of chemically generated **3**$^-$ under CO_2 (red) and in the presence of CO_2 with 10 equivalents of TFE (purple). D) Infrared spectroelectrochemical spectra of complex **3** at an applied potential of −1.8 V vs Fc/Fc$^+$, in the presence of CO_2 (red) and CO_2 with 5 equivalents of TFE (purple), respectively.

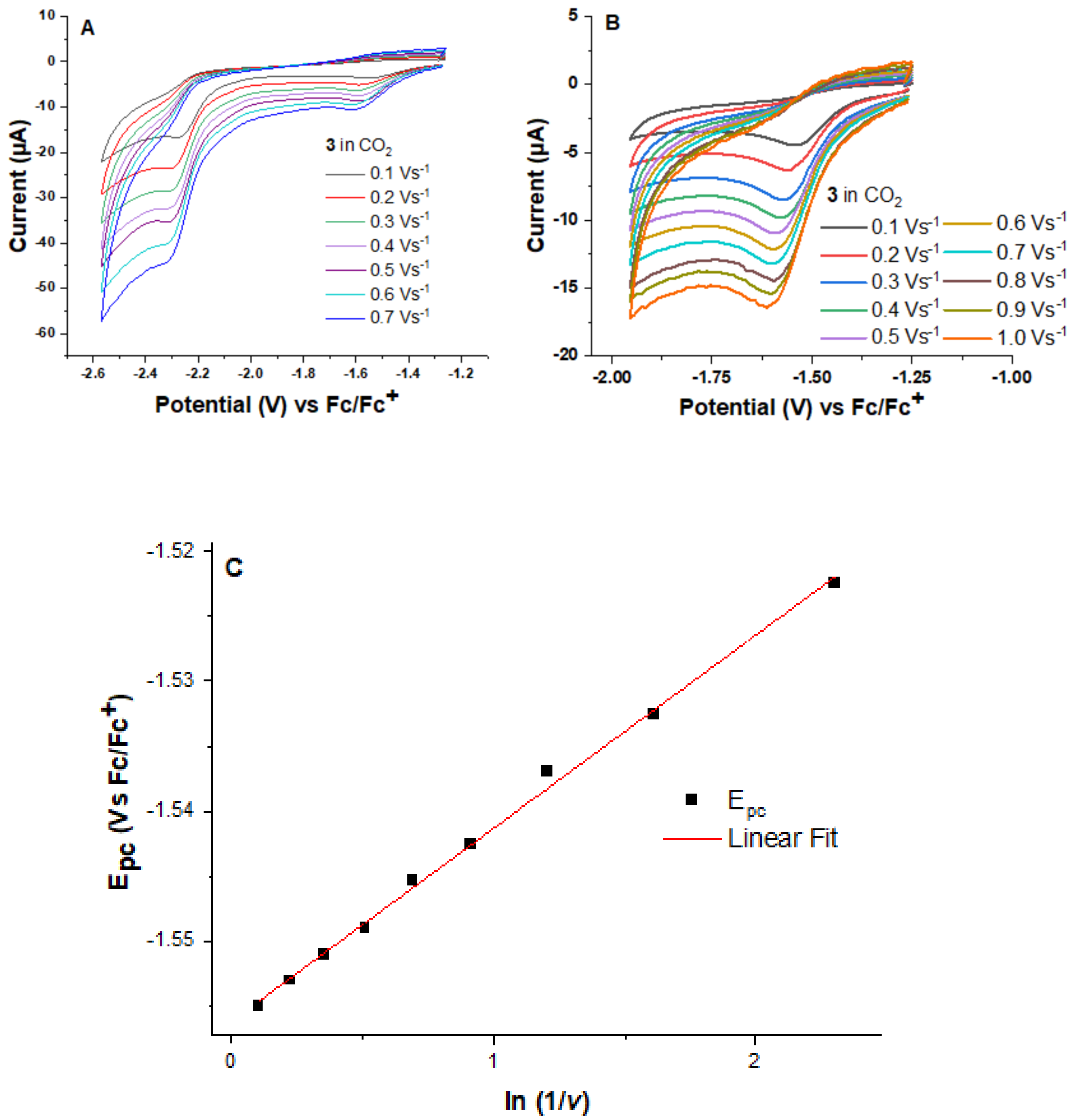

Figure S25. The cyclic voltammograms of **3** (1 mM in DMF) (A & B) showing the CO_2 binding at −1.53 V in 0.1 M $TBAPF_6$/DMF in saturated CO_2 solution. C) Plots of E_{pc} against ln (1/v) following the equation 1 in the main manuscript.

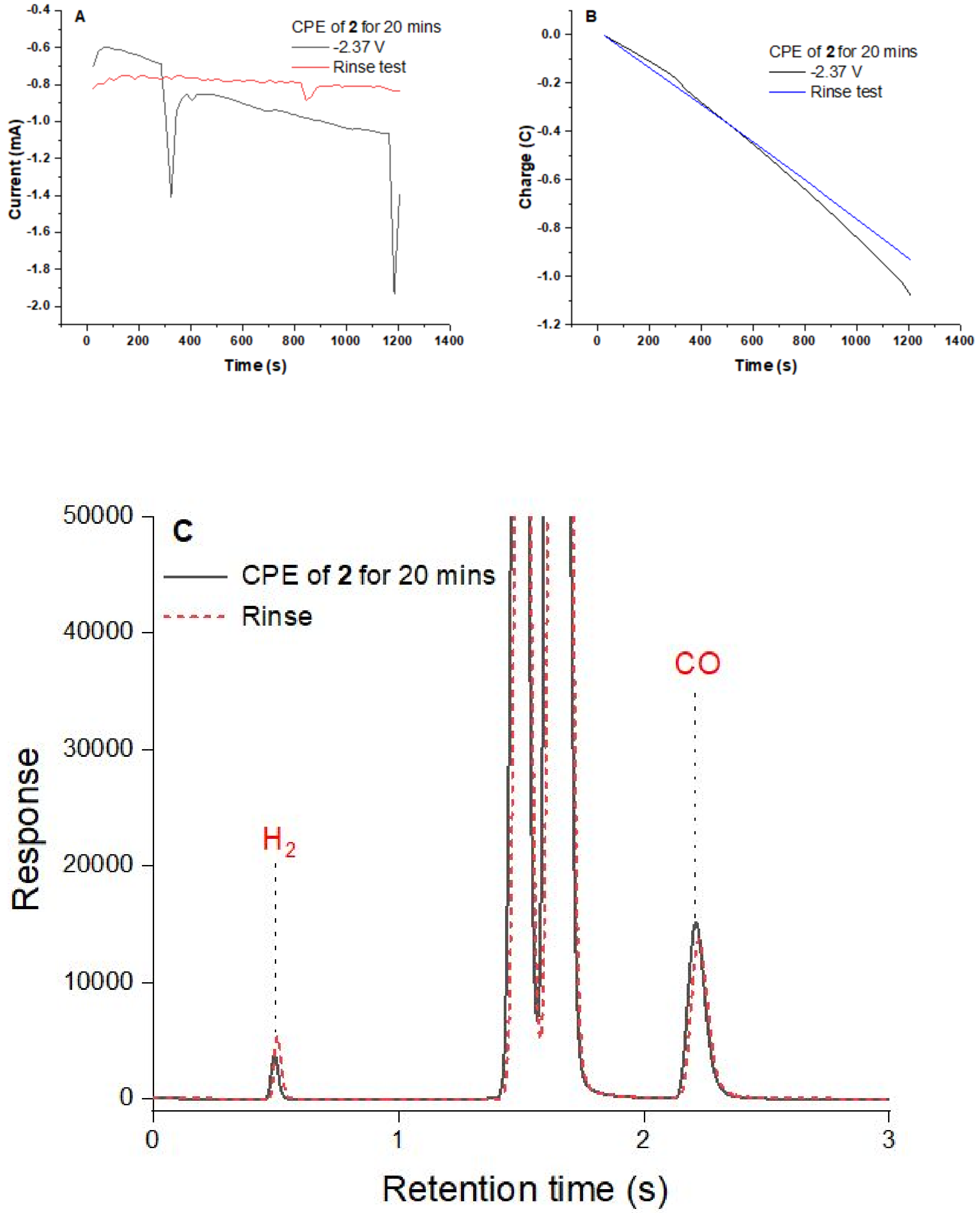

Figure S26. Bulk electrolysis of complex **2** under CO_2 atmosphere. A) Current vs. time plots recorded for control potential electrolysis; B) corresponding charge vs. time plot; C) gas chromatograph of headspace products following electrolysis at –2.37 V for 20 mins.

A

B

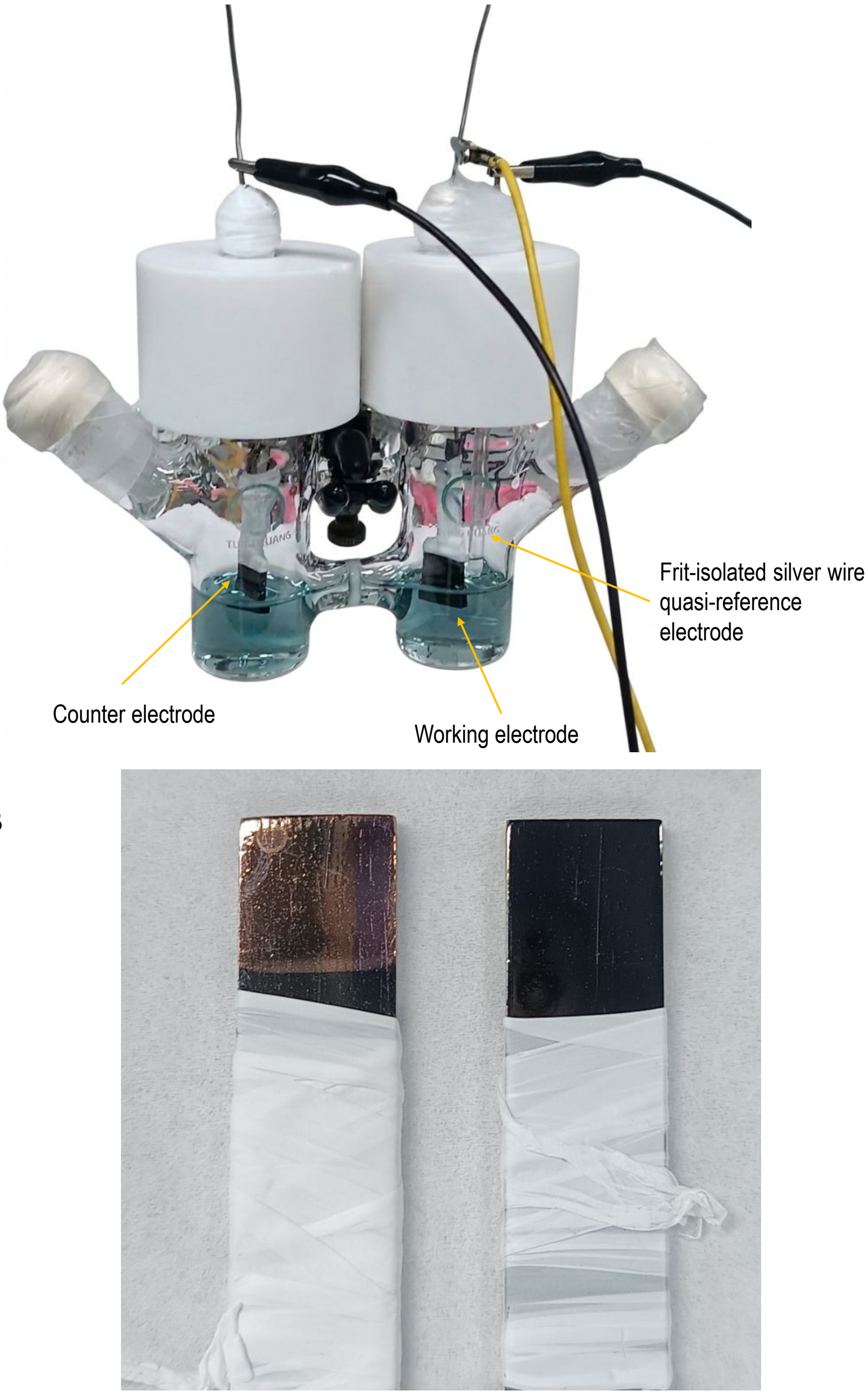

Figure S27. A) A photograph of the two-compartment H-cell used for controlled potential electrolysis of the copper complexes studied here, illustrating the positions of the working electrode, counter electrode, and frit-isolated silver wire quasi-reference electrode. B) Photograph of the glassy carbon electrode after 20 min bulk electrolysis of complex **2** (left) and complex **3** (Right) in DMF.

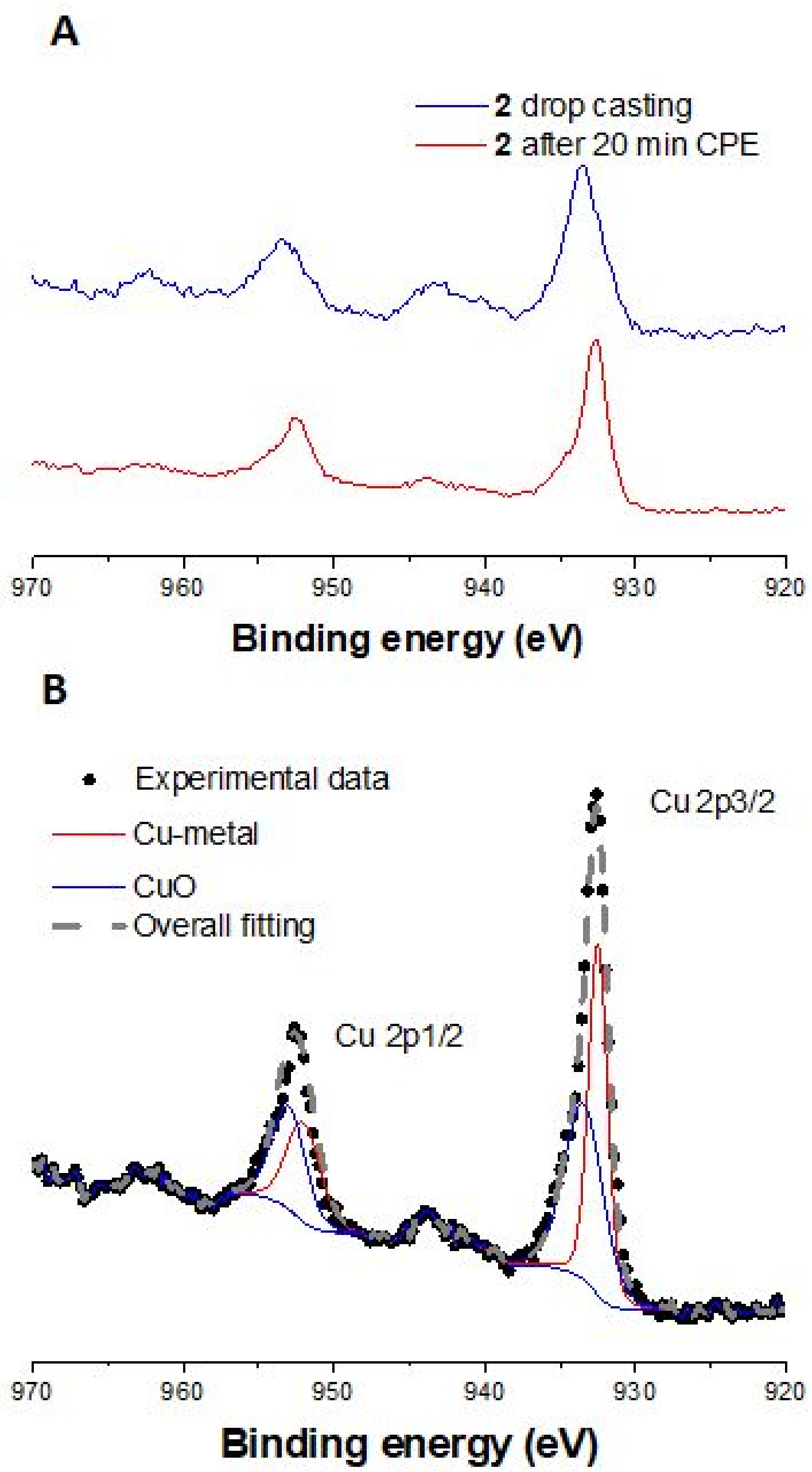

Figure S28. XPS analysis of the Cu 2p region for complex **2**. A) Comparison of the Cu 2p XPS spectra of drop-cast complex **2** (blue) and the glassy carbon electrode after 20 mins of controlled potential electrolysis (red). B) Deconvoluted Cu 2p spectrum of the post-electrolysis electrode. The fitting of the Cu(II) component was based on the reference spectrum of drop-cast complex **2**, while the Cu(0) and CuO components were fitted using literature reference spectra.[24]

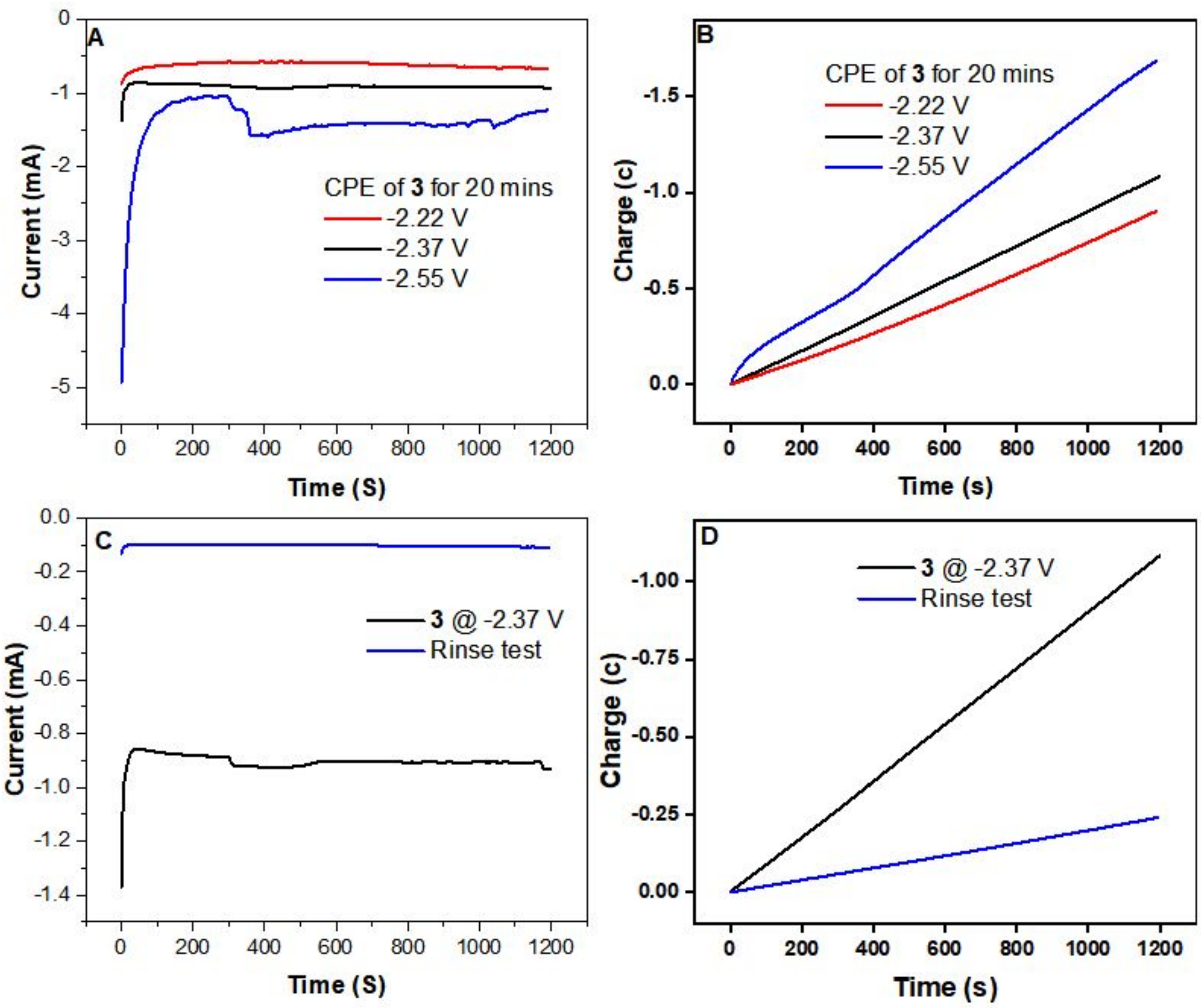

Figure S29. Bulk electrolysis of Complex **3** under CO_2 atmosphere. A) Current vs. time plots at variable potentials; B) Corresponding charge passed vs. time plots at variable applied potential values; C) Current vs. time plots at –2.37 V and its comparison with the rinse test; D) Corresponding charge passed vs. time at –2.37 V and its comparison with the rinse test.

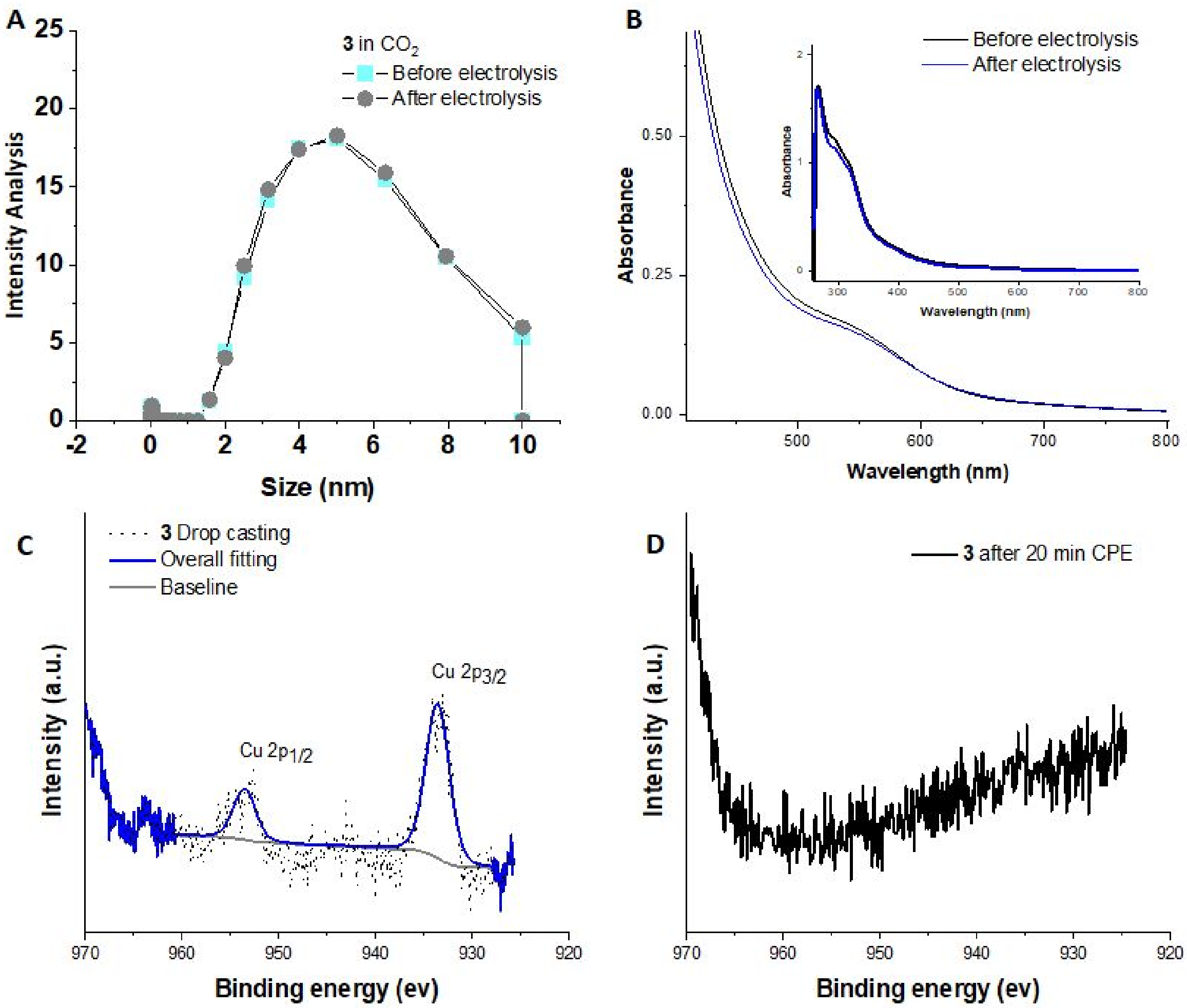

Figure S30. A) Dynamic light scattering measurement of **3** before and after the CPE experiment for 20 mins. B) UV-Vis spectra of complex **3** (0.5 mM) in DMF containing 0.1 M $TBAPF_6$ recorded before (black) and after (blue) 20 min of controlled potential electrolysis. Inset**:** UV-Vis spectra of equally diluted solutions of complex **3** recorded before (black) and after (blue) electrolysis. (C & D) XPS analysis in Cu 2p region of **3**. C) Cu 2p XPS spectra of drop-cast complex **3** and D) the glassy carbon electrode after 20 mins of controlled potential electrolysis.

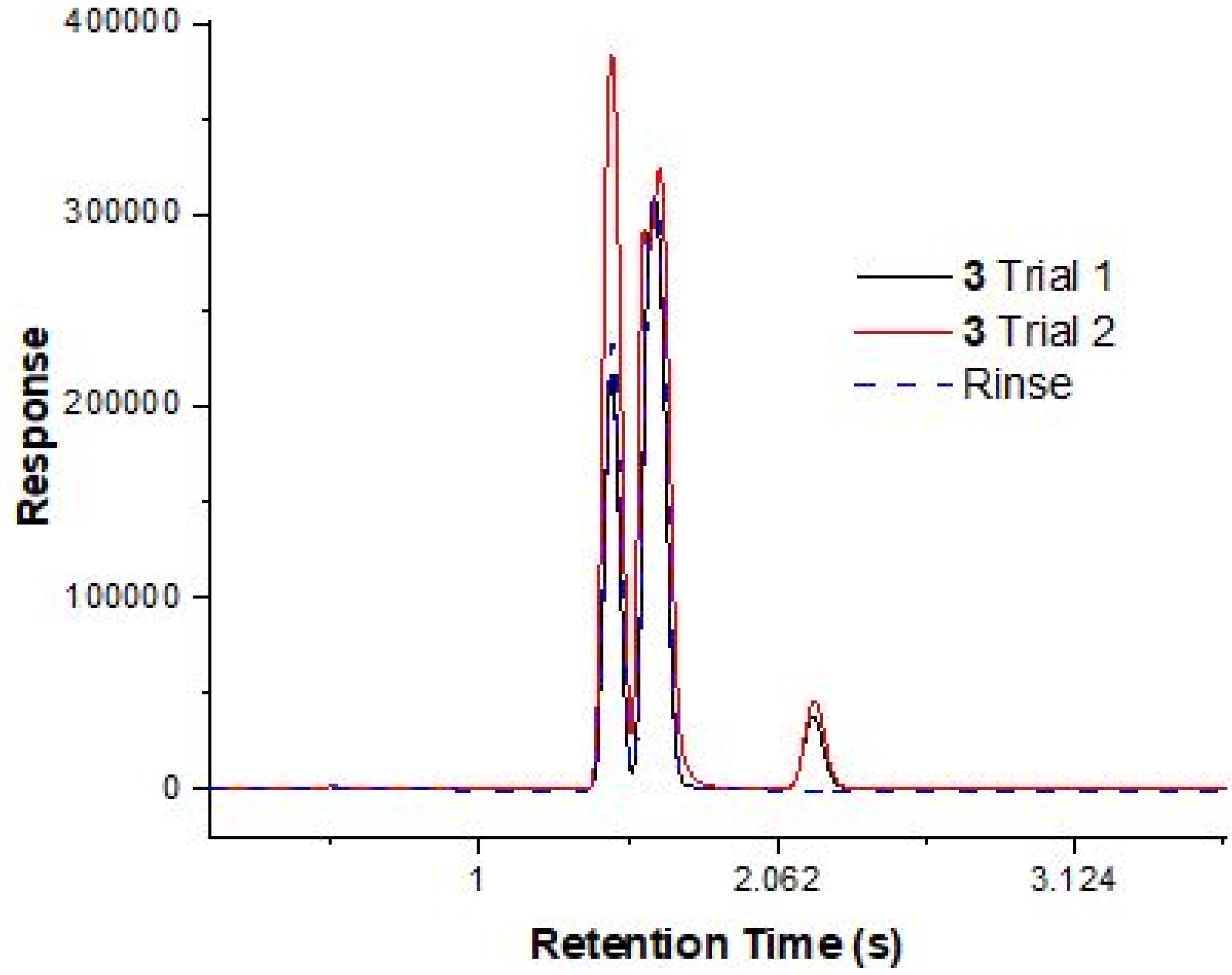

Figure S31. The blue dashed line indicates the GC chromatograph from post-electrolysis of the GC electrode that had been used for complex **3** for 20 minutes of electrolysis.

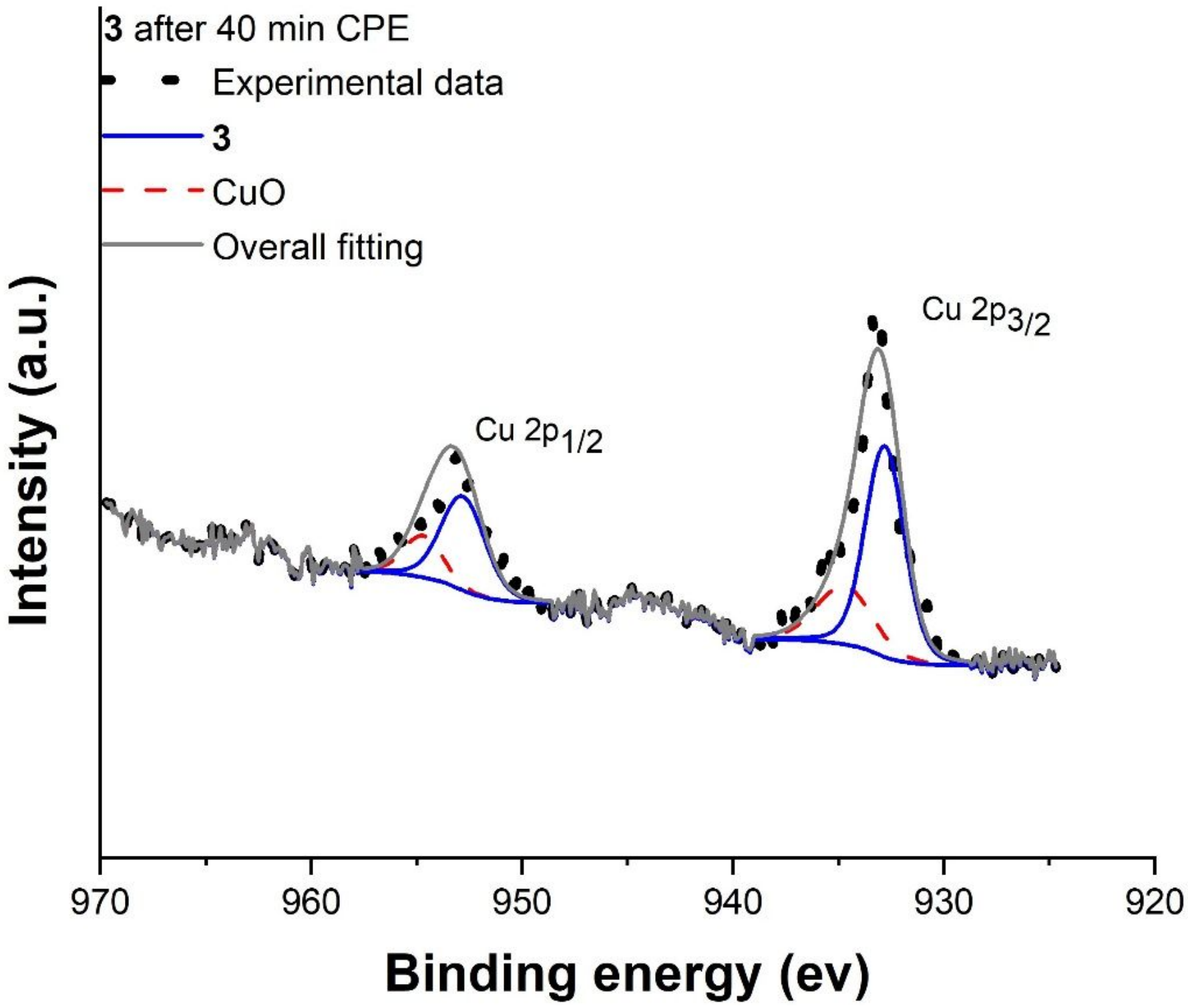

Figure S32. XPS analysis in Cu 2p region of **3** after 40 mins of electrolysis.

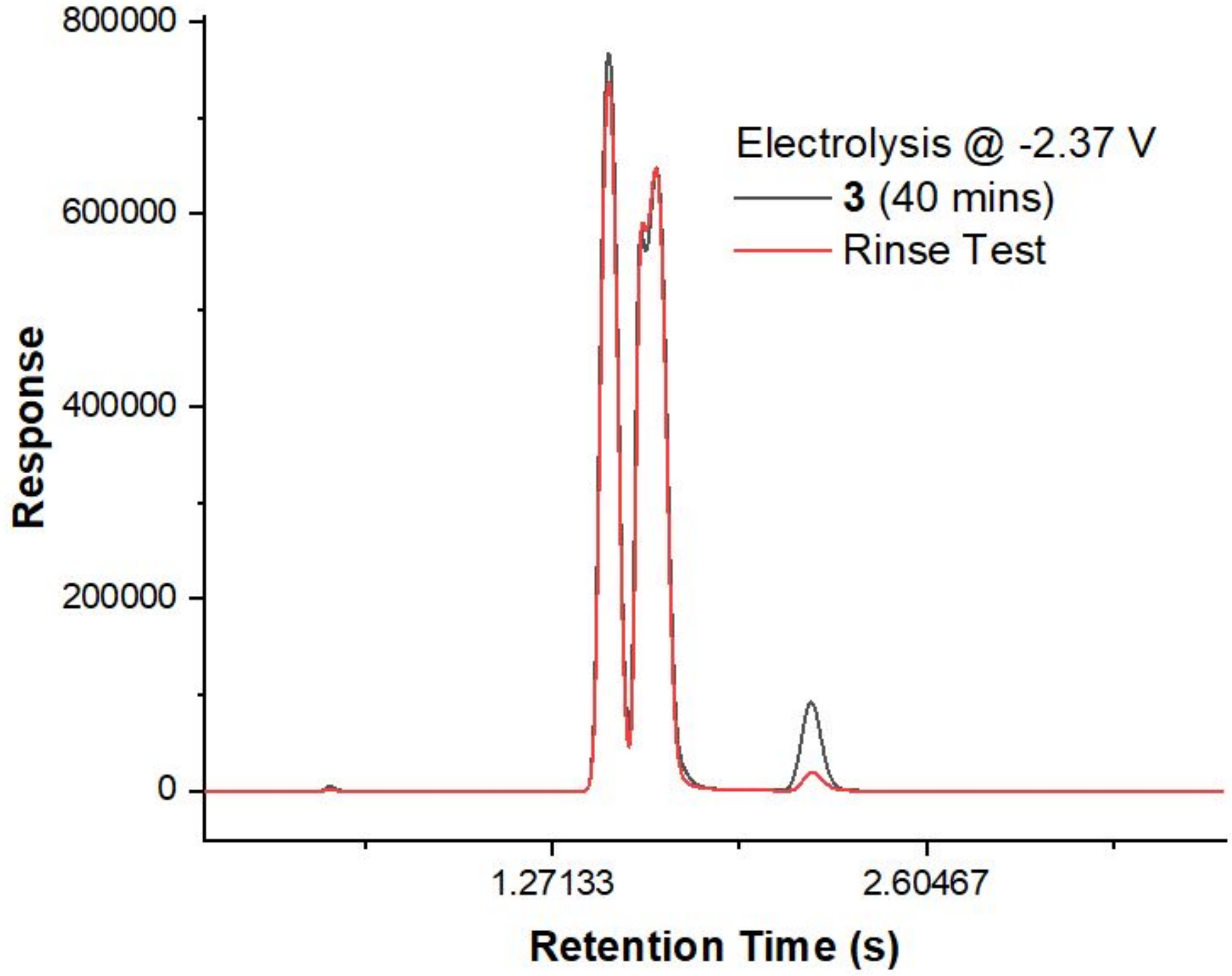

Figure S33. GC-chromatograph. The red GC chromatograph refers to post-electrolysis of the GC electrode used for complex **3** for 40-minute electrolysis.

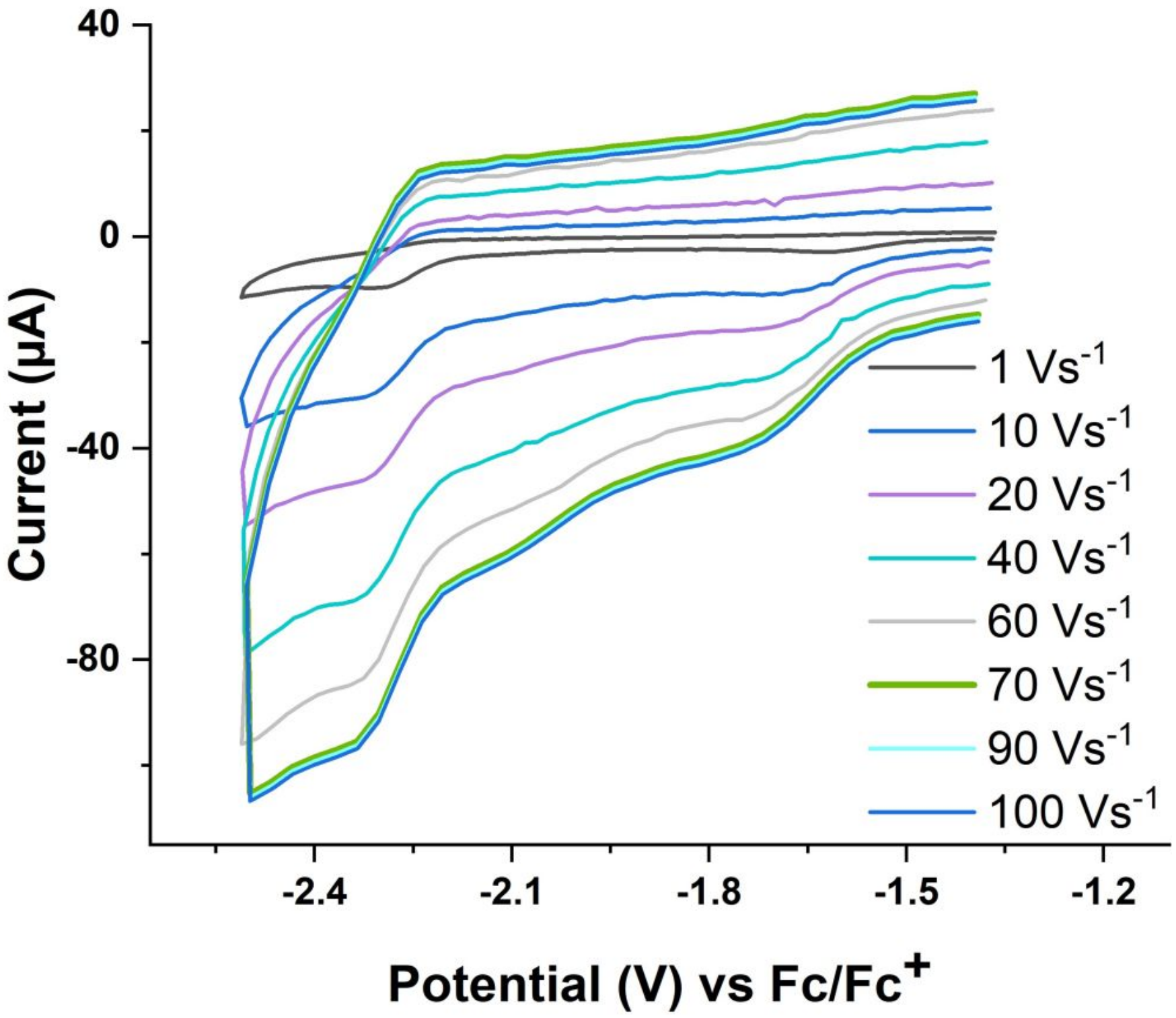

Figure S34. Cyclic voltammograms of complex **3** (1 mM) recorded in the presence of CO_2 with different scan rates at 75 equivalents of TFE.

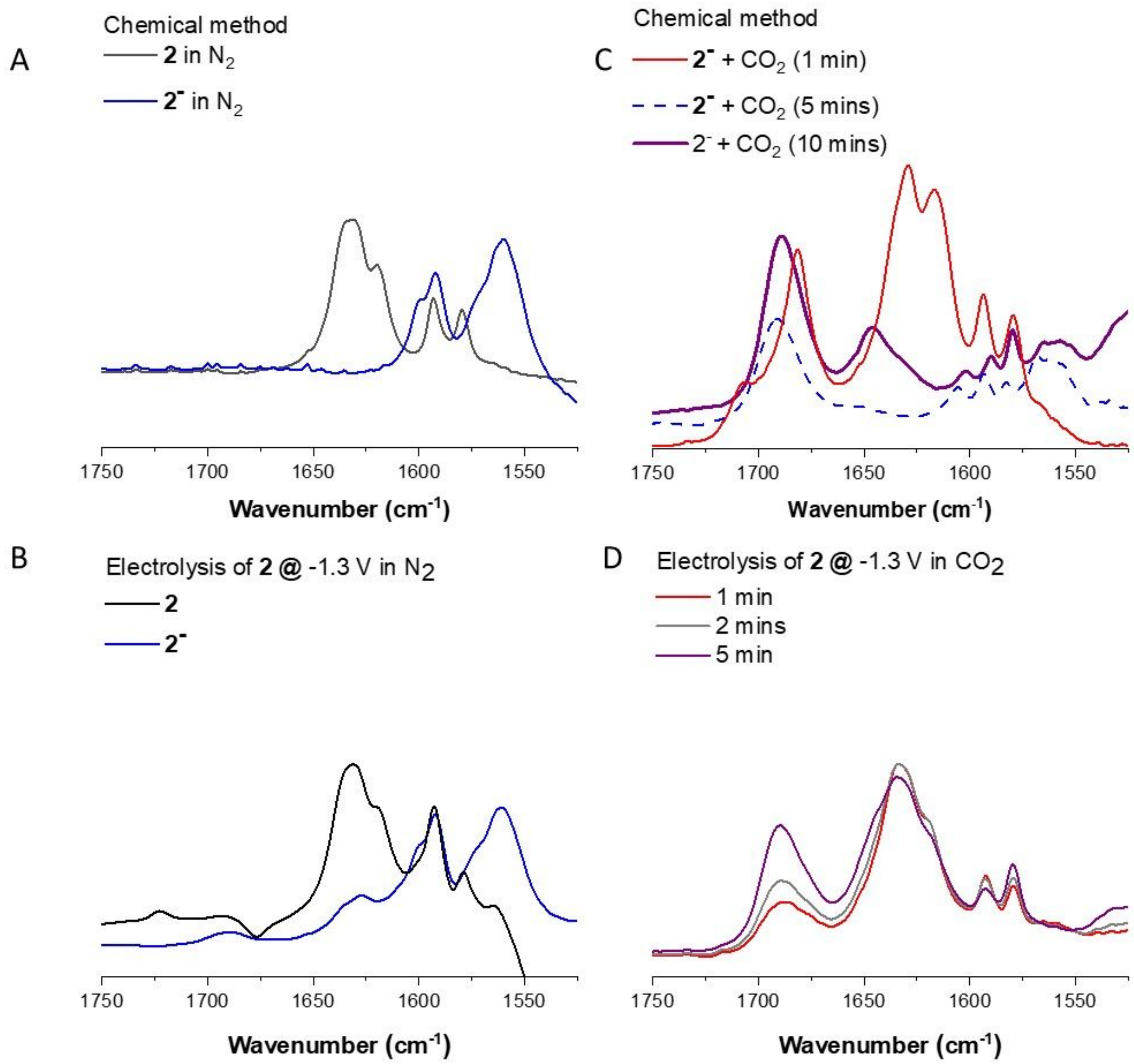

Figure S35. Comparative infrared spectra of chemically generated intermediates of complex **2** and corresponding *in-situ* infrared spectroelectrochemical measurements in ACN. A) chemically generated species in N_2 showing complex **2** (black); **2**$^-$ (blue); B) Infrared spectroelectrochemical spectra of complex **2** under N_2 atmosphere at an applied potential of −1.3 V vs Fc/Fc$^+$, showing spectra of **2** (black) and reduced **2**$^-$ (blue). C) Infrared spectra of chemically generated **2**$^-$ under CO_2 recorded over different time intervals after mixing CO_2 with the solution containing **2**$^-$: 1 min (red), 5 mins (blue dots) and 10 mins (purple). D) Infrared spectroelectrochemical spectra of complex **2** at an applied potential of −1.3 V vs Fc/Fc$^+$, under CO_2 atmosphere recorded after 1 min (red), 2 mins (gray) and 5 mins (purple) respectively.

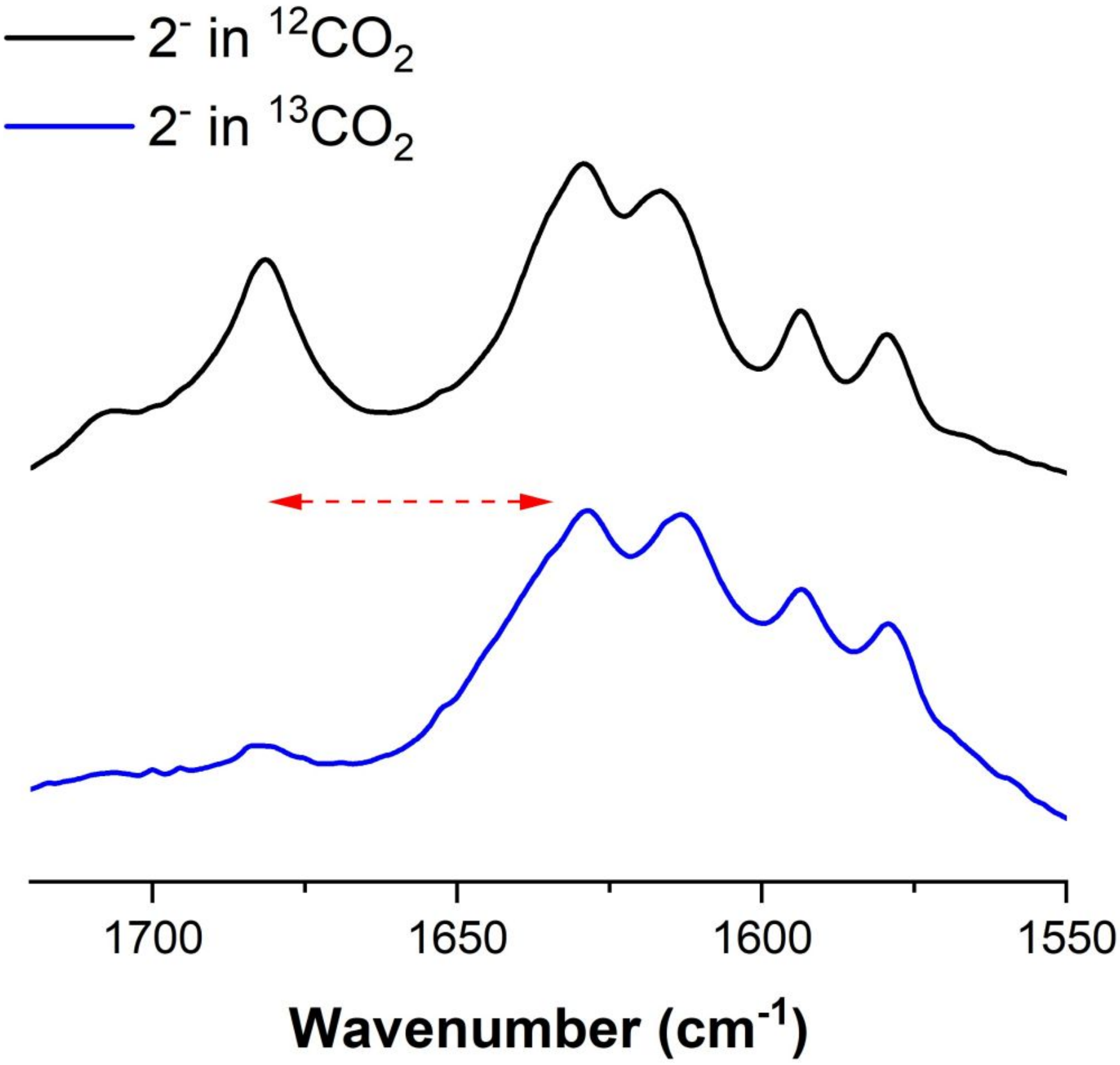

Figure S36. Infrared spectra of reduced complex **2**⁻ generated using 1.2 equivalents Co(Cp*)$_2$ in acetonitrile under natural-abundance $^{12}CO_2$ (black) and $^{13}CO_2$ (99%) (blue).

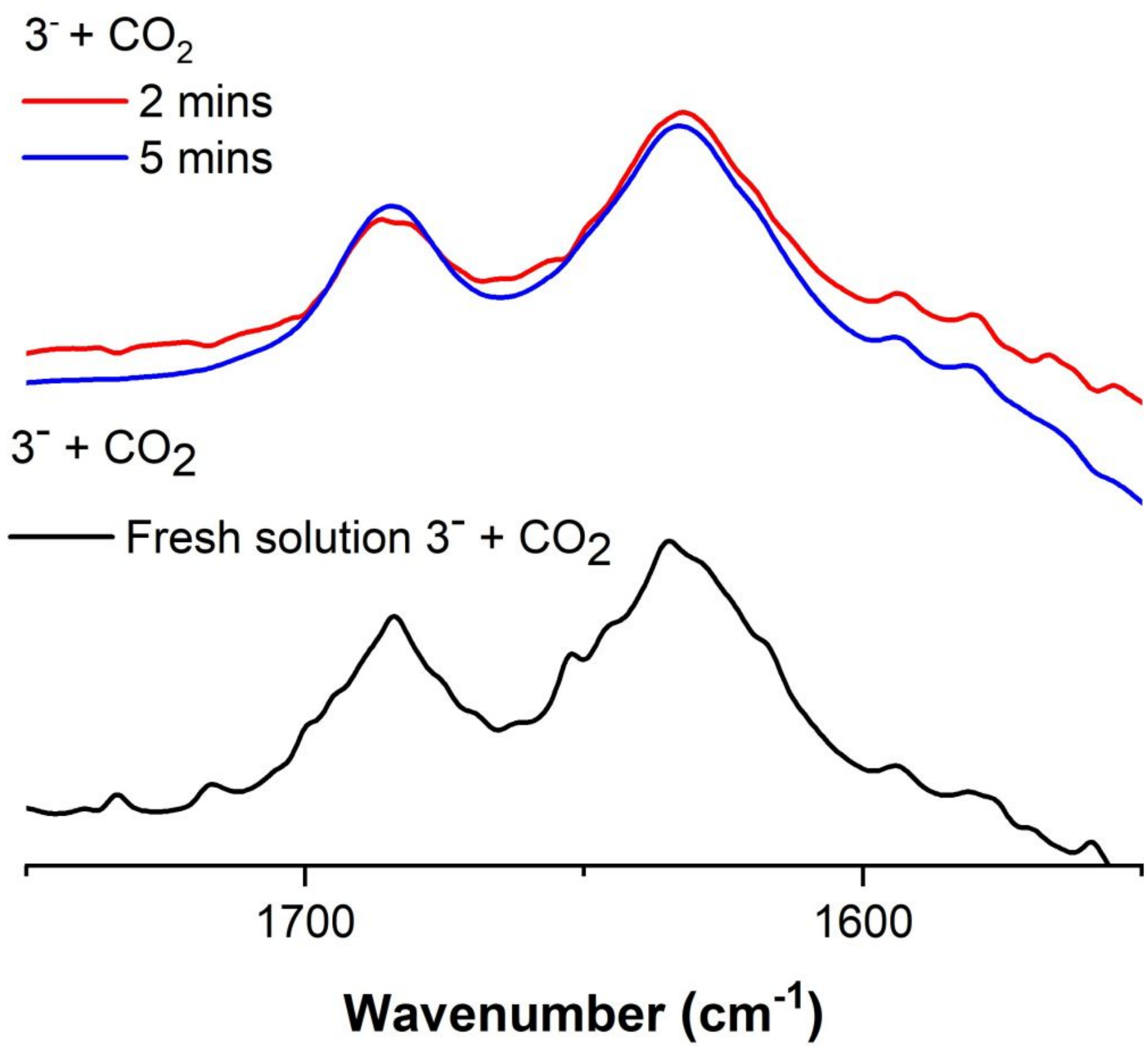

Figure S37. Infrared spectra of **3⁻** in acetonitrile under CO_2 at different time intervals: 2 min (red) and 5 min (blue) after mixing CO_2 with the solution containing **3⁻**. The black trace corresponds to a freshly prepared solution of **3⁻** and CO_2 stored in the glovebox for 10 minutes, showing no significant changes in the characteristic IR peaks.

	1^{Me}	2	3
**SOMO of [Cat]**	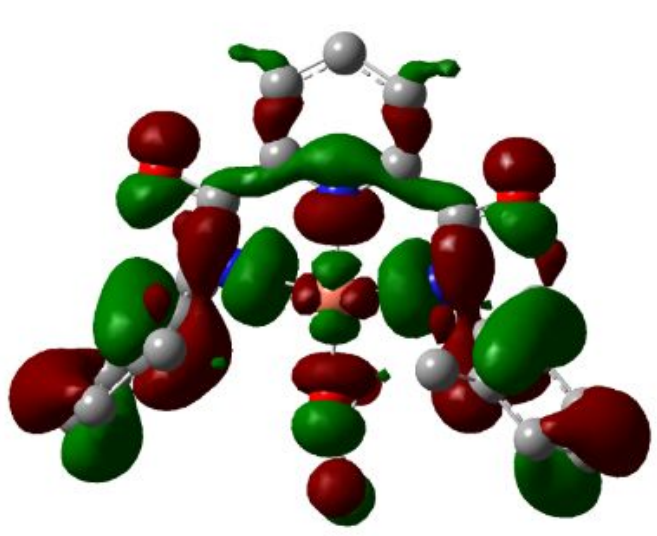	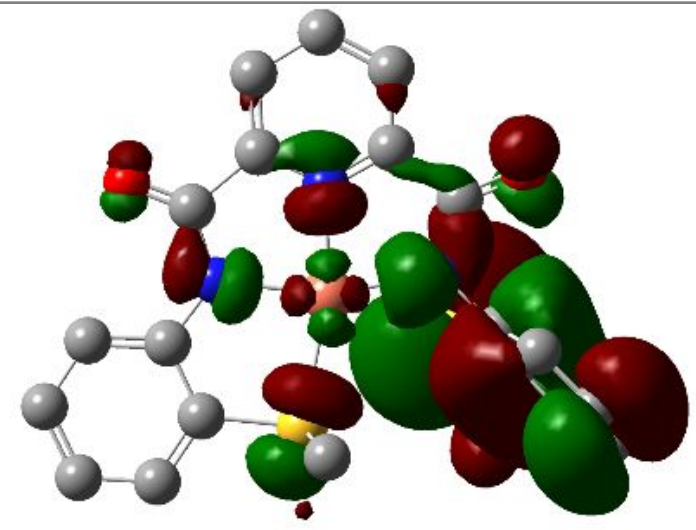	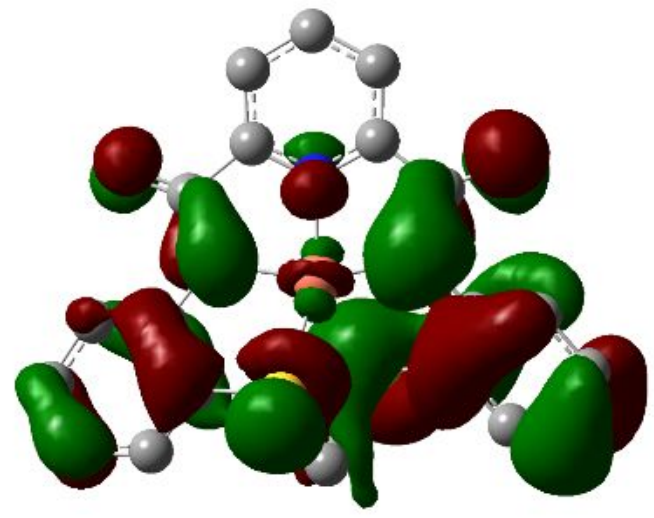
**HOMO of $[Cat]^-$**	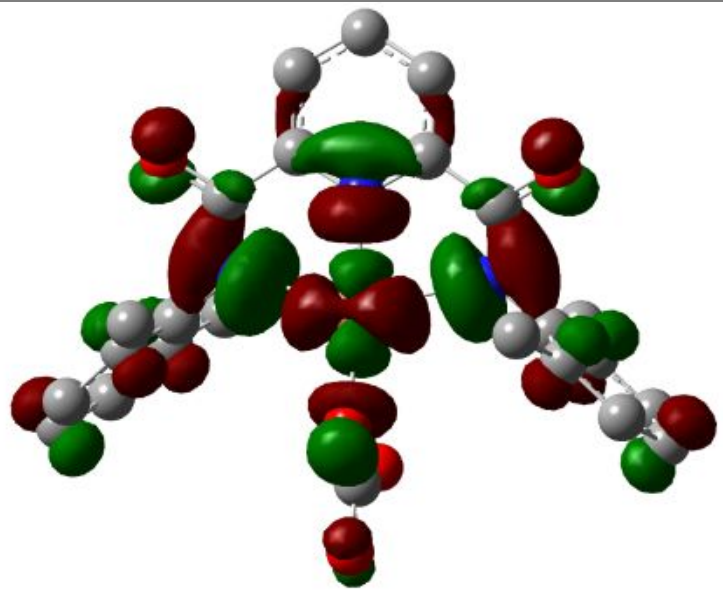	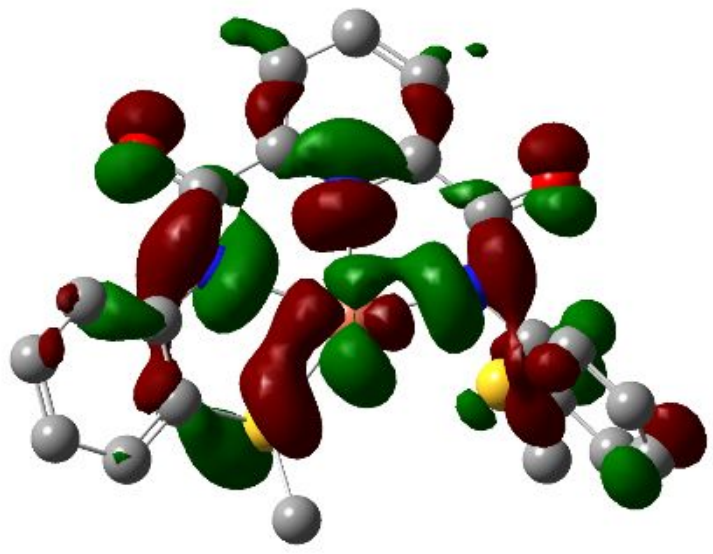	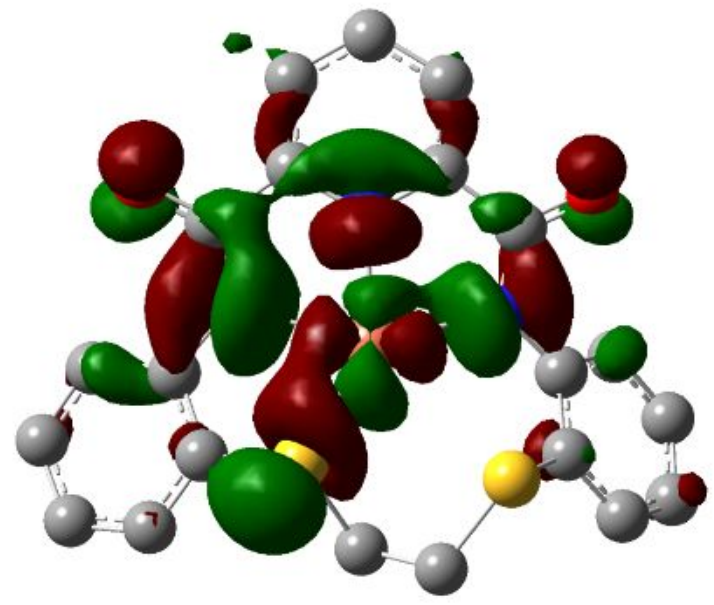
**SOMO of $[Cat]^{2-}$**	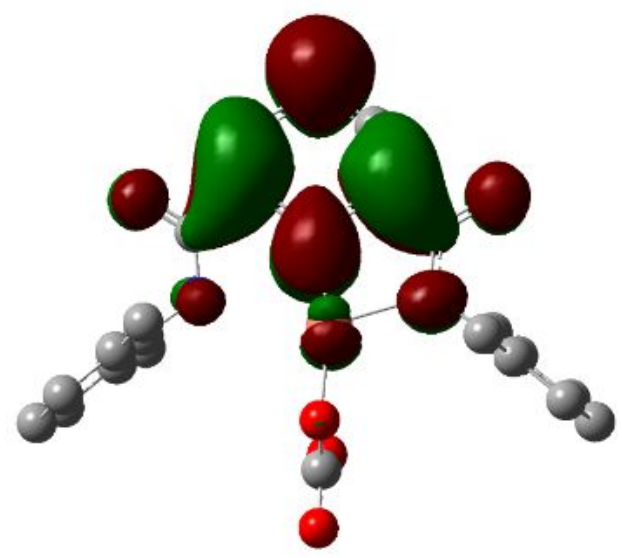	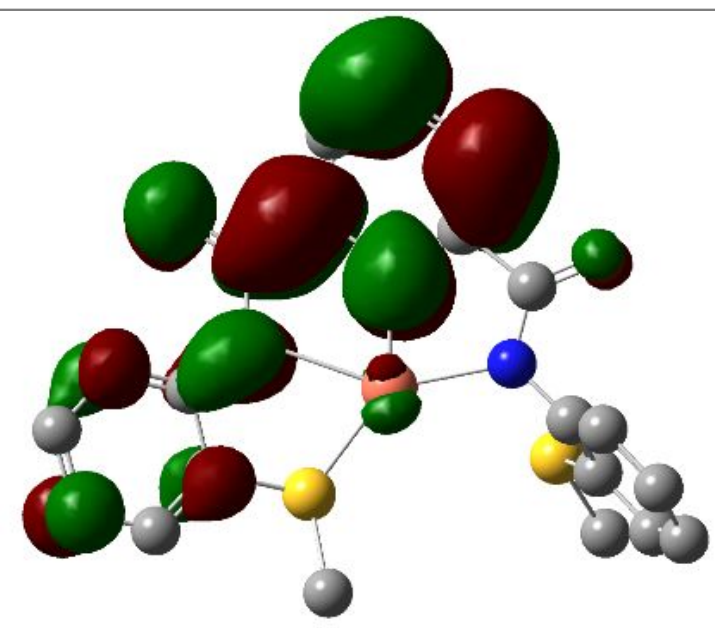	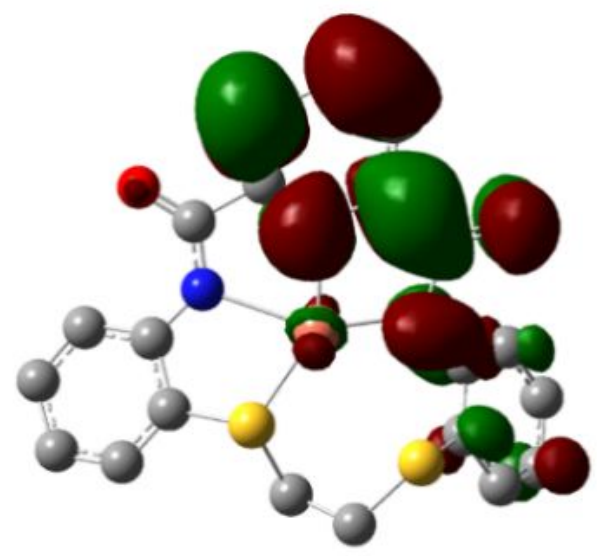

Figure S38. Molecular orbitals of the resting state, one-electron and two-electron reduction of complex $\mathbf{1}^{\mathbf{Me}}$, **2** and **3** obtained from DFT calculations.

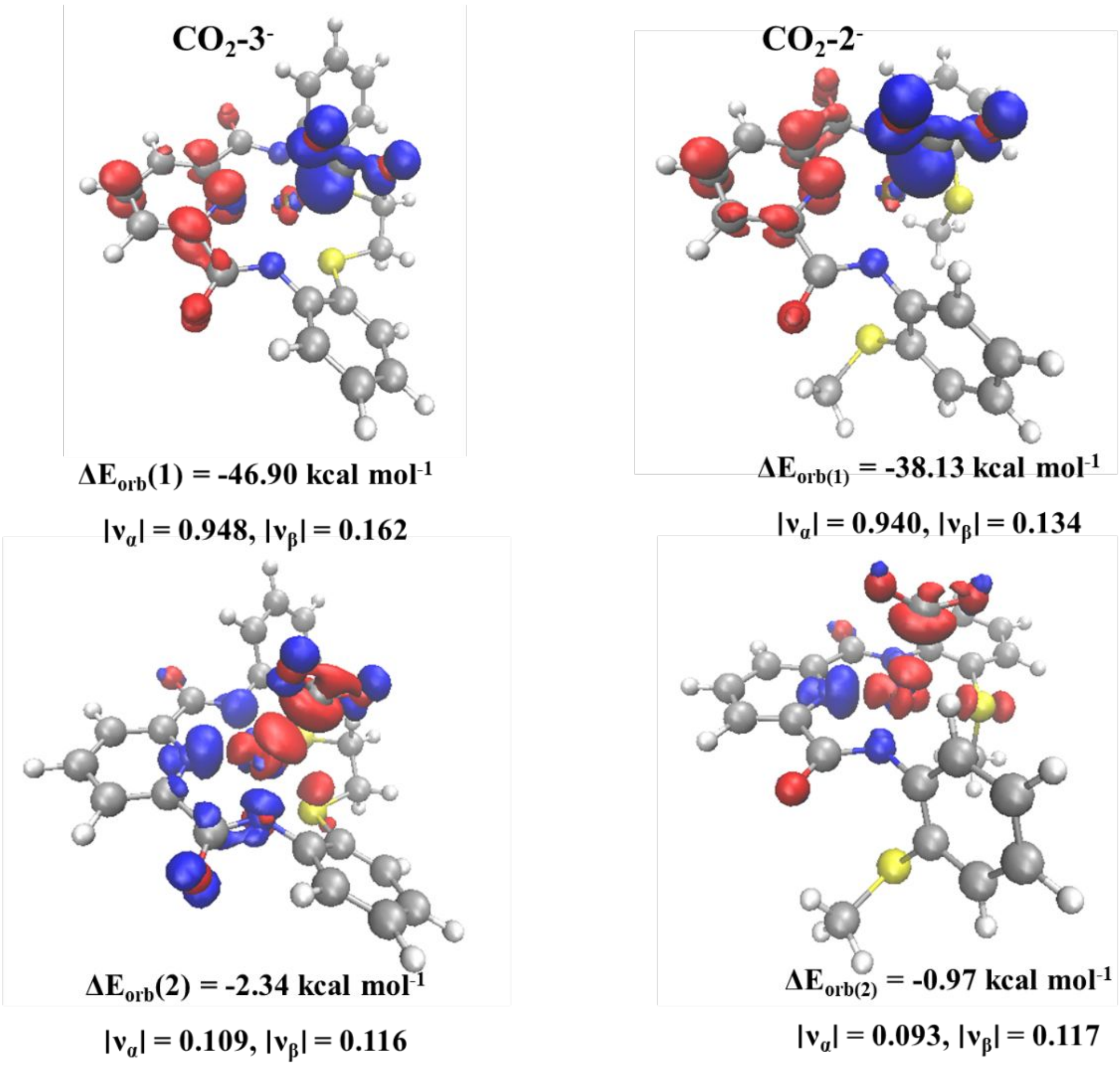

Figure S39. Selected shape of deformation densities for **CO_2-2$^-$** and **CO_2-3$^-$** complex. Shape of the deformation densities $\Delta\rho(1)$-(2), which are associated with the orbital interactions $\Delta E_{orb}(1)$,(2) and eigenvalues |νn| of the charge flow. The isosurface values are 0.005 for $\Delta\rho(1)$, and 0.001 for $\Delta\rho(2)$. The color code of the charge flow is red → blue.

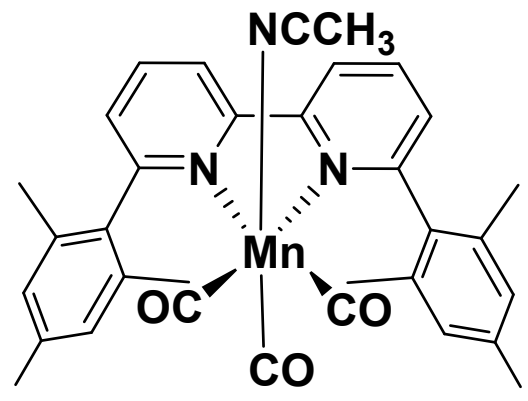

Mn(Mesbpy)

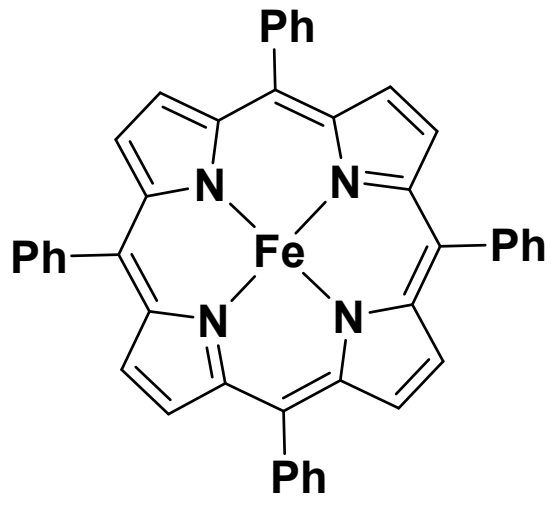

FeTPP

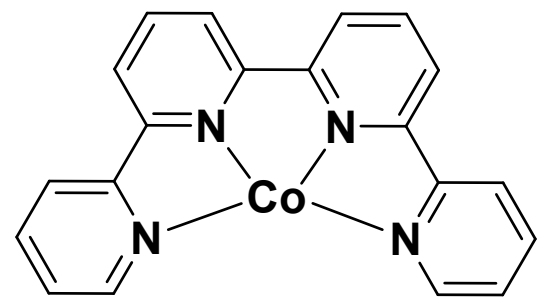

Coqpy

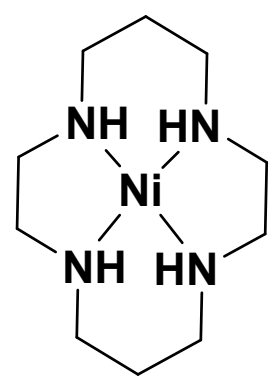

Ni(cyclam)

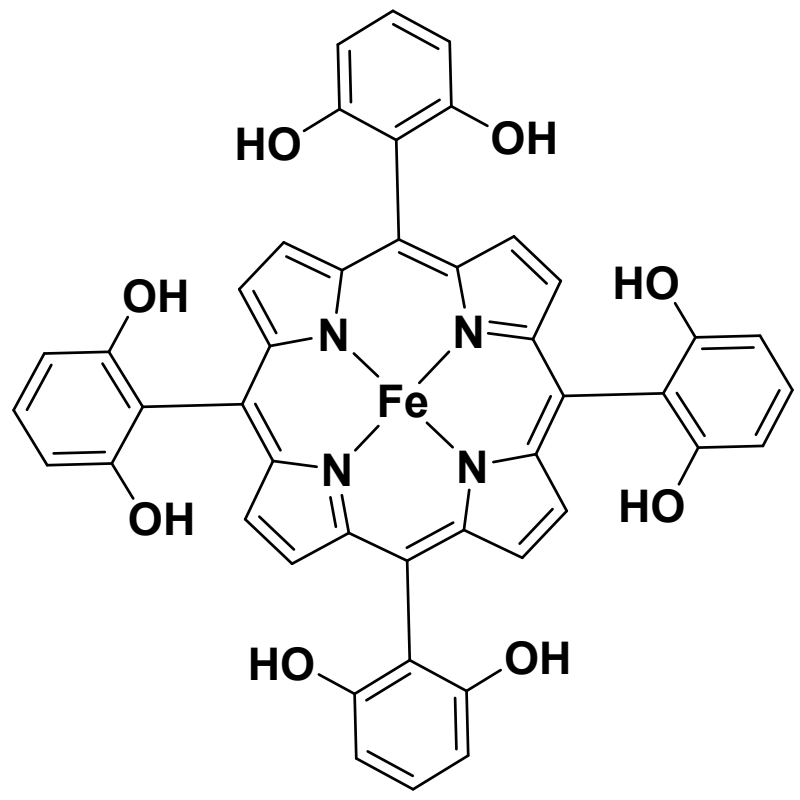

Fe-TDHP

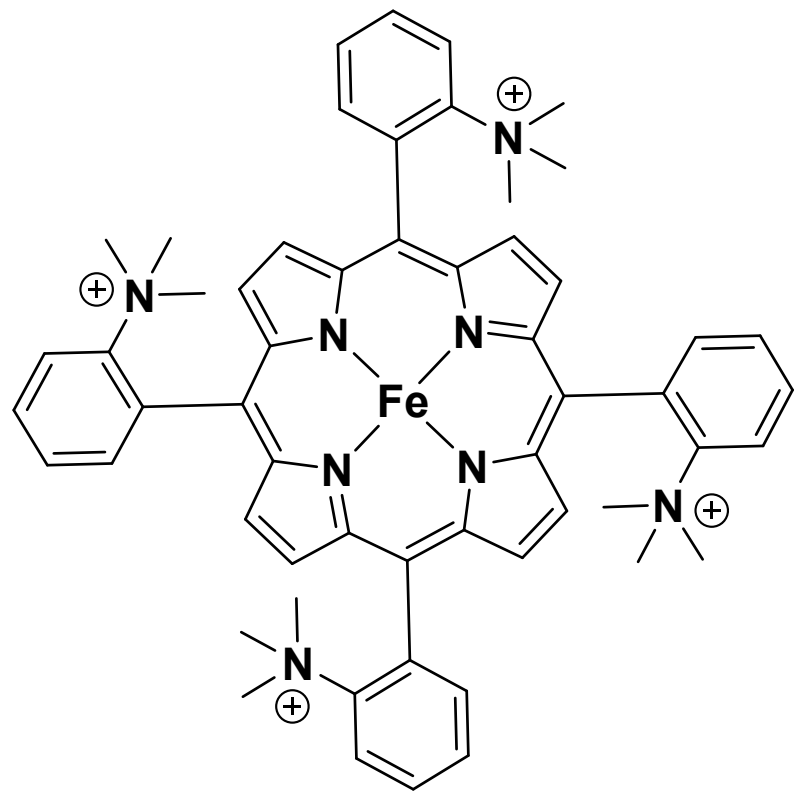

Fe-O-TMA

Figure S40. The molecular structures of the first-row transition metal complexes used for the catalytic Tafel plot in the manuscript of Figure 7. The values of the electrocatalytic performance of Mn(Mesbpy),[8] FeTPP,[9] Coqpy,[10] Ni(cyclam),[11] Fe-TDHP[7] and Fe-O-TMA[5] are obtained from references.

Supporting Tables

Table S2. Summary of the CPE experiments of complex **2** and **3** (0.5 mM) in 20 minutes

Complex	CPE-Potential	H_2, FE (%) (Trial 1)	H_2, FE (%) (Trial 2)	CO, FE (%) (Trial 1)	CO, FE (%) (Trial 2)
**2**	–2.37 V	7	11	18	15
**3**	-2.37 V	7.1	7.6	83.3	87.1
**3**	-2.55 V	14.7	-	48.1	-

Table S3: Comparison of calculated reduction potentials and Cu–ligand bond lengths of complex **3** using different DFT functionals.

Property	B3LYP	ωB97X-D	BPV86	Experimental conditions
**Reduction potential (V)**	- 1.52	- 1.57	- 1.60	- 1.53
**$Cu-N_{pyridine}$ bond length/ Å**	1.946	1.934	1.951	1.914
**$Cu-N_{amide}$ bond length/ Å**	2.022, 2.010	1.984, 1.990	2.021, 2.020	1.975, 1.977
**Cu-S bond length/ Å**	2.410, 2.875	2.467, 2.620	2.470, 2.532	2.379, 2.733

Table S4: EDA-NOCV results for intermediates **CO_2-2⁻** and **CO_2-3⁻**.

Energy terms/ kcal mol^{-1}	CO_2-3⁻	CO_2-2⁻
ΔE_{int}	-97.67	-90.67
$\Delta E_{orbital}$	**-46.99**	**-36.33**
ΔE_{elstat}	-21.85	-14.72
ΔE_{Pauli}	56.13	40.19
ΔE^0_{XC}	-23.53	-17.16
$\Delta E_{cpcm,dielectric}$	-59.81	-60.77
$\Delta E_{SMD-CDS,correction}$	-1.62	-1.87
$\Delta E_{orb(1)}$	**-46.90**	**-38.13**
$\Delta E_{orb(2)}$	**-2.34**	**-0.97**

$\Delta E_{orbital}$, ΔE_{elstat}, ΔE_{Pauli}, and ΔE_{xc}^0 represent the orbital, electrostatic, Pauli repulsion, and exchange–correlation contributions, respectively. $\Delta E_{CPCM,dielectric}$ and $\Delta E_{SMD-CDS}$ correction correspond to the continuum dielectric and SMD cavitation–dispersion–solvent-structure solvation corrections. $\Delta E_{orb(1)}$ and $\Delta E_{orb(2)}$ represent the two dominant NOCV orbital-interaction channels. Negative values indicate stabilizing interactions, whereas positive values indicate destabilizing contributions.

Characterization Data of Ligands and Complexes

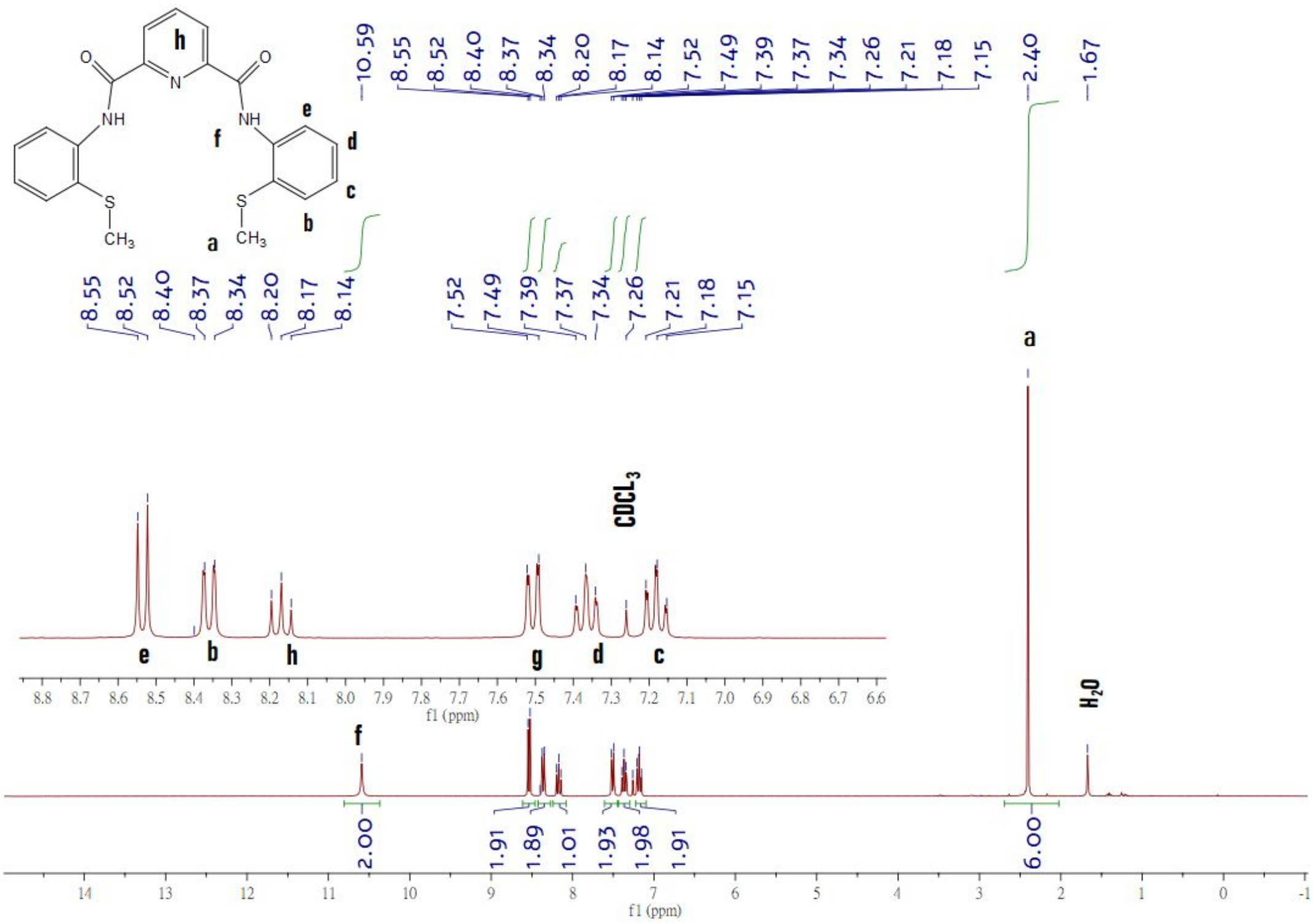

^{1}HNMR of non–cyclic N_3S_2 (**L_2**) in $CDCl_3$ (Top).

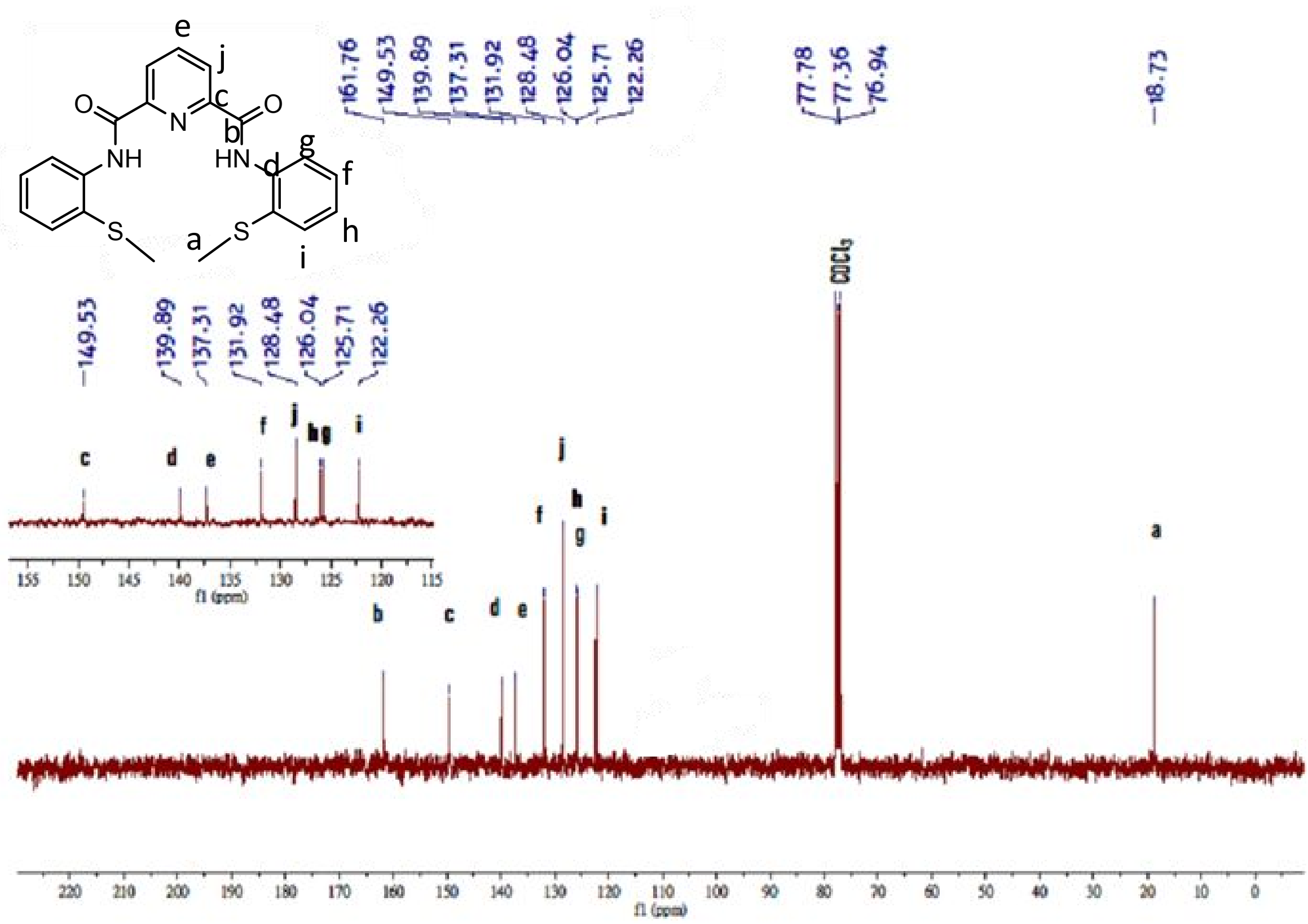

^{13}CNMR of non–cyclic N_3S_2 (**L_2**) in $CDCl_3$.

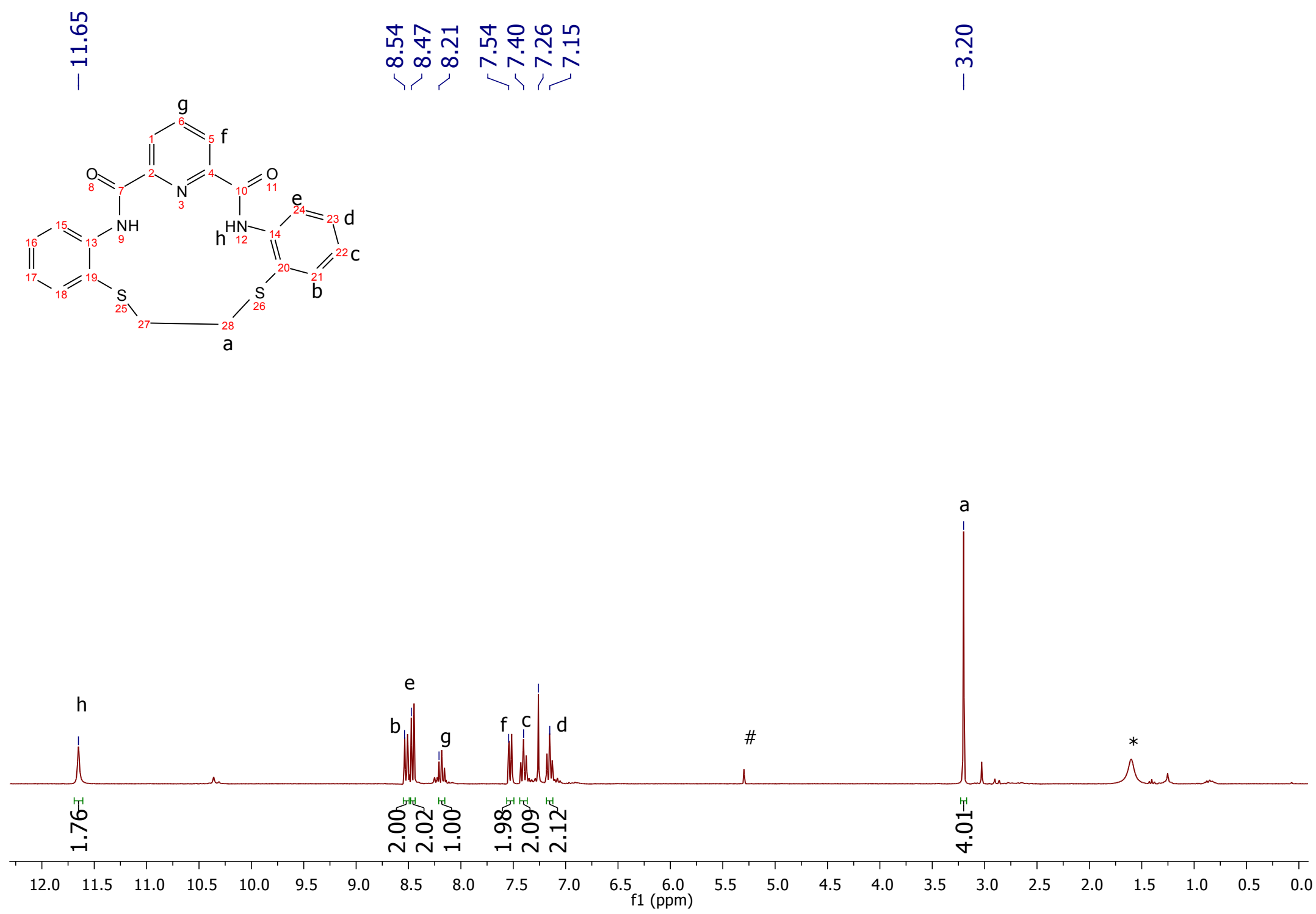

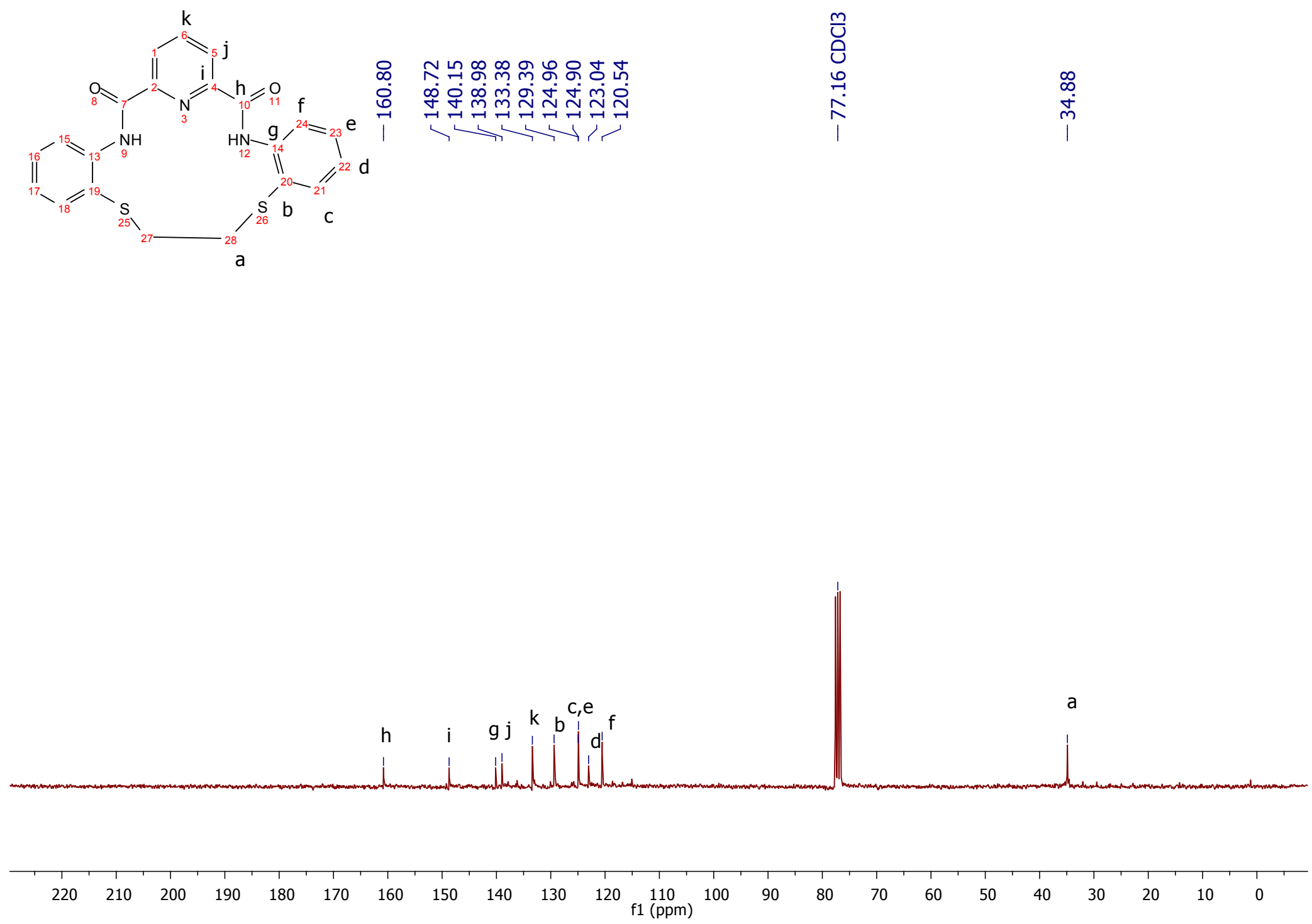

^{1}HNMR of non–cyclic N_3S_2 (**L3)** in $CDCl_3$ (Top). ^{13}CNMR of macrocyclic N_3S_2 (**L3)** in $CDCl_3$ (Bottom). (#,*: residual DCM and water).

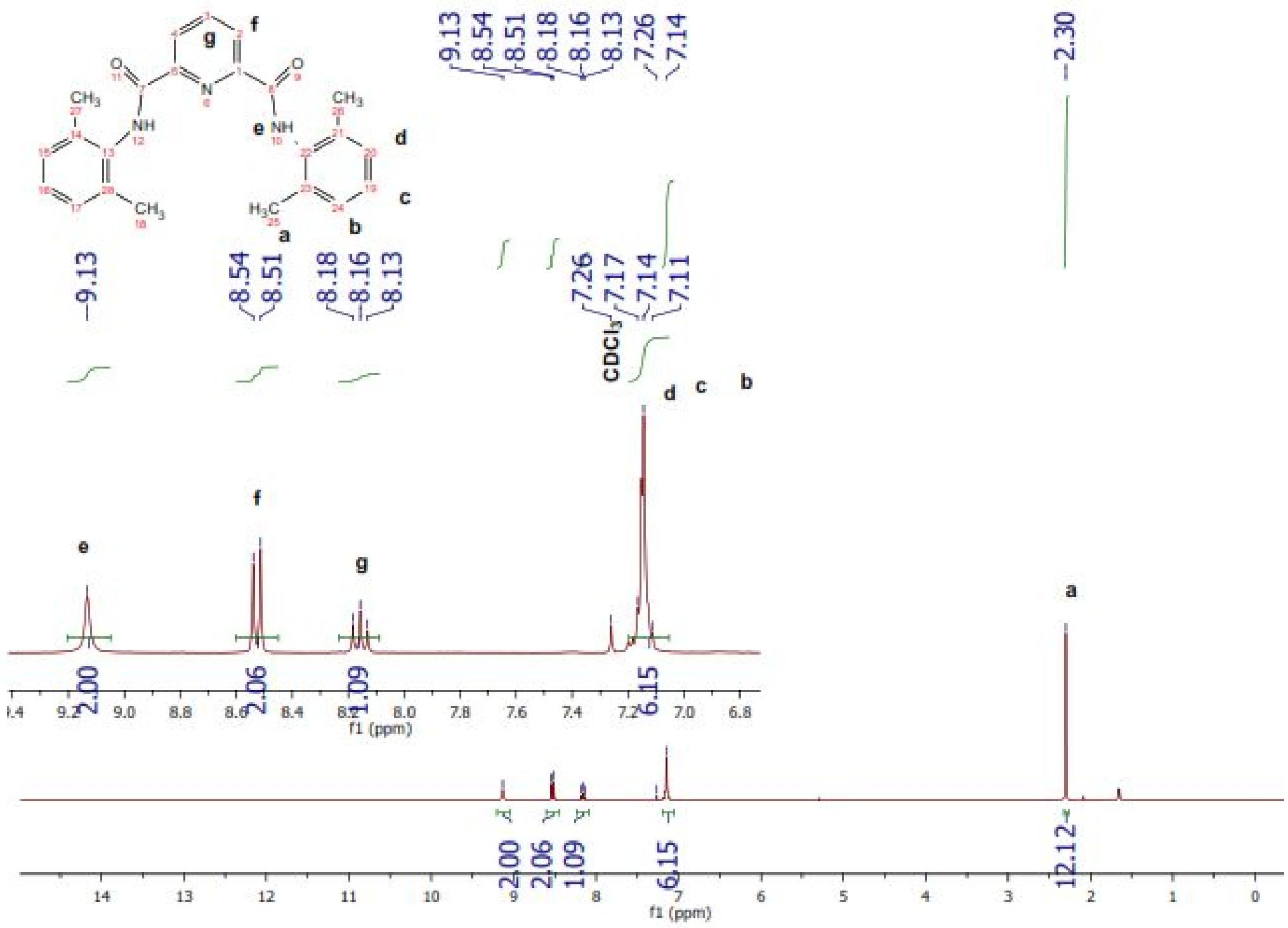

^{1}HNMR of $\mathbf{L_1^{Me}}$ in $CDCl_3$.

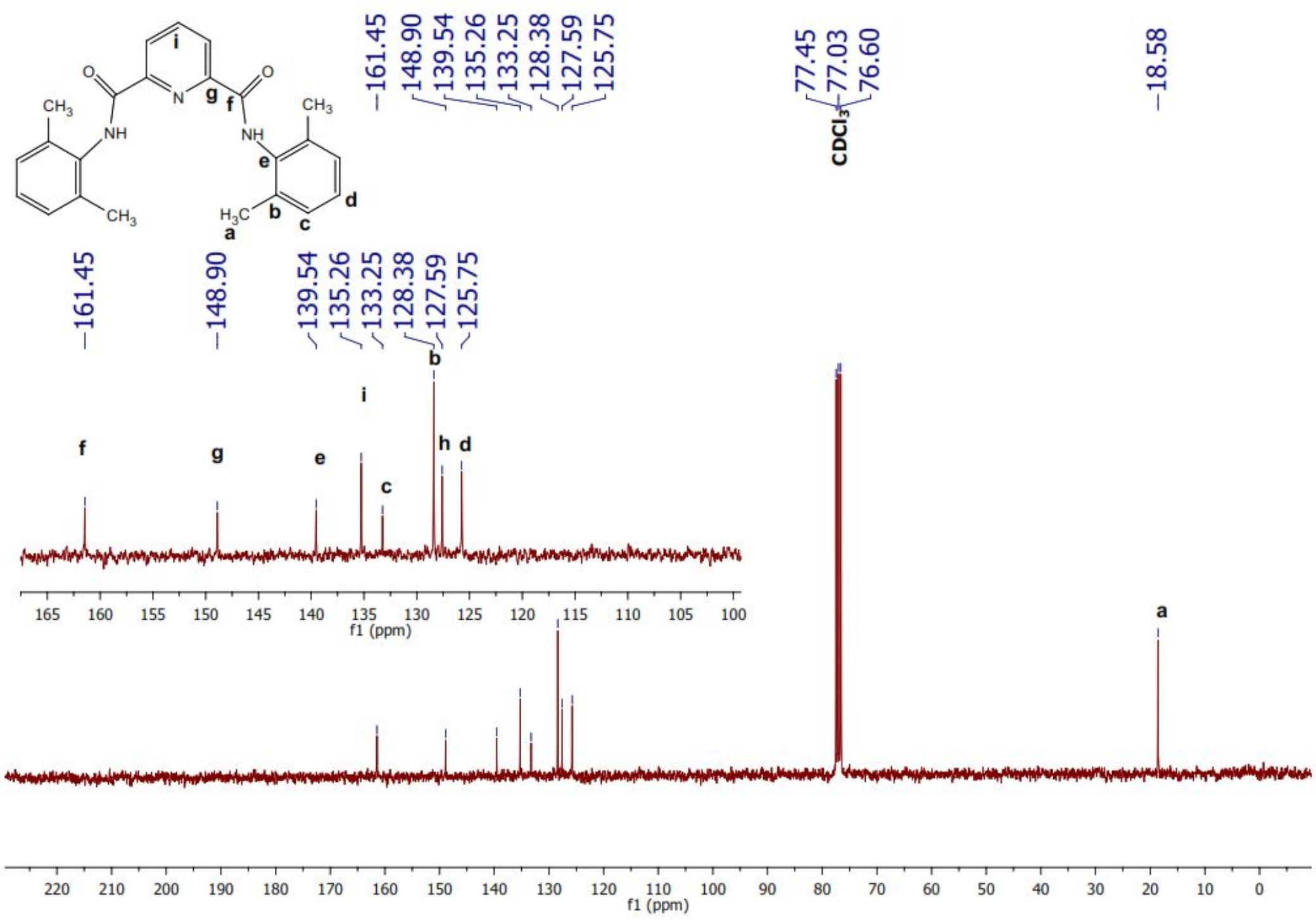

^{13}CNMR of $\mathbf{L_1^{Me}}$ in $CDCl_3$.

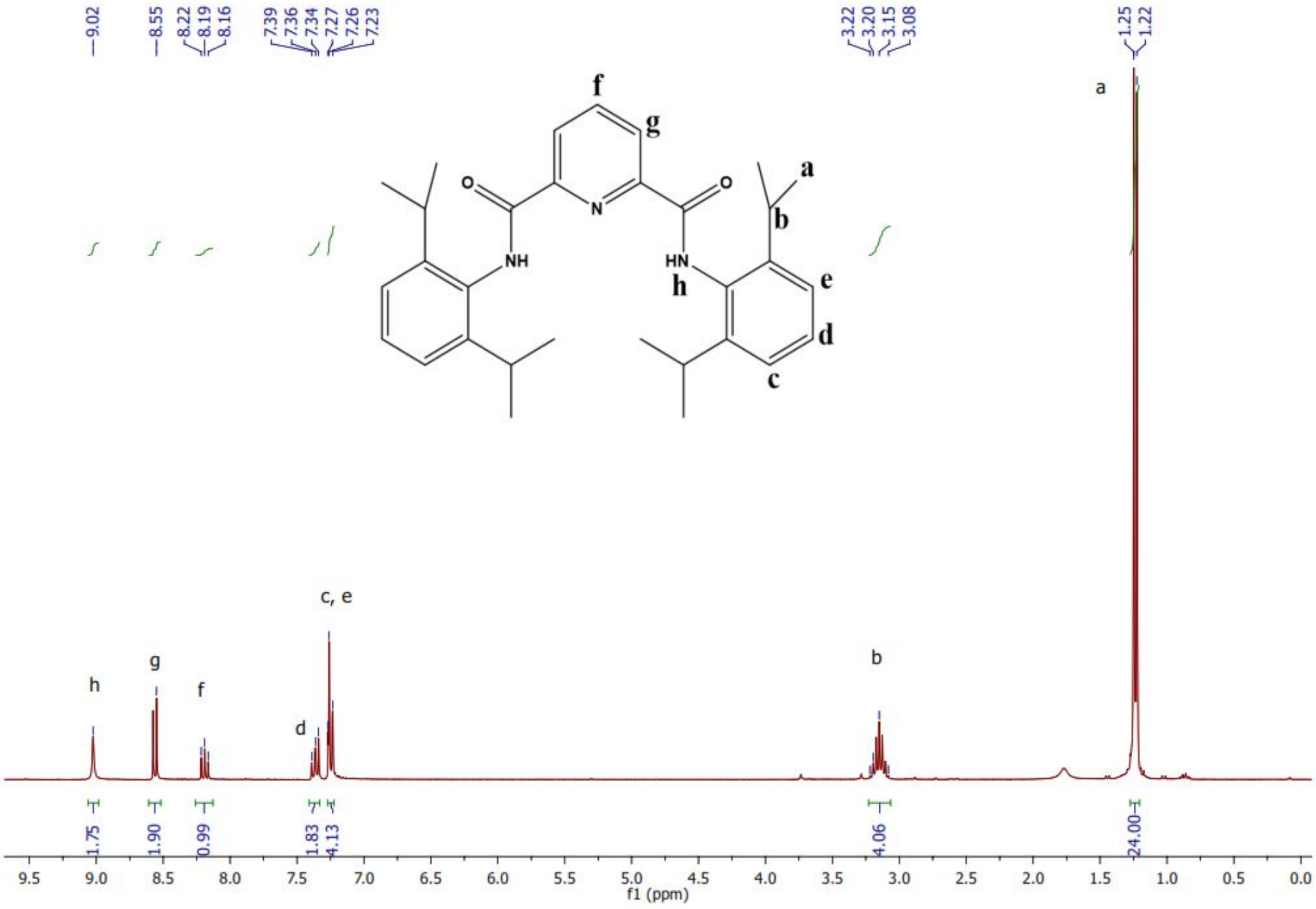

^{1}HNMR of $\mathbf{L_1}^{\boldsymbol{iPr}}$ in $CDCl_3$.

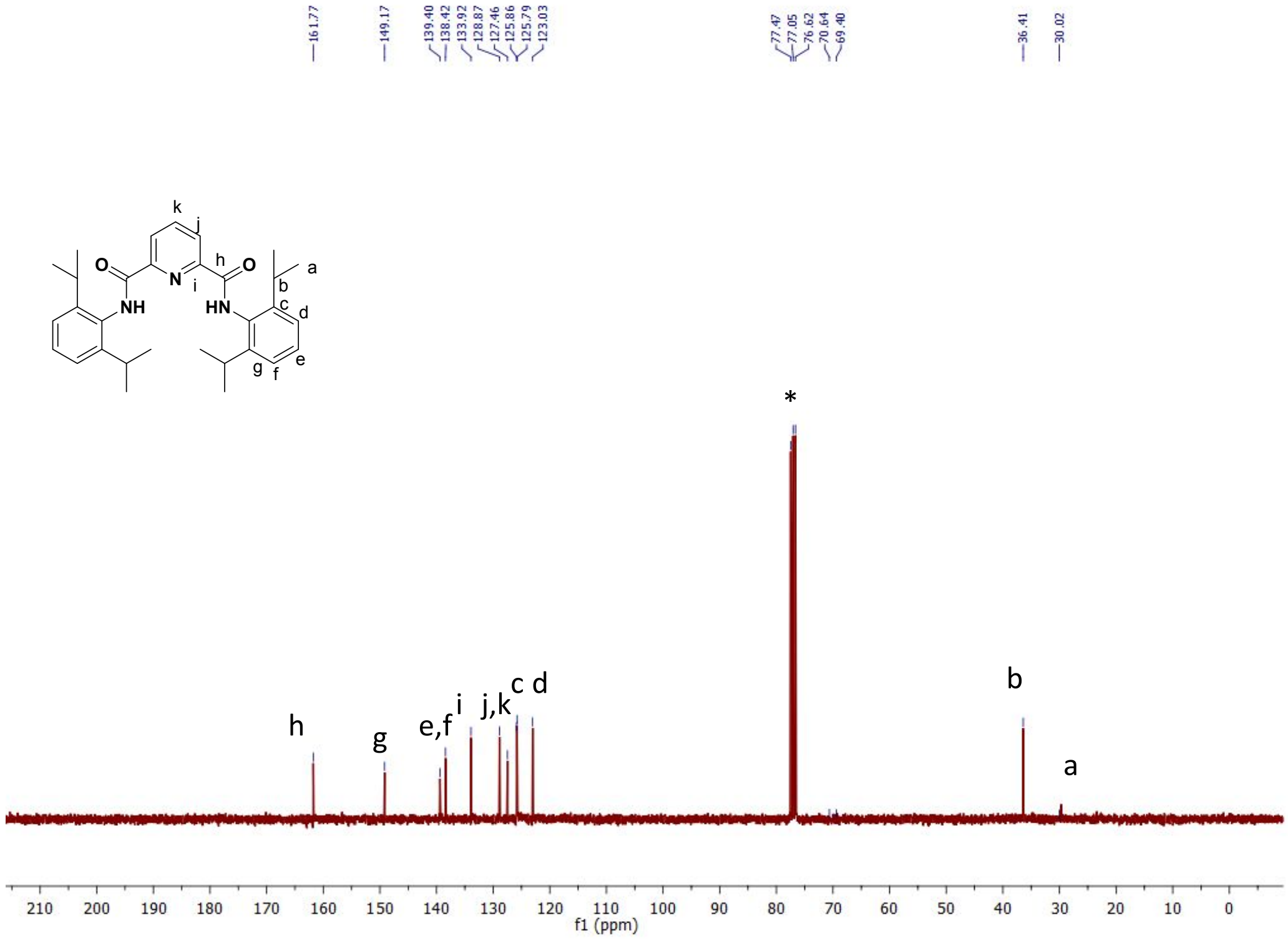

^{13}CNMR of $\mathbf{L_1}^{\boldsymbol{iPr}}$ in $CDCl_3$.

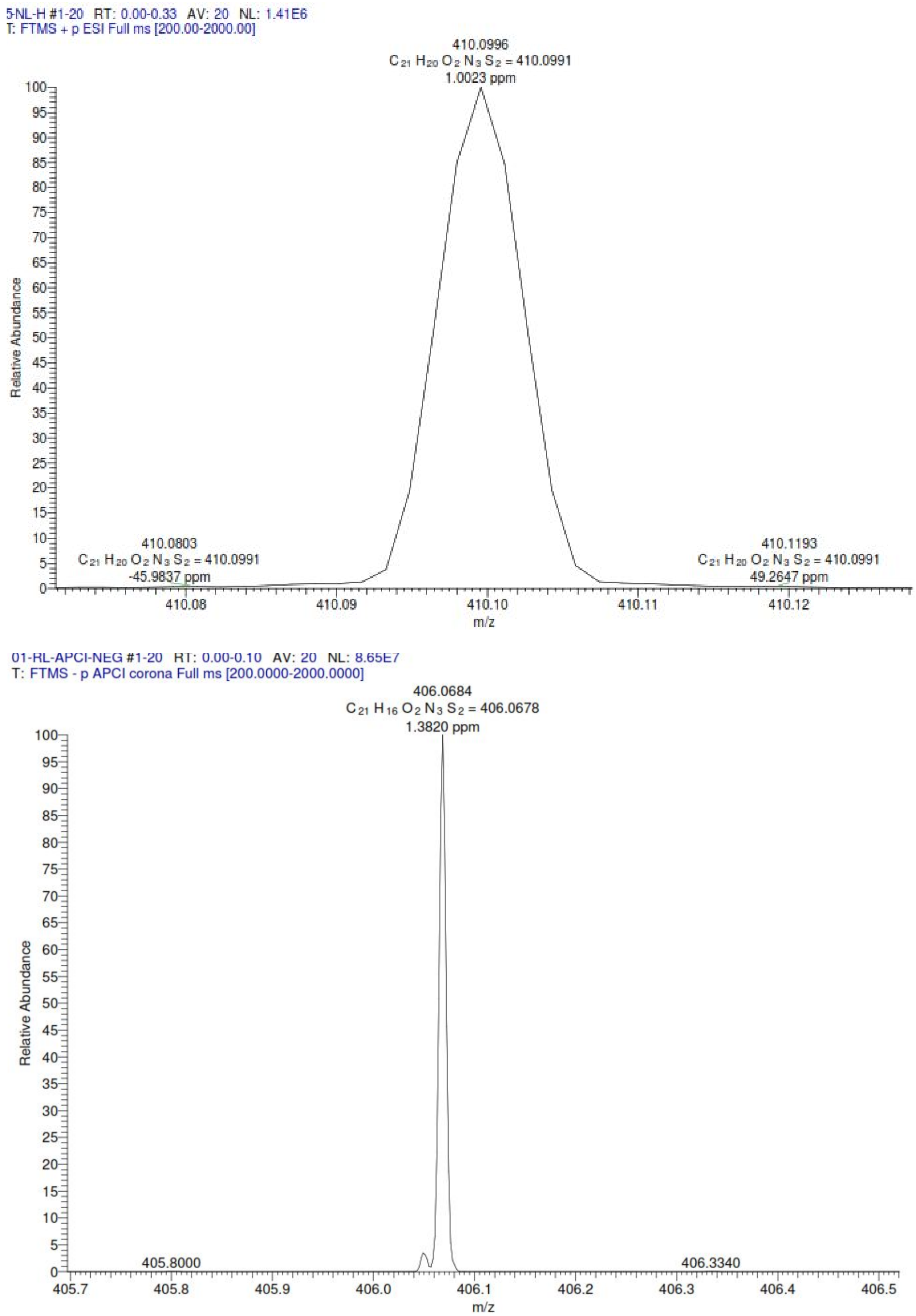

HRMS of non–cyclic N_3S_2 (**L_2+H**)$^+$ in chloroform (Top) and HRMS of macrocyclic N_3S_2 (**L_3-H)**$^-$ in chloroform (Bottom).

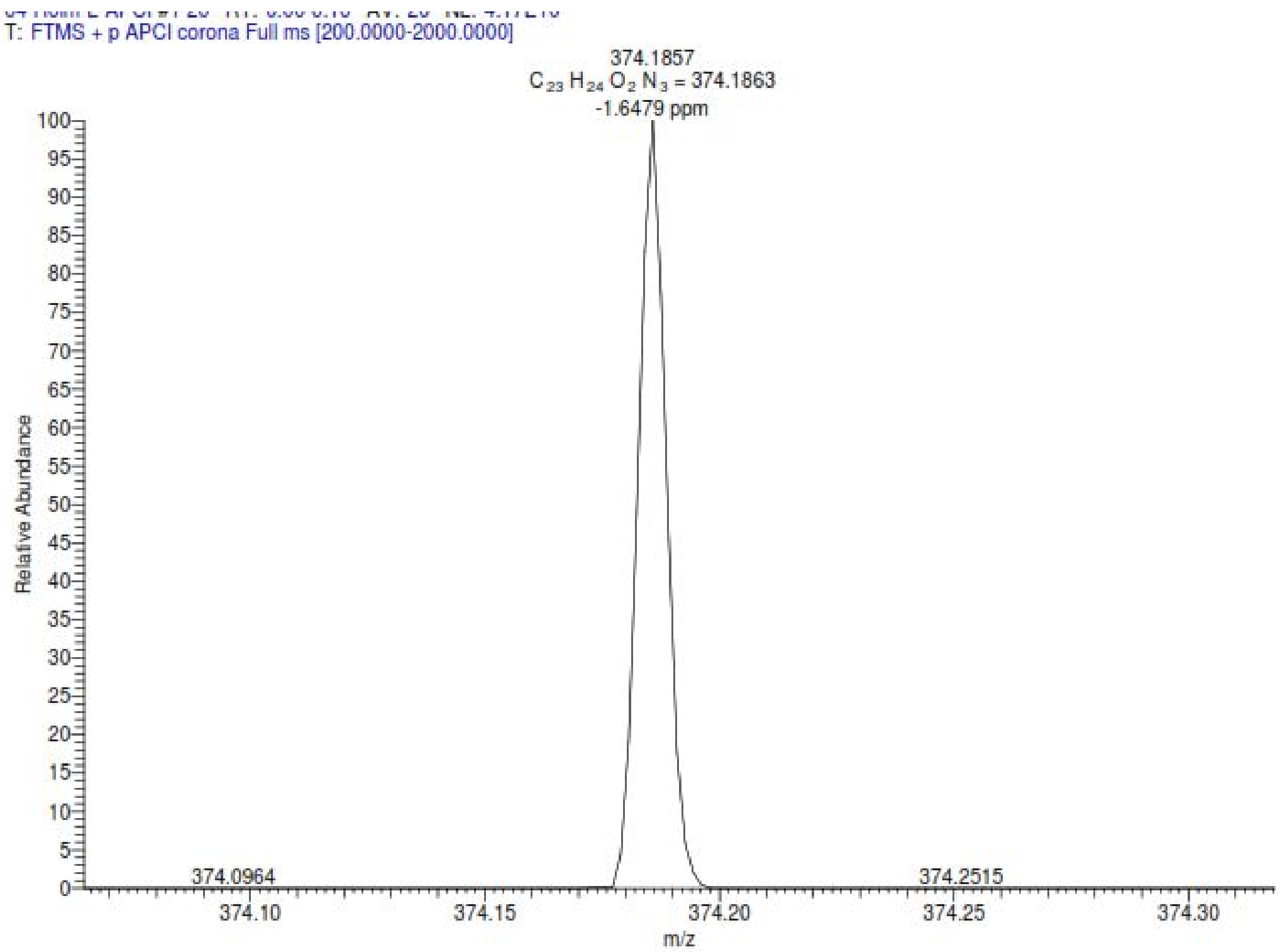

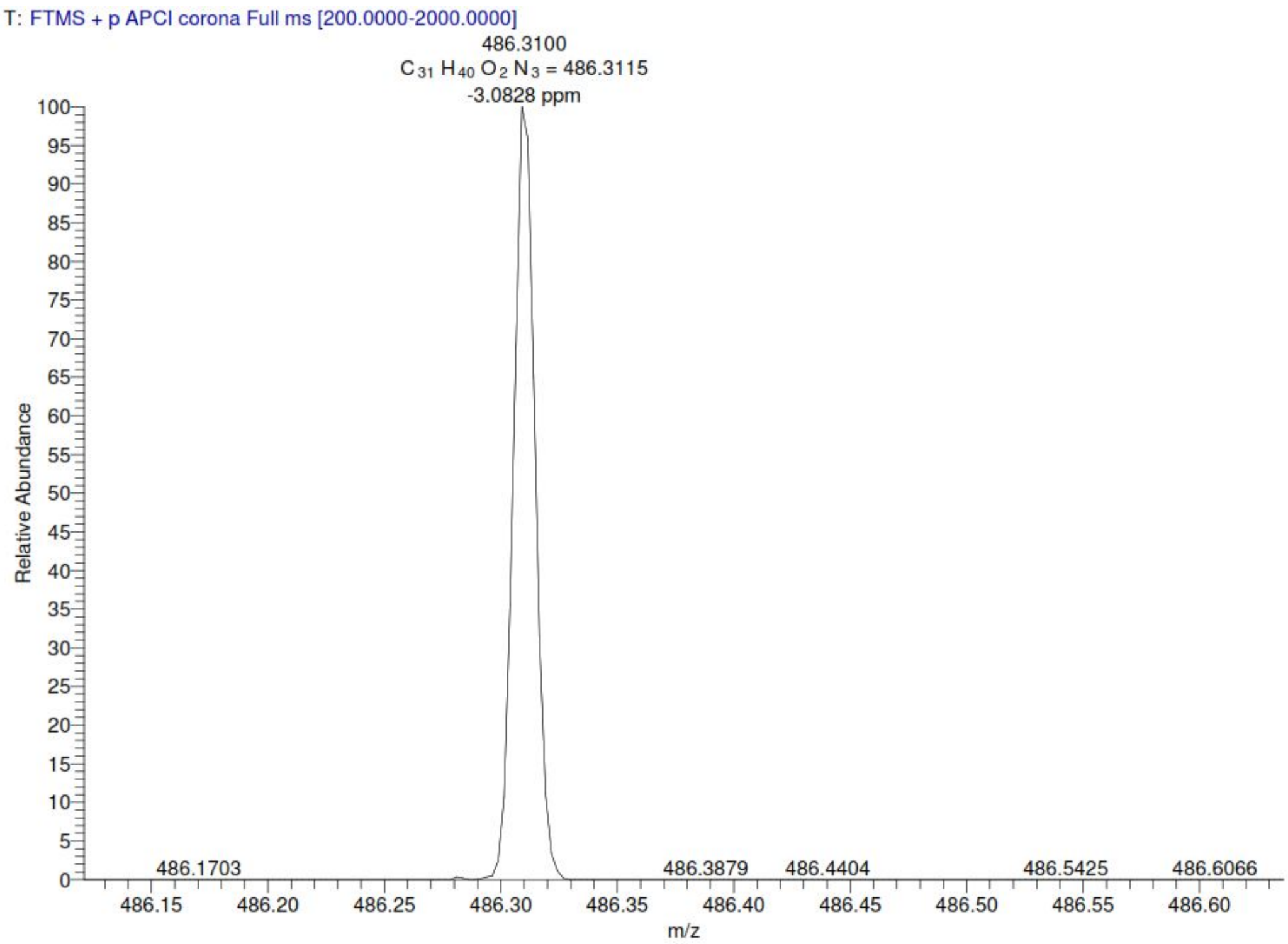

HRMS of ($L_1^{Me} + H^+$) in chloroform (Top) and ($L1^{iPr} + H^+$) in chloroform (Bottom)

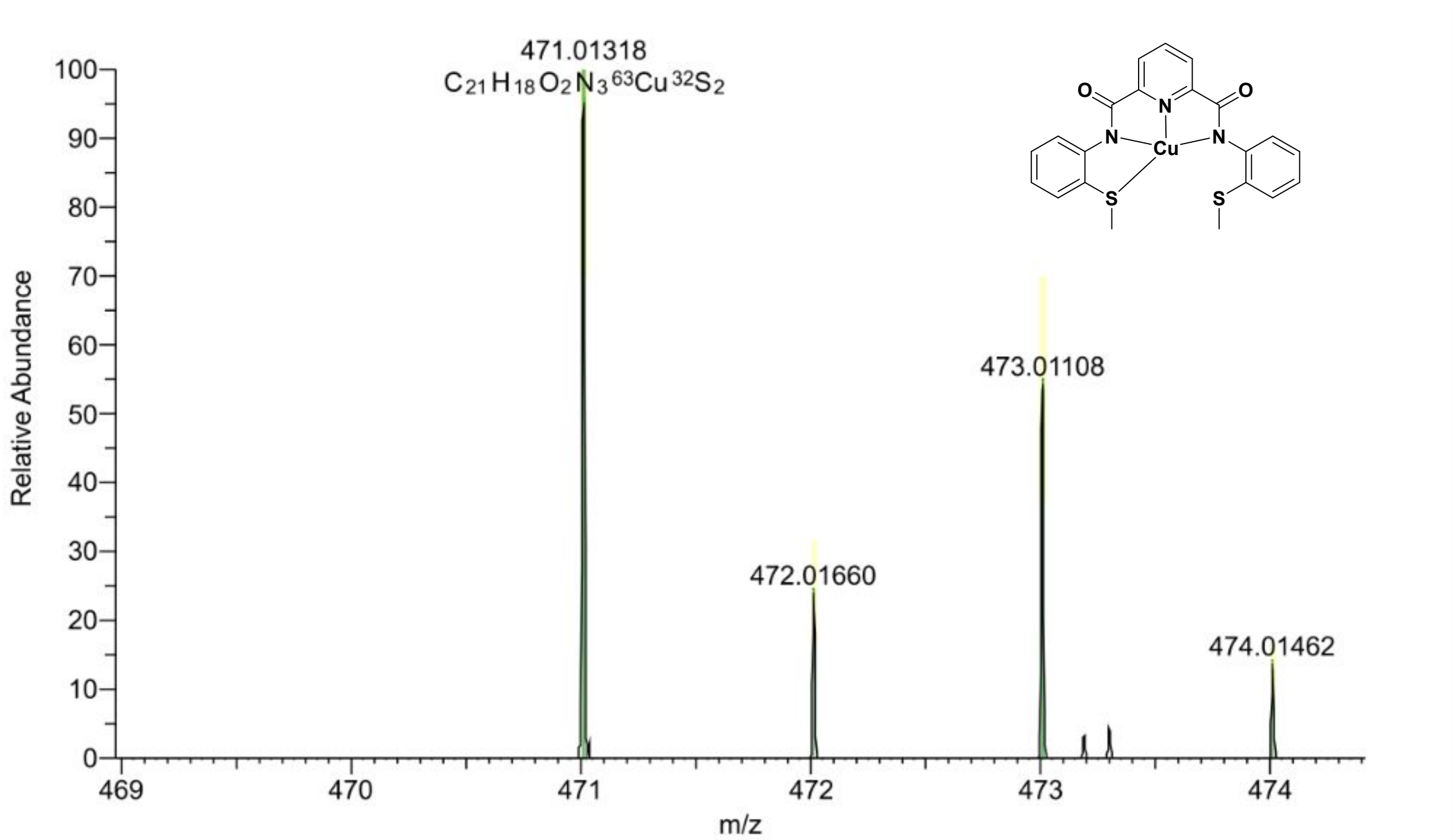

Peak Mass	Display Formula	S Fit	RDB	Delta [ppm]	Theo. mass	Pattern Cov. [%]	MSMS Matched...
471.01318	$C_{21}H_{18}O_2N_3{}^{63}Cu^{32}S_2$	41.2769868672978	14.50	0.19	471.01310	97.23	(Collection)

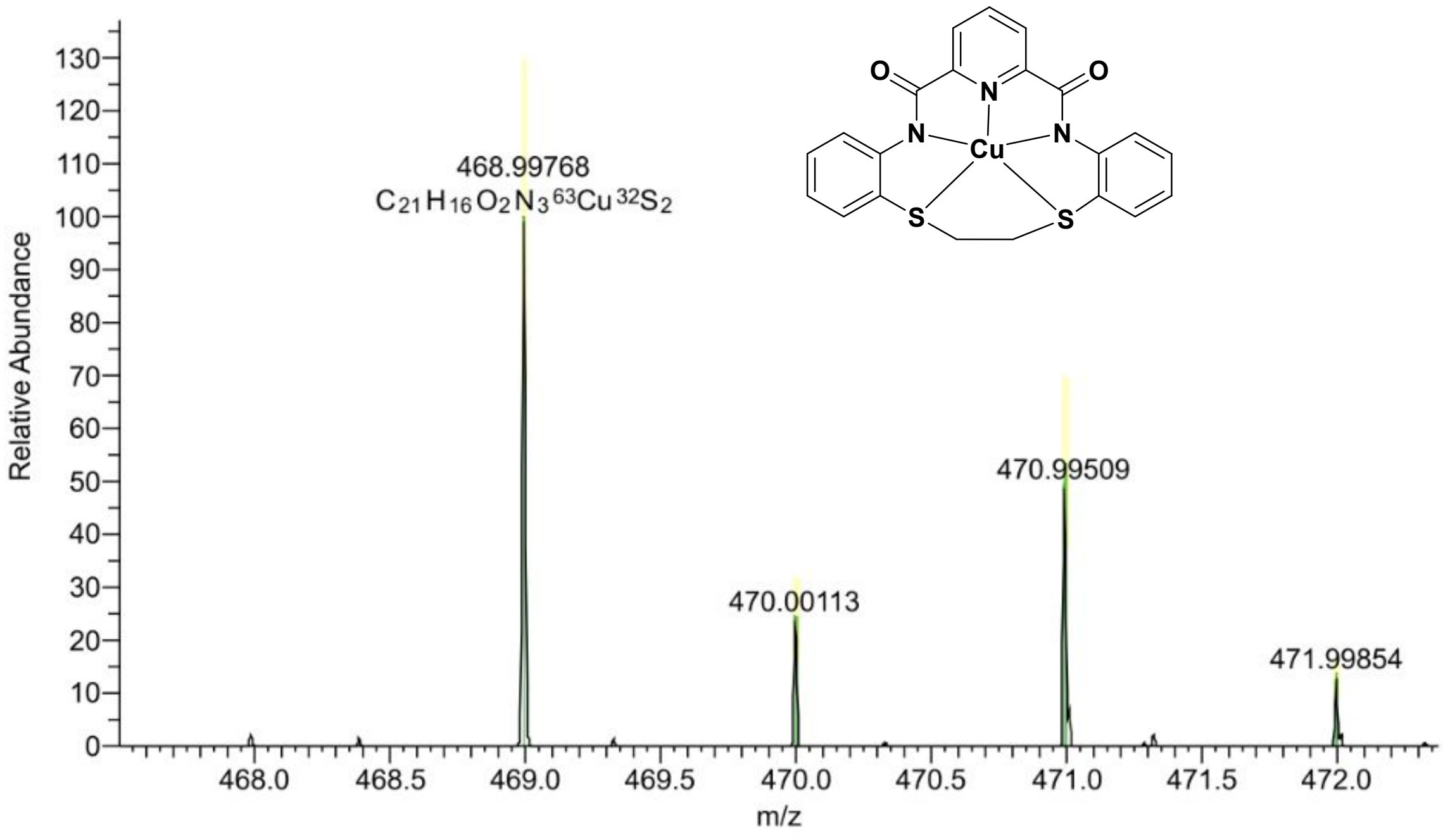

Peak Mass	Display Formula	S Fit	RDB	Delta [ppm]	Theo. mass	Pattern Cov. [%]	MSMS Matched..
468.99768	$C_{21}H_{16}O_2N_3{}^{63}Cu^{32}S_2$	48.6225752016116	15.50	0.50	468.99745	97.67	(Collection)

HRMS of complex (Top) **2** (M+1); (bottom) **3** (M+1) in acetonitrile

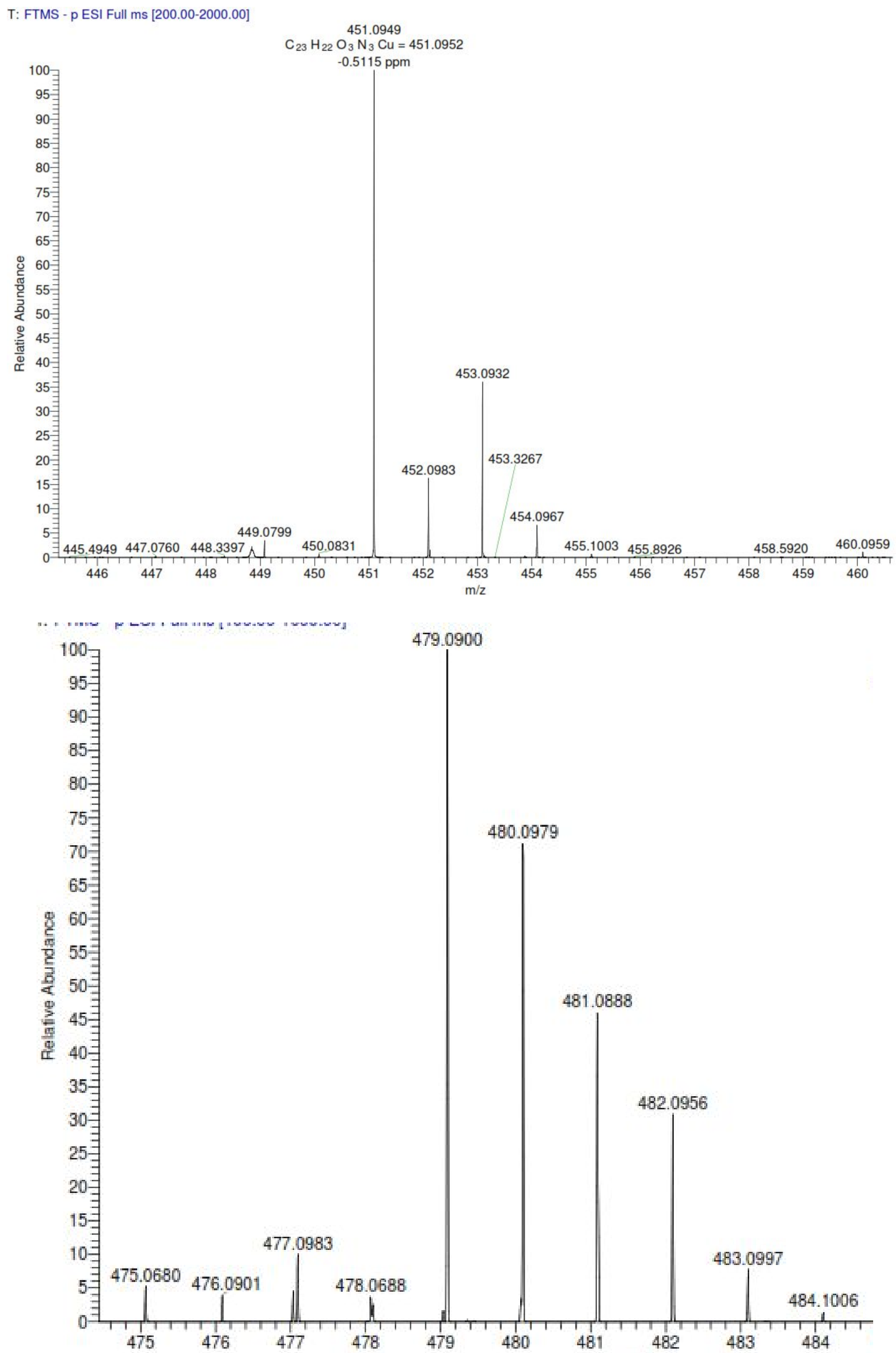

HRMS of complex (Top) $\mathbf{1^{Me}}$**-OH**: $Et_4N[1^{Me}\text{-}OH]$ (M-1); (bottom) $\mathbf{1^{Me}}$: $Et_4N[1^{Me}\text{-}HCO_2]$ (M-1) in acetonitrile

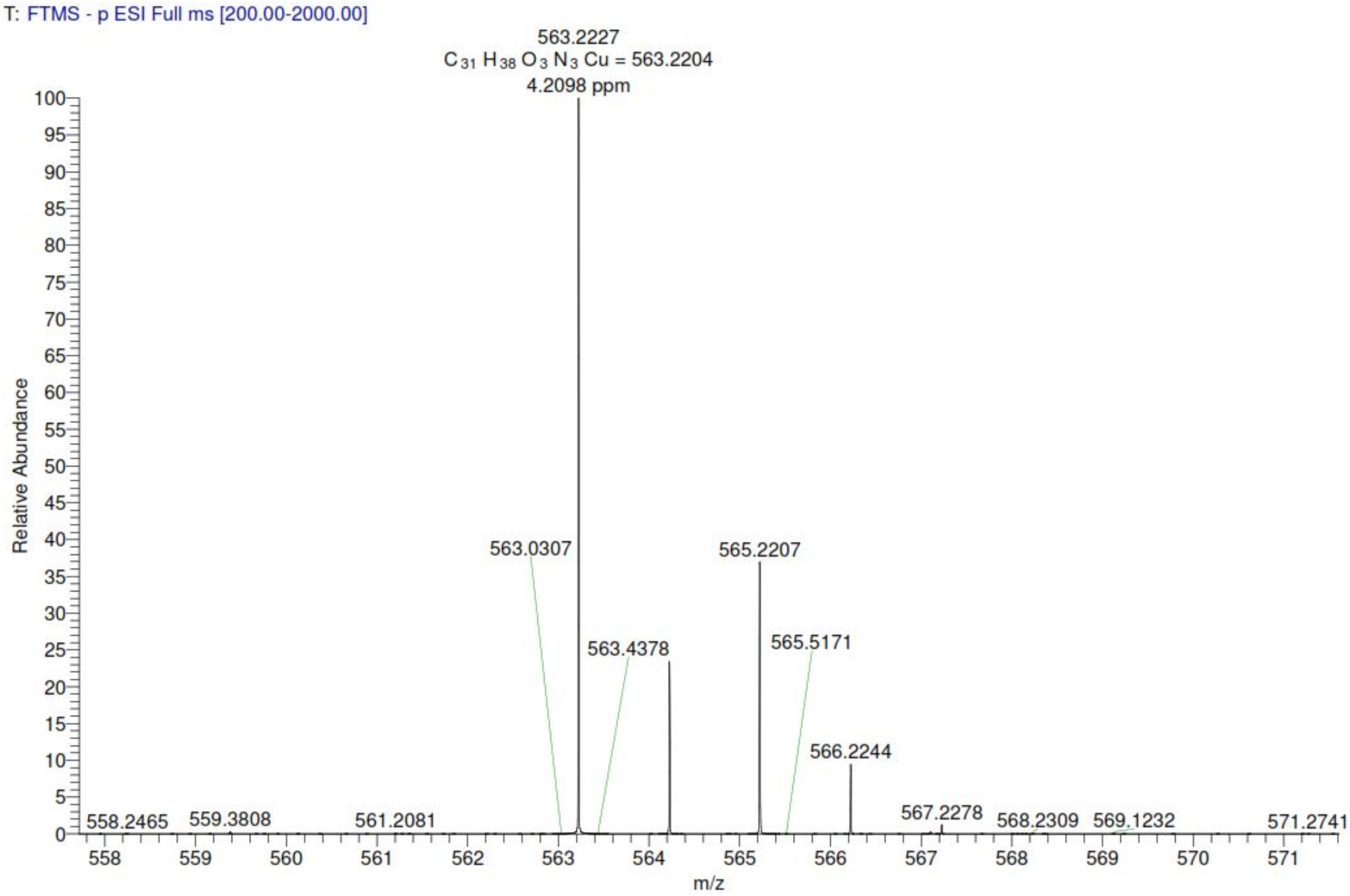

HRMS of complex **1*iPr***: Et_4N [1^{iPr}-OH] (M+1) in acetonitrile

-

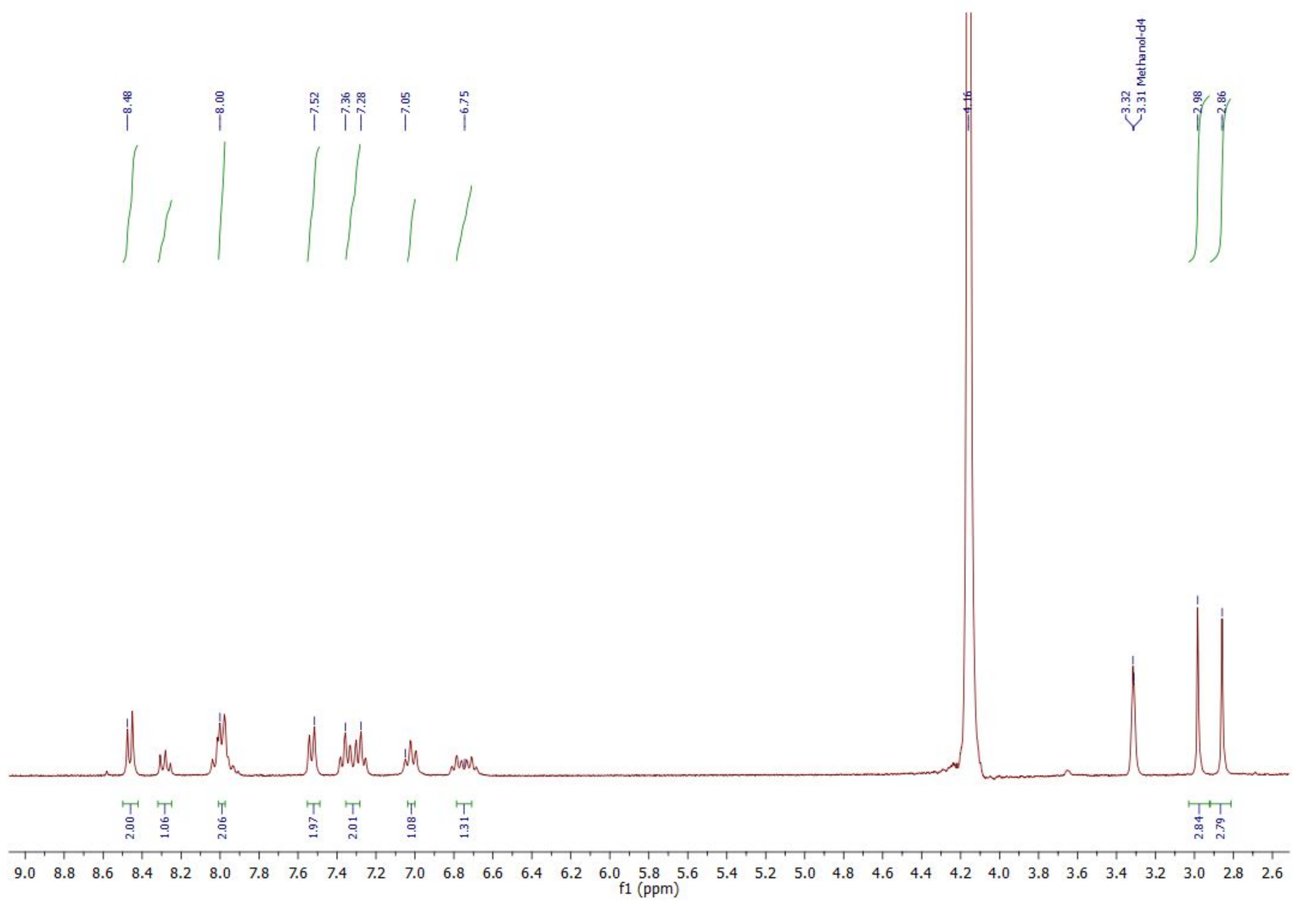

^{1}H-NMR of **2^{Zn}** in CD_3OD

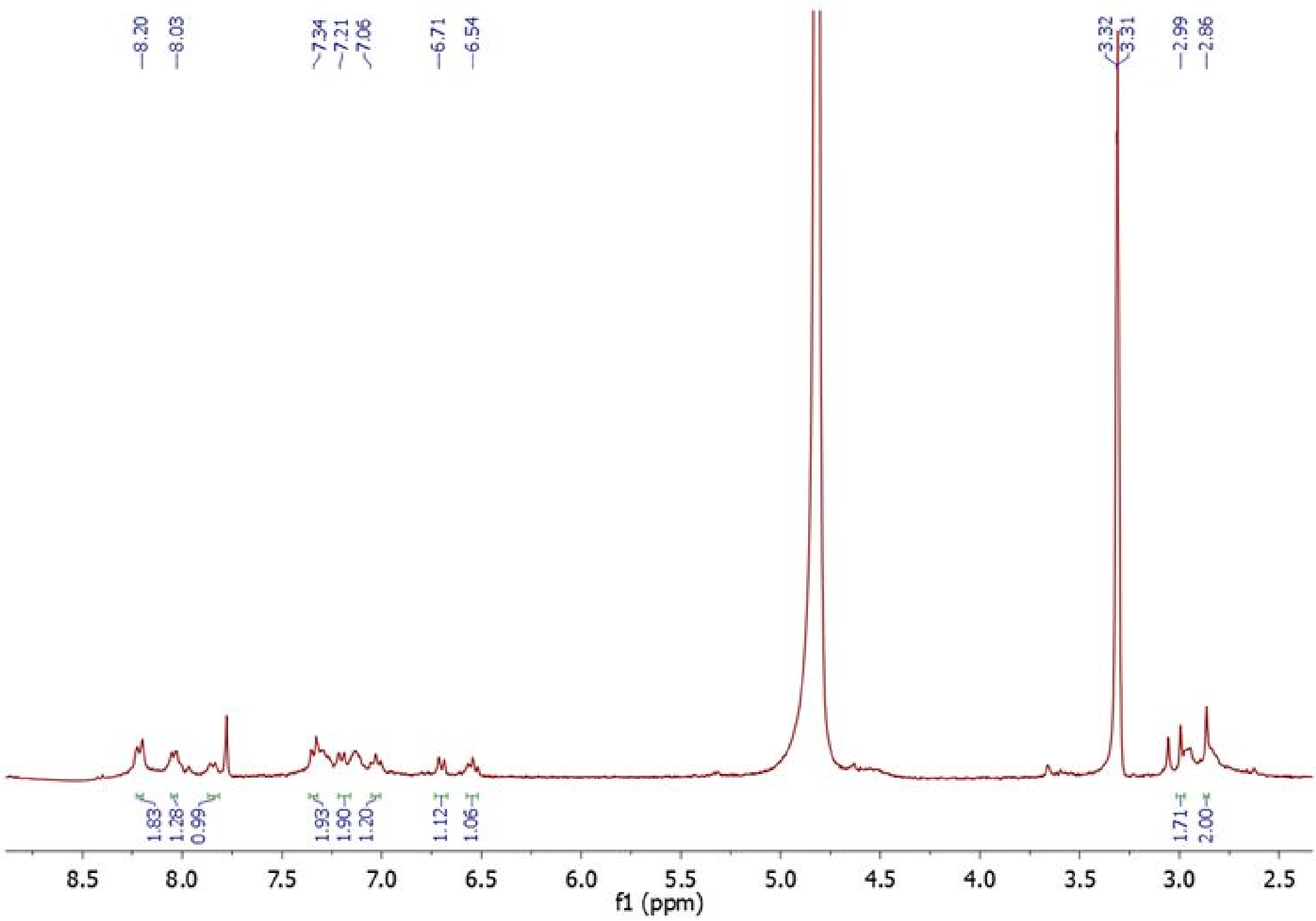

^{1}H-NMR of **3**^{Zn} in CD_3OD

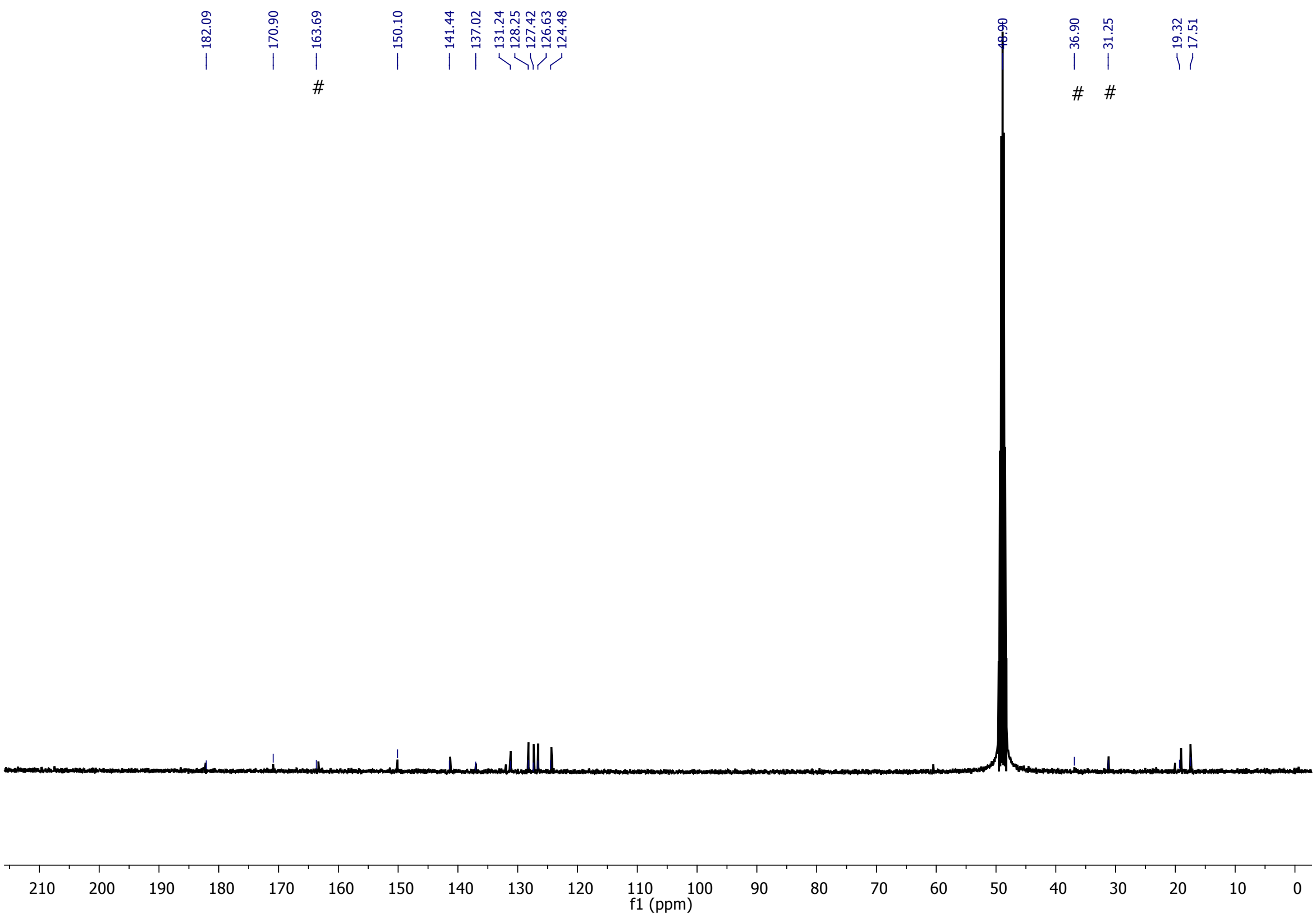

^{13}C-NMR of **2**$^{\mathbf{Zn}}$ in CD_3OD (#: residual DMF solvent).

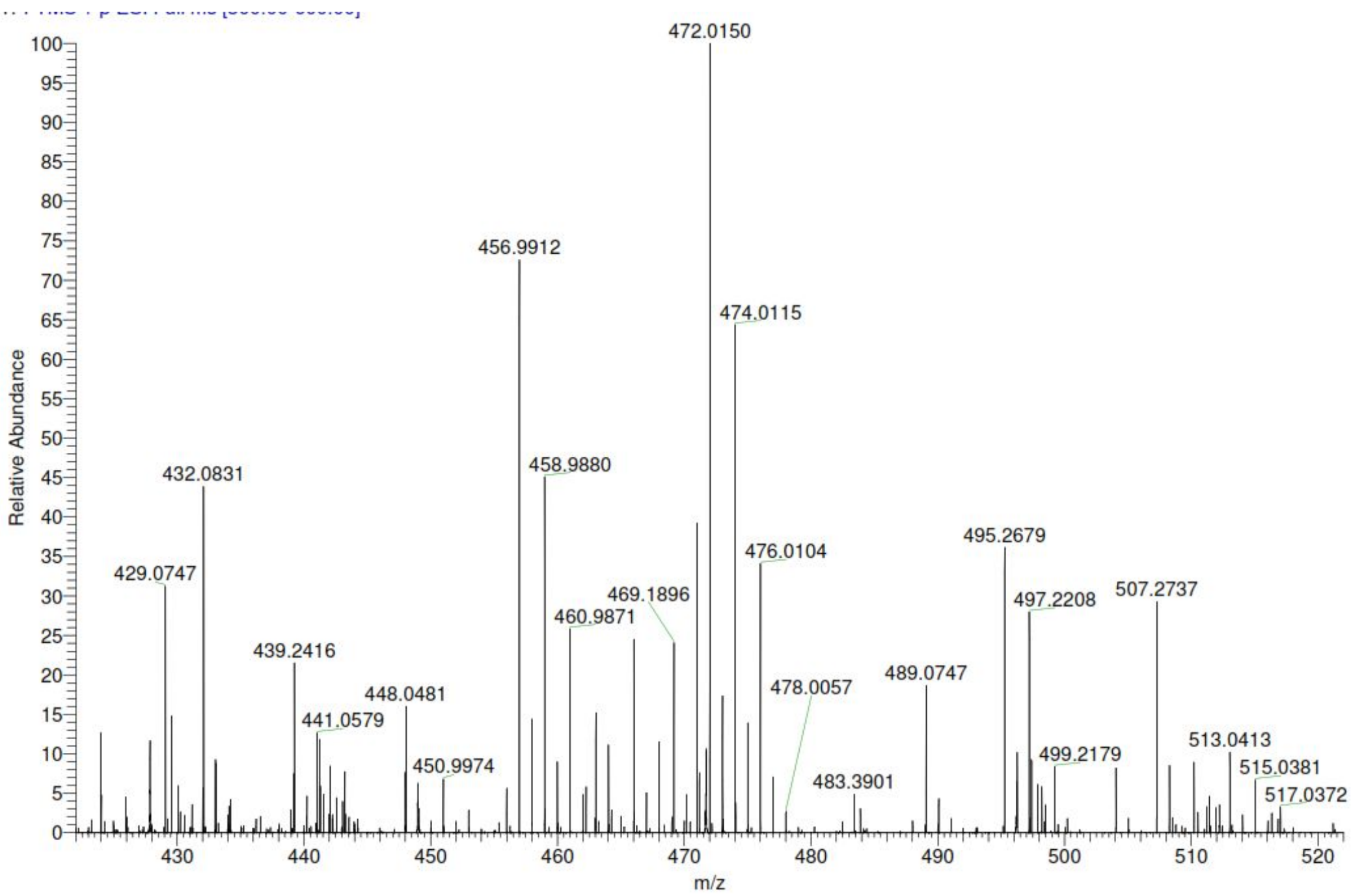

ESI-MS of 2^{Zn} in an EtOH mixture showing the $(M+H)^+$ peak.

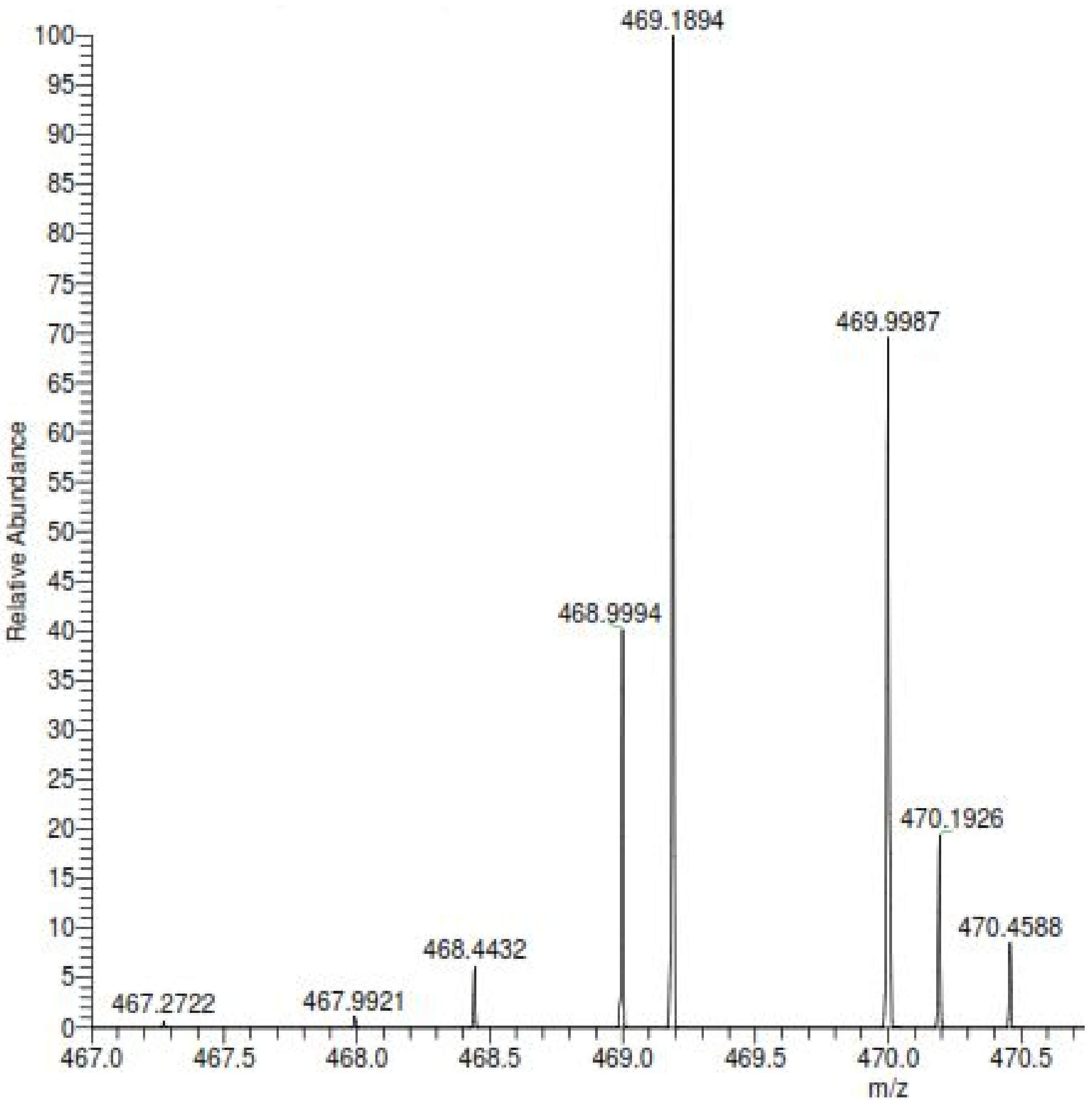

ESI-MS of 3^{Zn} in an EtOH mixture showing the $(M+H)^+$ peak.

Coordinates of optimized structure

1^{Me} Charge = [-1], Multiplicity = [2]

C	-1.22999	4.14649	-0.04280
C	-1.18543	2.75910	-0.05805
N	-0.01320	2.13627	-0.06065
C	1.15379	2.76888	-0.05479
C	1.18667	4.15661	-0.03929
C	-0.02457	4.84344	-0.03244
C	-2.38827	1.83927	-0.07612
O	-3.52611	2.33038	-0.07031
N	-2.04078	0.54521	-0.10577
C	2.36442	1.85920	-0.07083
O	3.49805	2.35998	-0.06323
N	2.02797	0.56223	-0.10178
C	-3.05463	-0.44058	-0.08302
C	3.05024	-0.41500	-0.08270
C	-3.70155	-0.76540	1.12550
C	-4.65812	-1.78121	1.12784
C	-4.97765	-2.46960	-0.03595
C	-4.33663	-2.14054	-1.22391
C	-3.37539	-1.13026	-1.26768
C	3.70034	-0.73886	1.12431
C	4.66543	-1.74657	1.12244
C	4.99027	-2.42779	-0.04408
C	4.34628	-2.09945	-1.23057
C	3.37658	-1.09719	-1.27017
C	-2.69771	-0.78478	-2.56512
C	3.36605	-0.01645	2.40048
C	-3.37300	-0.03552	2.39891
C	2.69640	-0.75167	-2.56621
Cu	-0.00486	0.18537	-0.11087
O	0.00567	-1.73826	-0.41799
H	-2.18271	4.65629	-0.04048
H	2.13507	4.67441	-0.03425
H	-0.02913	5.92615	-0.02040
H	-5.15449	-2.03363	2.05837
H	-5.72083	-3.25798	-0.01688
H	-4.58360	-2.67159	-2.13648
H	5.16412	-1.99847	2.05183
H	5.73992	-3.21007	-0.02827
H	4.59742	-2.62485	-2.14526
H	-3.08344	-1.40270	-3.37717
H	-1.61859	-0.94144	-2.50019
H	-2.85244	0.26325	-2.83485
H	2.29394	-0.04329	2.60940
H	3.65240	1.03691	2.34665
H	3.88884	-0.46574	3.24574
H	-2.30077	-0.05259	2.60744
H	-3.89163	-0.48608	3.24602
H	-3.66823	1.01506	2.34139
H	3.08551	-1.36463	-3.38028
H	2.84499	0.29804	-2.83261
H	1.61826	-0.91477	-2.50142
C	0.04355	-2.71022	0.40168
O	0.10127	-3.90660	0.12889
O	0.01090	-2.32908	1.72810
H	0.04769	-3.14656	2.24594

1^{Me-} Charge = [-2], Multiplicity = [1]

C	1.20177	4.06314	-0.14538
C	1.16581	2.67356	-0.02297
N	0.00002	2.01419	0.05518
C	-1.16576	2.67356	-0.02303
C	-1.20166	4.06316	-0.14543
C	0.00005	4.76393	-0.20224
C	2.44860	1.85256	-0.00015
O	3.53893	2.48754	0.06462
N	2.25260	0.54947	-0.08047
C	-2.44862	1.85265	-0.00026
O	-3.53890	2.48781	0.06432
N	-2.25276	0.54958	-0.08041
C	3.35703	-0.31939	-0.06638
C	-3.35721	-0.31921	-0.06640
C	3.82207	-0.87471	-1.28126
C	4.86460	-1.80617	-1.25552
C	5.45183	-2.19863	-0.05378
C	4.99000	-1.64886	1.14141
C	3.95110	-0.71332	1.15596
C	-3.82205	-0.87472	-1.28128
C	-4.86457	-1.80618	-1.25558
C	-5.45203	-2.19847	-0.05389
C	-4.99042	-1.64850	1.14129
C	-3.95153	-0.71296	1.15589
C	3.46613	-0.13060	2.46040
C	-3.20077	-0.45737	-2.59166
C	3.20095	-0.45725	-2.59169
C	-3.46679	-0.13005	2.46034
Cu	0.00078	-0.02624	0.19915
O	-0.00003	-1.91762	0.91053
H	2.15949	4.56206	-0.19818
H	-2.15938	4.56208	-0.19826
H	0.00006	5.84400	-0.29918
H	5.21888	-2.22710	-2.19201
H	6.25815	-2.92420	-0.04824
H	5.44167	-1.94888	2.08258
H	-5.21867	-2.22727	-2.19208
H	-6.25833	-2.92404	-0.04841
H	-5.44228	-1.94834	2.08243
H	4.00102	-0.56971	3.30553
H	2.39544	-0.30700	2.60100
H	3.61221	0.95347	2.48910
H	-2.12395	-0.65171	-2.60113
H	-3.32377	0.61548	-2.77057
H	-3.65599	-0.99473	-3.42675
H	2.12431	-0.65256	-2.60170
H	3.65697	-0.99381	-3.42685
H	3.32306	0.61580	-2.76999
H	-4.00263	-0.56824	3.30536
H	-3.61183	0.95416	2.48842
H	-2.39636	-0.30745	2.60174
C	-0.00036	-3.01480	0.29136
O	-0.00067	-4.16379	0.75996
O	-0.00036	-2.88201	-1.09654
H	-0.00060	-3.77735	-1.45976

1^{Me2-} Charge = [-3], Multiplicity = [2]

C	-1.17642	3.92342	0.23391
C	-1.18892	2.54498	0.09834
N	0.00289	1.80490	-0.04096
C	1.18539	2.52163	-0.04560
C	1.21510	3.89389	0.06732
C	0.01761	4.64324	0.22165
C	-2.49614	1.83013	0.08593
O	-3.56685	2.48148	0.24926
N	-2.41766	0.50774	-0.13331
C	2.52605	1.81237	-0.16007
O	3.51533	2.48827	-0.56043
N	2.54606	0.54558	0.23157
C	-3.55821	-0.28650	-0.09655
C	3.68135	-0.23397	0.10176
C	-4.20805	-0.59583	1.12590
C	-5.29372	-1.47051	1.12983
C	-5.76106	-2.05352	-0.04432
C	-5.12353	-1.75512	-1.24410
C	-4.03511	-0.88439	-1.29018
C	4.29411	-0.75785	1.27029
C	5.37523	-1.63041	1.16120
C	5.87572	-2.01071	-0.08005
C	5.27506	-1.50521	-1.22904
C	4.19266	-0.62854	-1.16426
C	-3.37779	-0.57076	-2.60769
C	3.77513	-0.36344	2.62653
C	-3.71214	-0.00253	2.41554
C	3.55667	-0.12854	-2.43136
Cu	-0.31860	-0.13699	-0.27514
O	-0.10518	-2.07377	-0.65974
H	-2.13467	4.41564	0.34421
H	2.18276	4.37563	0.03759
H	0.03417	5.72058	0.33019
H	-5.77853	-1.69996	2.07346
H	-6.60532	-2.73262	-0.02365
H	-5.47895	-2.20071	-2.16775
H	5.83289	-2.01511	2.06705
H	6.71533	-2.69212	-0.15120
H	5.64972	-1.80041	-2.20410
H	-3.86485	-1.11075	-3.42142
H	-2.31833	-0.83959	-2.60413
H	-3.42202	0.49759	-2.83726
H	2.71892	-0.62120	2.74048
H	3.84611	0.71570	2.79120
H	4.33743	-0.86265	3.41821
H	-2.65710	-0.24017	2.58256
H	-4.28388	-0.38185	3.26455
H	-3.79011	1.08719	2.40159
H	4.01246	-0.59606	-3.30714
H	3.65745	0.95681	-2.51845
H	2.48349	-0.34444	-2.44934
C	0.04100	-3.02708	0.15402
O	0.16368	-4.23104	-0.09357
O	0.05984	-2.63983	1.49178
H	0.16876	-3.45595	1.99878

2 Charge = [0], Multiplicity = [2]

C	1.92953	3.98809	0.15761
C	1.74690	2.61383	0.07867
N	0.52361	2.13178	-0.08436
C	-0.56959	2.87037	-0.18651
C	-0.46506	4.25288	-0.11618
C	0.80339	4.80358	0.05910
C	2.83257	1.55795	0.16118
O	4.00275	1.90878	0.31806
N	2.32625	0.30282	0.04360
C	-1.83861	2.06237	-0.38134
O	-2.91991	2.64018	-0.51355
N	-1.60263	0.73224	-0.37816
C	3.07340	-0.86673	0.10101
C	-2.68153	-0.15527	-0.58520
C	4.46731	-0.93235	0.30259
C	5.12807	-2.15109	0.35660
C	4.43941	-3.35201	0.22176
C	3.06532	-3.31716	0.02861
C	2.39279	-2.10167	-0.04412
C	-3.23662	-0.86115	0.50231
C	-4.26503	-1.77843	0.27155
C	-4.74586	-1.99389	-1.01568
C	-4.20464	-1.29752	-2.09026
C	-3.17722	-0.38824	-1.86808
S	0.61829	-2.14278	-0.29084
S	-2.57021	-0.50794	2.10638
C	0.47979	-2.48808	-2.07826
C	-3.48694	-1.62208	3.20003
Cu	0.33637	0.20285	-0.19219
H	0.91604	5.87862	0.11819
H	2.91890	4.40177	0.29102
H	-1.34659	4.87271	-0.19691
H	-2.73833	0.15539	-2.69606
H	-4.57526	-1.46184	-3.09449
H	-5.54412	-2.70924	-1.17195
H	-4.70201	-2.32863	1.09258
H	-0.58334	-2.47267	-2.31599
H	1.00577	-1.73316	-2.65781
H	0.88449	-3.47864	-2.27619
H	-4.55361	-1.39984	3.20334
H	-3.31967	-2.66784	2.94363
H	-3.08658	-1.43621	4.19700
H	2.50773	-4.24072	-0.06744
H	4.95880	-4.30009	0.27277
H	6.20029	-2.15827	0.51321
H	5.01605	-0.01238	0.41390

2$^-$ Charge = [-1], Multiplicity = [1]

C	1.76110	4.00202	0.32666
C	1.66256	2.61687	0.20765
N	0.48099	2.03784	-0.00225
C	-0.63192	2.75285	-0.13555
C	-0.61310	4.14074	-0.02987
C	0.60505	4.76543	0.21273
C	2.88832	1.71189	0.27043
O	3.99967	2.24765	0.46112
N	2.57283	0.43168	0.10596
C	-1.90618	1.98019	-0.43879
O	-2.95333	2.62482	-0.62834
N	-1.74148	0.65292	-0.47118
C	3.46046	-0.60144	-0.03991
C	-2.85064	-0.14635	-0.80085
C	4.83143	-0.49671	-0.35947
C	5.61166	-1.62567	-0.57197
C	5.06177	-2.90006	-0.46701
C	3.71478	-3.03852	-0.14024
C	2.92284	-1.91552	0.06860
C	-3.55613	-0.84715	0.20288
C	-4.61381	-1.68818	-0.14978
C	-4.98767	-1.84010	-1.48205
C	-4.30317	-1.15043	-2.47631
C	-3.24700	-0.31492	-2.13032
S	1.20612	-2.02927	0.55071
S	-3.01655	-0.59183	1.87448
C	0.66938	-3.62553	-0.12716
C	-4.14588	-1.62525	2.84262
Cu	0.15153	-0.10156	-0.11126
H	2.72643	4.45634	0.49751
H	0.65459	5.84380	0.30355
H	-1.53007	4.70136	-0.14021
H	-2.70081	0.21965	-2.89874
H	-4.58611	-1.26227	-3.51617
H	-5.81057	-2.49792	-1.73432
H	-5.15721	-2.23082	0.61102
H	-5.18205	-1.30388	2.73813
H	-4.05298	-2.68054	2.58684
H	-3.84018	-1.48884	3.88047
H	-0.40710	-3.66433	0.03668
H	0.87695	-3.68709	-1.19421
H	1.13567	-4.45444	0.40224
H	3.29397	-4.03103	-0.04865
H	5.66976	-3.78126	-0.62991
H	6.66095	-1.50826	-0.81836
H	5.26124	0.49069	-0.43601

2^{2-} Charge = [-2], Multiplicity = [2]

C	1.81735	3.98502	0.36385
C	1.73567	2.58030	0.23993
N	0.49268	2.00260	-0.00152
C	-0.59618	2.76918	-0.13690
C	-0.56633	4.14786	-0.02847
C	0.69308	4.76989	0.23968
C	2.90910	1.71006	0.32187
O	4.06935	2.19880	0.47780
N	2.59416	0.38543	0.22911
C	-1.89596	2.03191	-0.43920
O	-2.93318	2.68990	-0.65455
N	-1.78304	0.69351	-0.43128
C	3.45054	-0.62988	-0.00130
C	-2.89971	-0.08089	-0.76095
C	4.83742	-0.55795	-0.31689
C	5.57987	-1.69576	-0.60050
C	4.99754	-2.96334	-0.58502
C	3.63991	-3.07747	-0.27086
C	2.88332	-1.95110	0.01253
C	-3.55473	-0.85023	0.23023
C	-4.62276	-1.67914	-0.11835
C	-5.06380	-1.75504	-1.43712
C	-4.43388	-0.99841	-2.41954
C	-3.36770	-0.17468	-2.07744
S	1.15475	-2.02247	0.46027
S	-2.93453	-0.68595	1.88627
C	0.60468	-3.62037	-0.20337
C	-4.01641	-1.77702	2.84642
Cu	0.15901	-0.04379	-0.11285
H	2.78745	4.42654	0.55613
H	0.76055	5.84732	0.33889
H	-1.47492	4.71915	-0.15183
H	-2.86656	0.40984	-2.83997
H	-4.76823	-1.04985	-3.44914
H	-5.89372	-2.40469	-1.68775
H	-5.12353	-2.27120	0.63502
H	-5.05775	-1.45724	2.80661
H	-3.93256	-2.81662	2.52970
H	-3.66495	-1.69657	3.87566
H	-0.47274	-3.64929	-0.04283
H	0.81303	-3.69804	-1.26979
H	1.06120	-4.45105	0.33312
H	3.18644	-4.06036	-0.25159
H	5.58002	-3.84912	-0.80609
H	6.63445	-1.59273	-0.83597
H	5.29500	0.42030	-0.32373

CO_2-2$^-$ Charge = [-1], Multiplicity = [3]

C	-1.07237	3.97835	0.09018
C	-0.94553	2.59551	0.18282
N	0.25121	2.03357	0.03699
C	1.36546	2.71366	-0.22773
C	1.31469	4.10044	-0.33448
C	0.07819	4.72880	-0.16566
C	-2.06556	1.60043	0.40987
O	-3.22776	2.00739	0.58102
N	-1.62719	0.32875	0.39125
C	2.59708	1.85067	-0.41639
O	3.68772	2.38764	-0.65936
N	2.30139	0.53279	-0.30422
C	-2.56381	-0.71859	0.54738
C	3.21872	-0.50537	-0.44702
C	-2.63315	-1.38693	1.77971
C	-3.50291	-2.45404	1.97922
C	-4.32430	-2.88716	0.93855
C	-4.26000	-2.24418	-0.29467
C	-3.39756	-1.16108	-0.50407
C	2.71365	-1.83122	-0.47076
C	3.55947	-2.93345	-0.56959
C	4.93764	-2.75604	-0.67061
C	5.45314	-1.46068	-0.66215
C	4.61836	-0.35241	-0.55148
S	-3.34360	-0.40006	-2.13464
S	0.93814	-2.12995	-0.35188
C	-4.87712	0.61014	-2.10808
C	0.47253	-2.23880	-2.12878
Cu	0.39497	0.10481	0.24971
C	0.92478	-0.21262	2.65877
O	1.33701	-1.34824	2.89156
O	0.72954	0.81922	3.29857
H	-2.04212	4.44260	0.20913
H	2.21510	4.66067	-0.54787
H	0.00954	5.80740	-0.24297
H	-1.98969	-1.04733	2.58270
H	-3.53644	-2.94753	2.94421
H	-5.00043	-3.72250	1.07998
H	-4.88265	-2.58486	-1.11417
H	3.13591	-3.93122	-0.56653
H	5.59316	-3.61497	-0.74923
H	6.52408	-1.30524	-0.73616
H	5.03106	0.64407	-0.54288
H	-4.90953	1.13905	-3.06161
H	-5.75730	-0.02602	-2.01782
H	-4.82670	1.32493	-1.28848
H	-0.61157	-2.34166	-2.16043
H	0.78163	-1.34039	-2.65893
H	0.94064	-3.12259	-2.55905

3 Charge = [0], Multiplicity = [2]

C	-1.01384	4.31438	0.02494
C	-1.01965	2.92736	-0.04620
N	0.12955	2.27128	0.00238
C	1.31964	2.84383	0.11332
C	1.40257	4.22740	0.19490
C	0.21626	4.95760	0.14683
C	-2.24053	2.03338	-0.13417
O	-3.36674	2.53366	-0.08960
N	-1.90269	0.72417	-0.19530
C	2.48353	1.87281	0.12244
O	3.63407	2.30127	0.22262
N	2.06665	0.58868	-0.01213
C	-2.86738	-0.28557	-0.13121
C	2.91073	-0.51497	-0.06413
C	-4.06462	-0.24496	-0.86336
C	-4.97744	-1.28671	-0.80228
C	-4.72823	-2.39911	-0.00187
C	-3.55542	-2.45586	0.73748
C	-2.62263	-1.42100	0.67246
C	2.39025	-1.72295	-0.58540
C	3.16565	-2.87563	-0.65505
C	4.47047	-2.87231	-0.18089
C	4.99828	-1.69568	0.34208
C	4.24131	-0.53452	0.39509
S	-1.13066	-1.49315	1.65697
S	0.73320	-1.72239	-1.26873
Cu	0.08152	0.33686	-0.19993
C	-0.31742	-3.01535	1.05757
C	-0.11345	-3.11696	-0.44388
H	-1.94172	4.86709	-0.01180
H	2.36339	4.71288	0.28919
H	0.25130	6.03802	0.20472
H	-4.26428	0.61413	-1.48660
H	-5.88979	-1.23022	-1.38381
H	-5.44288	-3.21060	0.05303
H	-3.36113	-3.30972	1.37450
H	2.75047	-3.78021	-1.08129
H	5.06655	-3.77449	-0.22787
H	6.01606	-1.67766	0.71311
H	4.66470	0.37167	0.79625
H	-0.89149	-3.88465	1.38104
H	0.63398	-3.03235	1.59138
H	-1.06590	-3.17867	-0.97162
H	0.44430	-4.02486	-0.67490

3^{-} Charge = [-1], Multiplicity = [1]

C	1.77513	4.10913	-0.09031
C	1.61284	2.72637	-0.13671
N	0.39583	2.20281	-0.26597
C	-0.70698	2.94256	-0.26460
C	-0.62383	4.33201	-0.23116
C	0.63996	4.91197	-0.15857
C	2.76566	1.73521	0.00779
O	3.93460	2.16912	-0.03263
N	2.31872	0.48958	0.15574
C	-2.02477	2.18135	-0.17901
O	-3.08259	2.83396	-0.21975
N	-1.86800	0.85542	-0.02874
C	3.07554	-0.63287	0.37023
C	-2.91915	0.05246	0.40011
C	4.32853	-0.65843	1.02095
C	4.97638	-1.85269	1.29730
C	4.41580	-3.07249	0.92107
C	3.19767	-3.08017	0.25114
C	2.52632	-1.89079	-0.01727
C	-3.13712	-1.24837	-0.12009
C	-4.11551	-2.07777	0.43860
C	-4.89090	-1.66598	1.51080
C	-4.70363	-0.38002	2.01881
C	-3.75012	0.45735	1.46913
S	1.01455	-1.87915	-0.97914
S	-2.26691	-1.82158	-1.57209
Cu	0.09792	0.13517	-0.31740
C	-0.10349	-3.07985	-0.14953
C	-1.34660	-3.30563	-1.00368
H	2.76353	4.53535	0.00881
H	-1.52326	4.93104	-0.24321
H	0.73840	5.99036	-0.12813
H	4.77223	0.28395	1.30783
H	5.93277	-1.83279	1.80760
H	4.92526	-4.00446	1.13152
H	2.77134	-4.02231	-0.07127
H	-4.27240	-3.06101	0.01132
H	-5.63802	-2.32722	1.93171
H	-5.30476	-0.03039	2.85046
H	-3.61026	1.44887	1.87666
H	-0.35644	-2.69646	0.83778
H	0.41476	-4.03276	-0.03666
H	-2.03018	-3.96168	-0.46362
H	-1.07727	-3.80818	-1.93487

3^{2-} Charge = [-2], Multiplicity = [2]

C	1.81735	3.98502	0.36385
C	1.73567	2.58030	0.23993
N	0.49268	2.00260	-0.00152
C	-0.59618	2.76918	-0.13690
C	-0.56633	4.14786	-0.02847
C	0.69308	4.76989	0.23968
C	2.90910	1.71006	0.32187
O	4.06935	2.19880	0.47780
N	2.59416	0.38543	0.22911
C	-1.89596	2.03191	-0.43920
O	-2.93318	2.68990	-0.65455
N	-1.78304	0.69351	-0.43128
C	3.45054	-0.62988	-0.00130
C	-2.89971	-0.08089	-0.76095
C	4.83742	-0.55795	-0.31689
C	5.57987	-1.69576	-0.60050
C	4.99754	-2.96334	-0.58502
C	3.63991	-3.07747	-0.27086
C	2.88332	-1.95110	0.01253
C	-3.55473	-0.85023	0.23023
C	-4.62276	-1.67914	-0.11835
C	-5.06380	-1.75504	-1.43712
C	-4.43388	-0.99841	-2.41954
C	-3.36770	-0.17468	-2.07744
S	1.15475	-2.02247	0.46027
S	-2.93453	-0.68595	1.88627
C	0.60468	-3.62037	-0.20337
C	-4.01641	-1.77702	2.84642
Cu	0.15901	-0.04379	-0.11285
H	2.78745	4.42654	0.55613
H	0.76055	5.84732	0.33889
H	-1.47492	4.71915	-0.15183
H	-2.86656	0.40984	-2.83997
H	-4.76823	-1.04985	-3.44914
H	-5.89372	-2.40469	-1.68775
H	-5.12353	-2.27120	0.63502
H	-5.05775	-1.45724	2.80661
H	-3.93256	-2.81662	2.52970
H	-3.66495	-1.69657	3.87566
H	-0.47274	-3.64929	-0.04283
H	0.81303	-3.69804	-1.26979
H	1.06120	-4.45105	0.33312
H	3.18644	-4.06036	-0.25159
H	5.58002	-3.84912	-0.80609
H	6.63445	-1.59273	-0.83597
H	5.29500	0.42030	-0.32373

CO_2-3$^-$ Charge = [-1], Multiplicity = [3]

C	0.74602	4.27722	-0.75812
C	0.80165	2.91110	-0.51428
N	-0.30982	2.24279	-0.23571
C	-1.51338	2.80801	-0.19914
C	-1.64222	4.17268	-0.41892
C	-0.49356	4.90755	-0.69881
C	2.06246	2.08225	-0.50354
O	3.14663	2.63949	-0.71137
N	1.83423	0.77354	-0.25174
C	-2.65918	1.85208	0.02789
O	-3.81530	2.28086	0.10755
N	-2.22966	0.57378	0.06176
C	2.93706	-0.02244	0.10494
C	-3.07857	-0.52679	0.14045
C	3.79839	0.41420	1.12952
C	4.87807	-0.34011	1.55238
C	5.14420	-1.57376	0.96161
C	4.30361	-2.03193	-0.03983
C	3.20054	-1.28342	-0.46729
C	-2.61823	-1.72814	-0.44476
C	-3.38688	-2.88512	-0.40789
C	-4.62031	-2.88131	0.23645
C	-5.08370	-1.71007	0.82578
C	-4.32844	-0.54449	0.77700
S	2.15049	-1.99750	-1.72123
S	-1.06389	-1.60193	-1.31033
C	1.14898	-3.12966	-0.66130
C	-0.28429	-3.25370	-1.14187
Cu	-0.18250	0.30903	0.04264
C	0.04545	-0.51440	2.25387
O	0.19244	-1.72875	2.36707
O	-0.01683	0.45439	3.00471
H	-2.61865	4.63432	-0.38198
H	-0.56595	5.97251	-0.87944
H	1.65209	4.82104	-0.98348
H	-4.69439	0.36191	1.23347
H	-6.04241	-1.69981	1.33029
H	-5.21292	-3.78686	0.26833
H	-3.03507	-3.79075	-0.88428
H	-0.85689	-3.83380	-0.41990
H	-0.34998	-3.74665	-2.11150
H	1.16219	-2.72286	0.34766
H	1.60978	-4.11865	-0.65674
H	3.59826	1.36858	1.59707
H	5.51267	0.03326	2.34742
H	5.99024	-2.17025	1.27929
H	4.49312	-2.99038	-0.50653

References

(1) Bergbreiter, D. E.; Killough, J. M. Reactions of potassium-graphite. *J. Am. Chem. Soc.* **1978**, *100* (7), 2126–2134.
(2) Zhang, X.; Huang, D.; Chen, Y.-S.; Holm, R. H. Synthesis of Binucleating Macrocycles and Their Nickel(II) Hydroxo- and Cyano-Bridged Complexes with Divalent Ions: Anatomical Variation of Ligand Features. *Inorg. Chem.* **2012**, *51* (20), 11017–11029.
(3) Donoghue, P. J.; Tehranchi, J.; Cramer, C. J.; Sarangi, R.; Solomon, E. I.; Tolman, W. B. Rapid C–H Bond Activation by a Monocopper(III)–Hydroxide Complex. *J. Am. Chem. Soc.* **2011**, *133* (44), 17602–17605.
(4) Liu, W.-J.; Huang, H.-H.; Ouyang, T.; Jiang, L.; Zhong, D.-C.; Zhang, W.; Lu, T.-B. A Copper(II) Molecular Catalyst for Efficient and Selective Photochemical Reduction of CO_2 to CO in a Water-Containing System. *Chem. Eur. J.* **2018**, *24* (18), 4503–4508.
(5) Azcarate, I.; Costentin, C.; Robert, M.; Savéant, J.-M. Through-Space Charge Interaction Substituent Effects in Molecular Catalysis Leading to the Design of the Most Efficient Catalyst of CO_2-to-CO Electrochemical Conversion. *J. Am. Chem. Soc.* **2016**, *138* (51), 16639–16644.
(6) Costentin, C.; Drouet, S.; Robert, M.; Savéant, J.-M. A Local Proton Source Enhances CO_2 Electroreduction to CO by a Molecular Fe Catalyst. *Science* **2012**, *338* (6103), 90–94.
(7) Costentin, C.; Passard, G.; Robert, M.; Saveant, J. M. Ultraefficient homogeneous catalyst for the CO_2-to-CO electrochemical conversion. *Proc Natl Acad Sci U S A* **2014**, *111* (42), 14990–14994.
(8) Sampson, M. D.; Nguyen, A. D.; Grice, K. A.; Moore, C. E.; Rheingold, A. L.; Kubiak, C. P. Manganese Catalysts with Bulky Bipyridine Ligands for the Electrocatalytic Reduction of Carbon Dioxide: Eliminating Dimerization and Altering Catalysis. *J. Am. Chem. Soc.* **2014**, *136* (14), 5460–5471.
(9) Costentin, C.; Drouet, S.; Robert, M.; Saveant, J. M. Turnover numbers, turnover frequencies, and overpotential in molecular catalysis of electrochemical reactions. Cyclic voltammetry and preparative-scale electrolysis. *J. Am. Chem. Soc.* **2012**, *134* (27), 11235–11242.
(10) Cometto, C.; Chen, L.; Lo, P.-K.; Guo, Z.; Lau, K.-C.; Anxolabéhère-Mallart, E.; Fave, C.; Lau, T.-C.; Robert, M. Highly Selective Molecular Catalysts for the CO_2-to-CO Electrochemical Conversion at Very Low Overpotential. Contrasting Fe vs Co Quaterpyridine Complexes upon Mechanistic Studies. *ACS Catal.* **2018**, *8* (4), 3411–3417.
(11) Froehlich, J. D.; Kubiak, C. P. Homogeneous CO_2 Reduction by Ni(cyclam) at a Glassy Carbon Electrode. *Inorg. Chem.* **2012**, *51* (7), 3932–3934.
(12) Stoll, S.; Schweiger, A. EasySpin, a comprehensive software package for spectral simulation and analysis in EPR. *J. Magn. Reson.* **2006**, *178* (1), 42–55.
(13) *Apex6*; Bruker AXS Inc.: 2024.
(14) Dolomanov, O. V.; Bourhis, L. J.; Gildea, R. J.; Howard, J. A. K.; Puschmann, H. OLEX2: a complete structure solution, refinement and analysis program. *J. Appl. Crystallogr.* **2009**, *42* (2), 339–341.
(15) Sheldrick, G. SHELXT - Integrated space-group and crystal-structure determination. *Acta Crystallographica Section A* **2015**, *71* (1), 3–8.
(16) Sheldrick, G. Crystal structure refinement with SHELXL. *Acta Crystallographica Section C* **2015**, *71* (1), 3–8.
(17) Solis, B. H.; Hammes-Schiffer, S. Proton-Coupled Electron Transfer in Molecular Electrocatalysis: Theoretical Methods and Design Principles. *Inorg. Chem.* **2014**, *53* (13), 6427–6443.

(18) Isse, A. A.; Gennaro, A. Absolute Potential of the Standard Hydrogen Electrode and the Problem of Interconversion of Potentials in Different Solvents. *J. Phys. Chem. B* **2010**, *114* (23), 7894–7899.
(19) Kégl, T. R.; Carrilho, R. M. B.; Kégl, T. Theoretical insights into the electronic structure of nickel(0)-diphosphine-carbon dioxide complexes. *J. Organomet. Chem.* **2020**, *924*, 121462.
(20) Mitoraj, M. P.; Michalak, A.; Ziegler, T. A Combined Charge and Energy Decomposition Scheme for Bond Analysis. *J. Chem. Theory Comput.* **2009**, *5* (4), 962–975.
(21) Chauhan, A.; Karnamkkott, H. S.; Gorantla, S. M. N. V. T.; Mondal, K. C. Dinitrogen Binding and Activation: Bonding Analyses of Stable V(III/I)–N_2–V(III/I) Complexes by the EDA–NOCV Method from the Perspective of Vanadium Nitrogenase. *ACS Omega* **2022**, *7* (35), 31577–31590.
(22) Neese, F. The ORCA program system. *WIREs Comput. Mol. Sci.* **2012**, *2* (1), 73–78.
(23) Suthar, S.; Kaushik, R.; Mondal, K. C. Probing Transition Metal–Fullerene Bonds: EDA–NOCV Analyses. *Organometallics* **2025**, *44* (13), 1395–1403.
(24) Biesinger, M. C. Advanced analysis of copper X-ray photoelectron spectra. *Surf. Interface Anal.* **2017**, *49* (13), 1325–1334.
